# Supplementary material for: [Au]/[Ag]-catalysed expedient synthesis of branched heneicosafuranosyl arabinogalactan motif of Mycobacterium tuberculosis cell wall
Source: Nat Commun. 2017 Jan 25;8:14019. doi: 10.1038/ncomms14019 (PMC5288502; doi:10.1038/ncomms14019)
Supplement: Supplementary Information — Supplementary figures, supplementary methods and supplementary references. [file ncomms14019-s1.pdf]

Supplementary Figure 1. ^{1}H NMR Spectrum (399.78 MHz, $CDCl_3$) of Compound **8b**

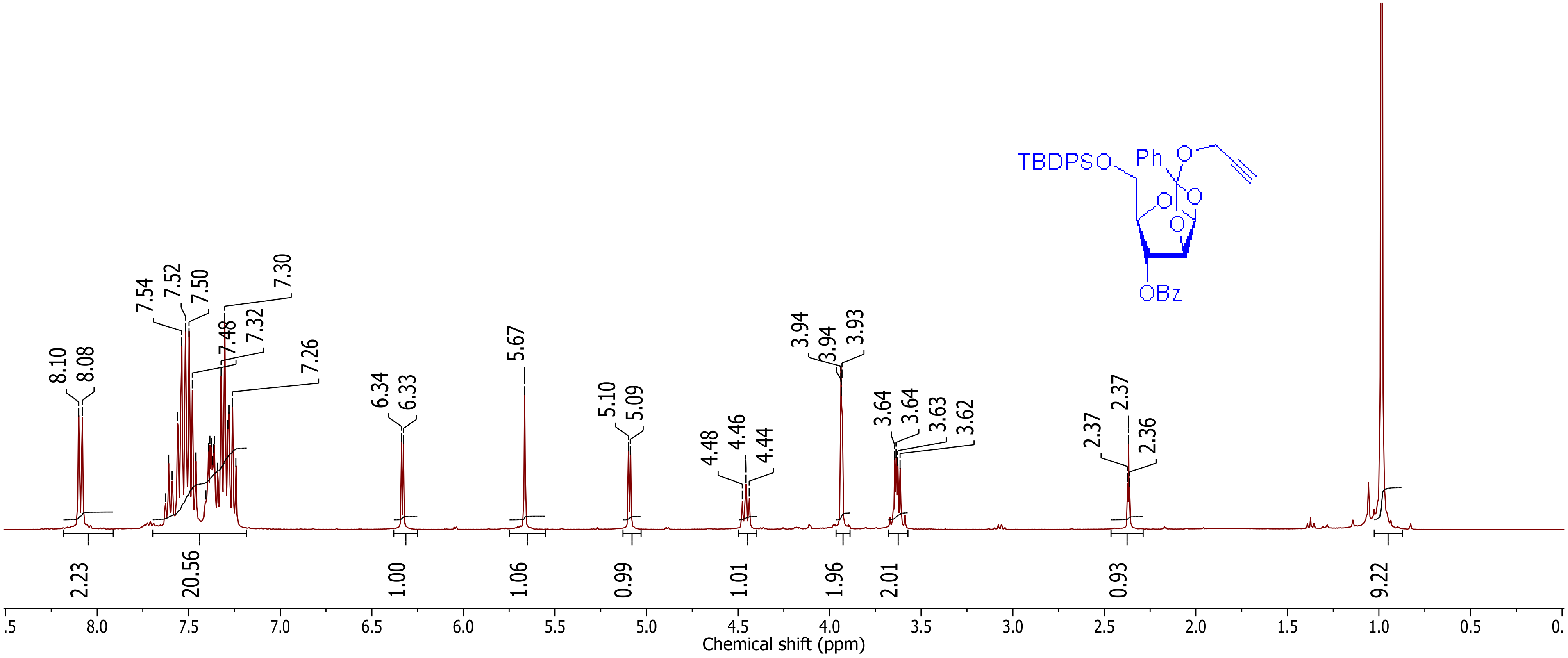

Supplementary Figure 2. ^{13}C NMR Spectrum (100.53 MHz, $CDCl_3$) of Compound **8b**

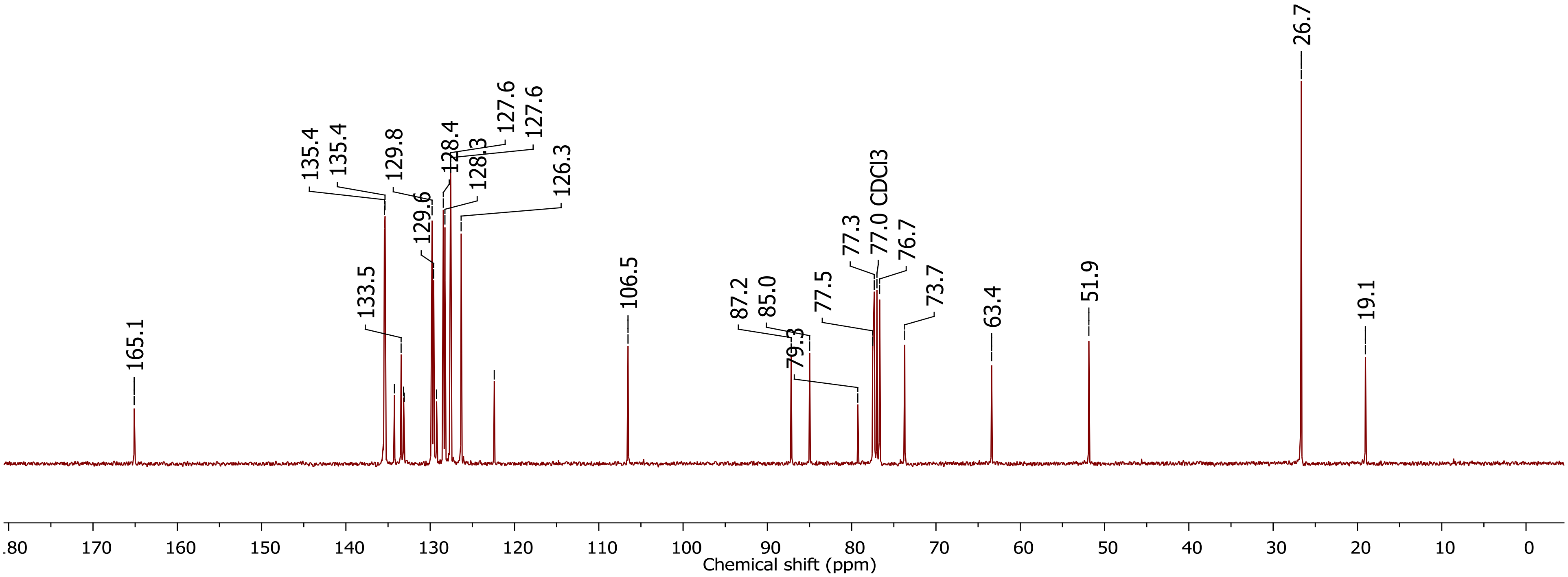

Supplementary Figure 3. DEPT NMR Spectrum (100.53 MHz, $CDCl_3$) of Compound **8b**

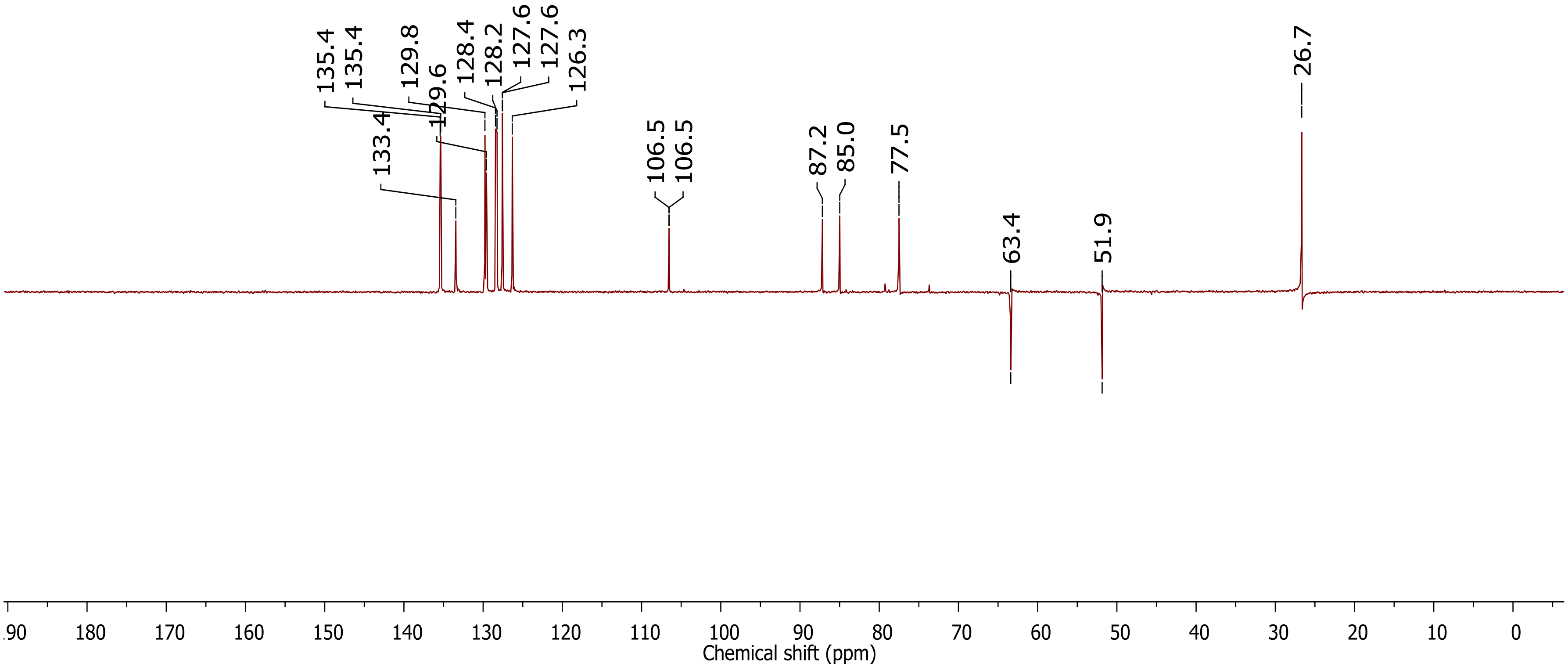

Supplementary Figure 4. ^{1}H NMR Spectrum (399.78 MHz, $CDCl_3$) of Compound **8a**

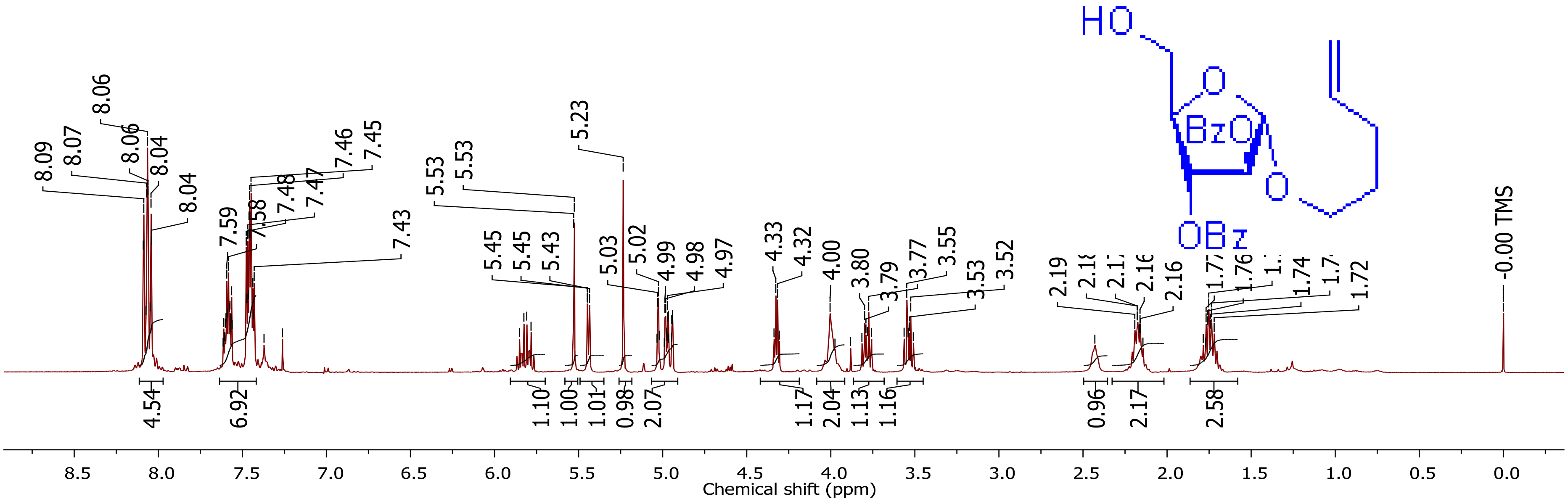

Supplementary Figure 5. ^{13}C NMR Spectrum (100.53 MHz, $CDCl_3$) of Compound **8a**

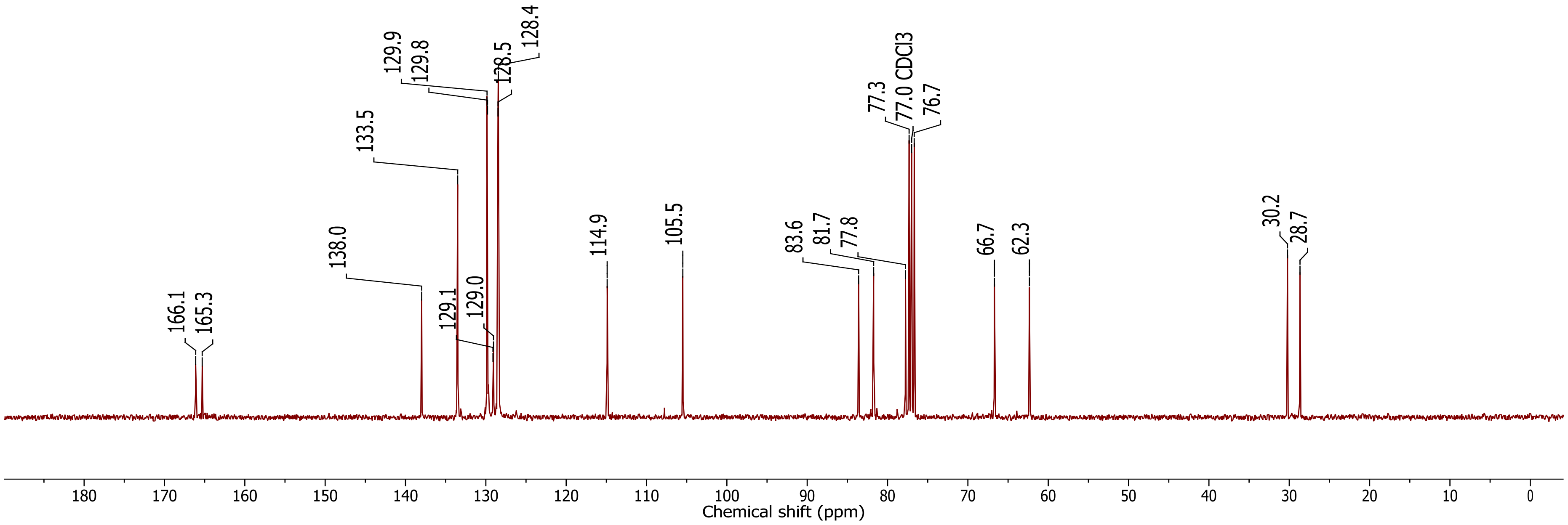

Supplementary Figure 6. DEPT NMR Spectrum (100.53 MHz, $CDCl_3$) of Compound **8a**

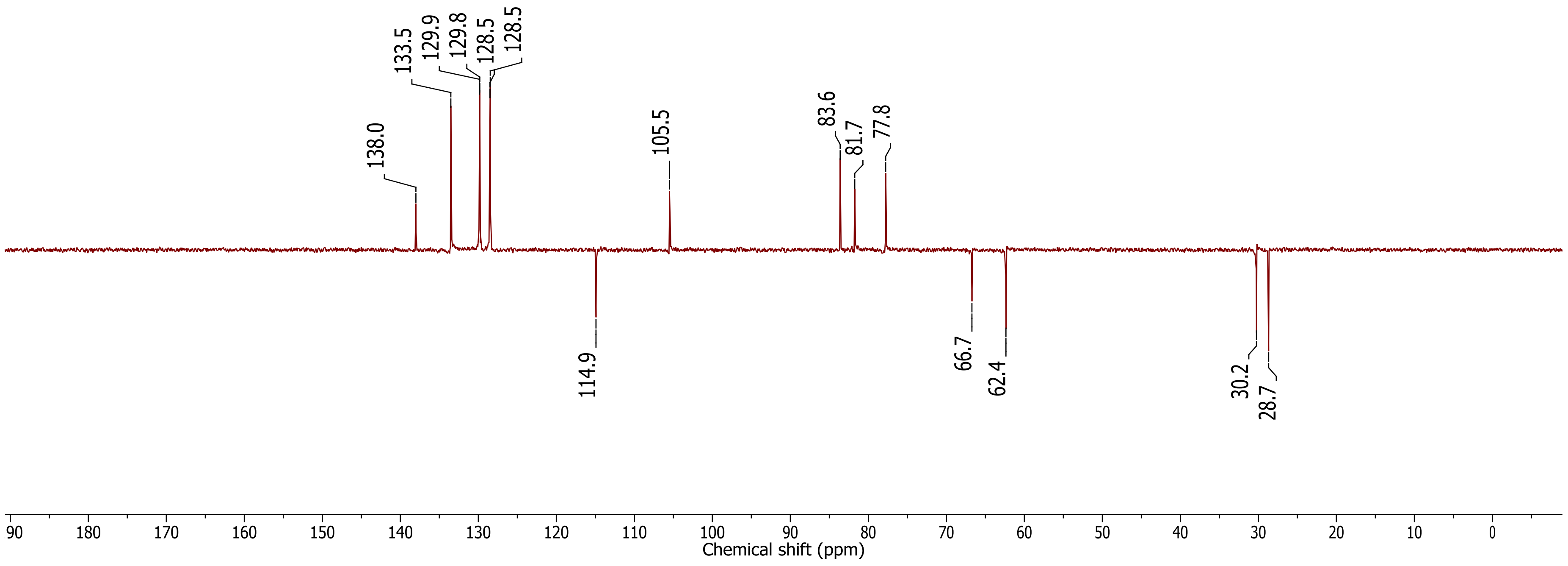

Supplementary Figure 7. ^{1}H NMR Spectrum (399.78 MHz, $CDCl_3$) of Compound **8d**

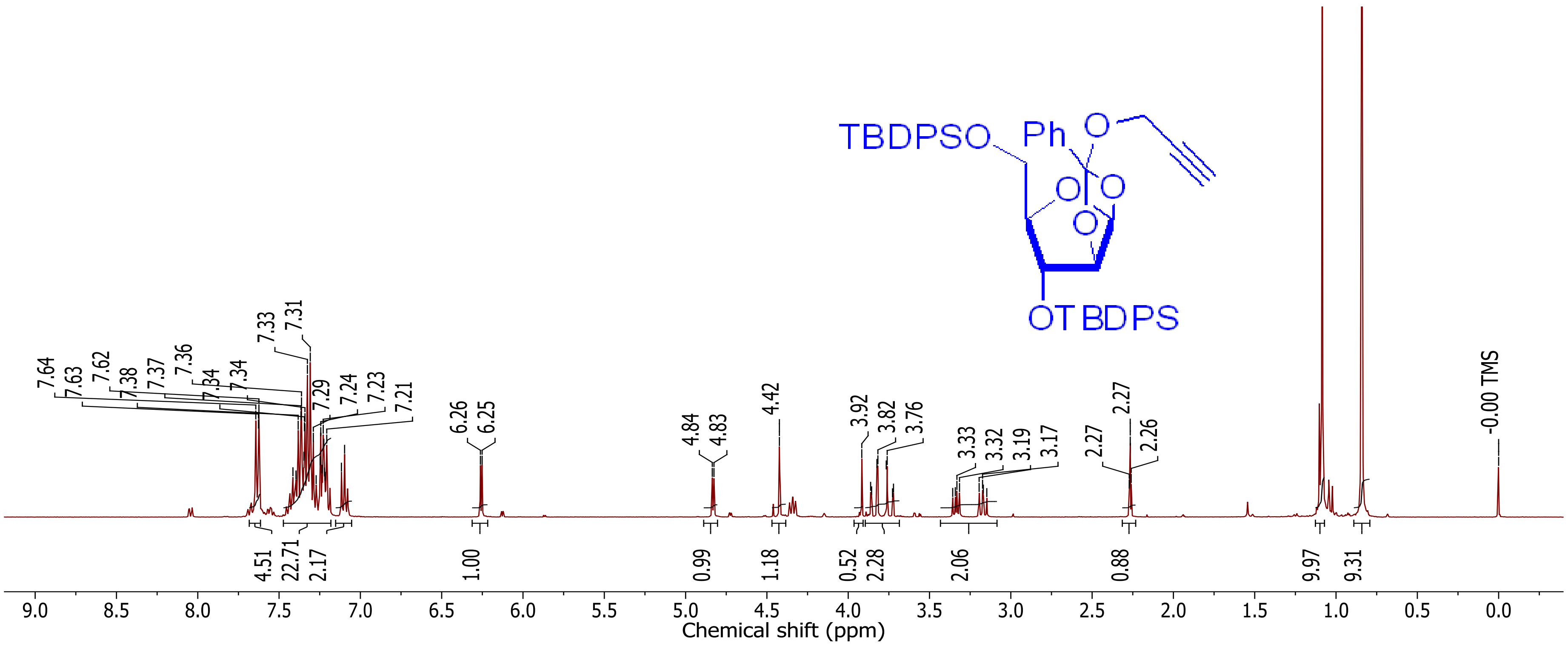

Supplementary Figure 8. ^{13}C NMR Spectrum (100.53 MHz, $CDCl_3$) of Compound **8d**

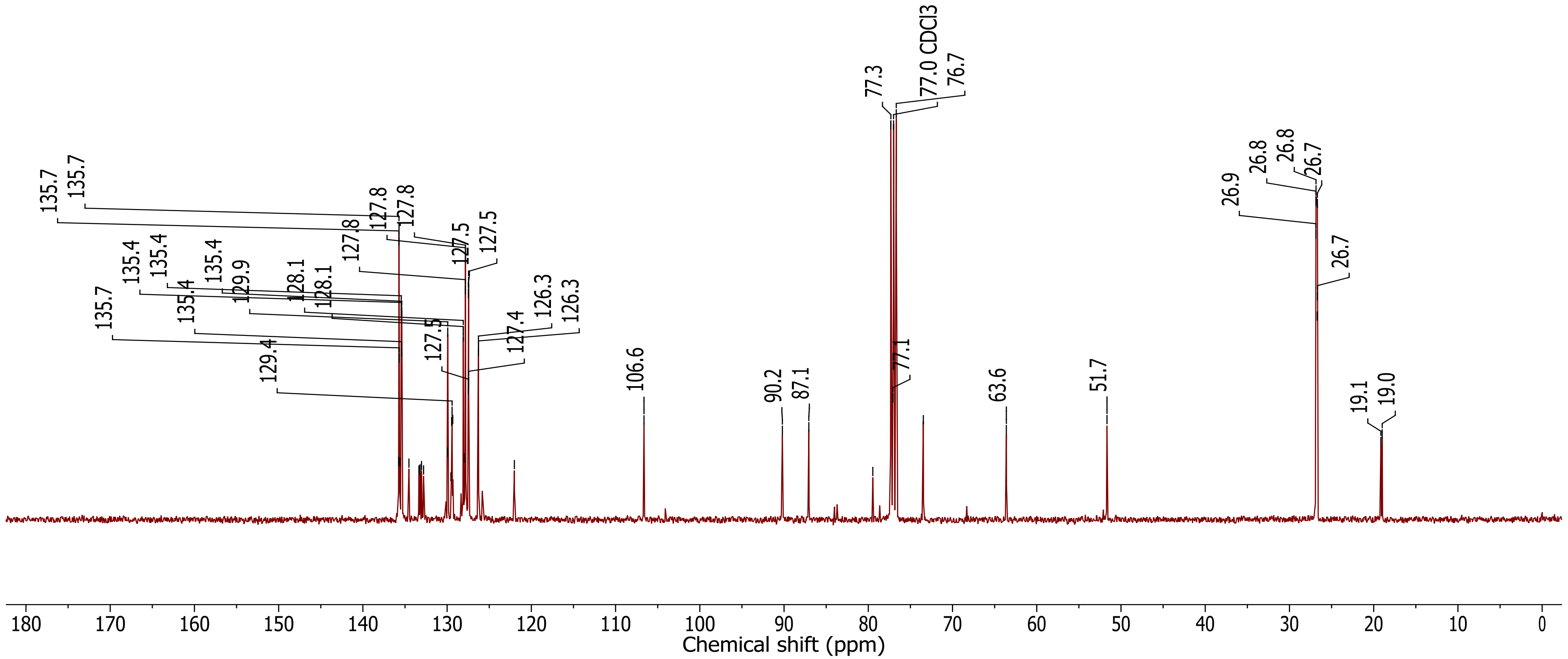

Supplementary Figure 9. DEPT NMR Spectrum (100.53 MHz, $CDCl_3$) of Compound **8d**

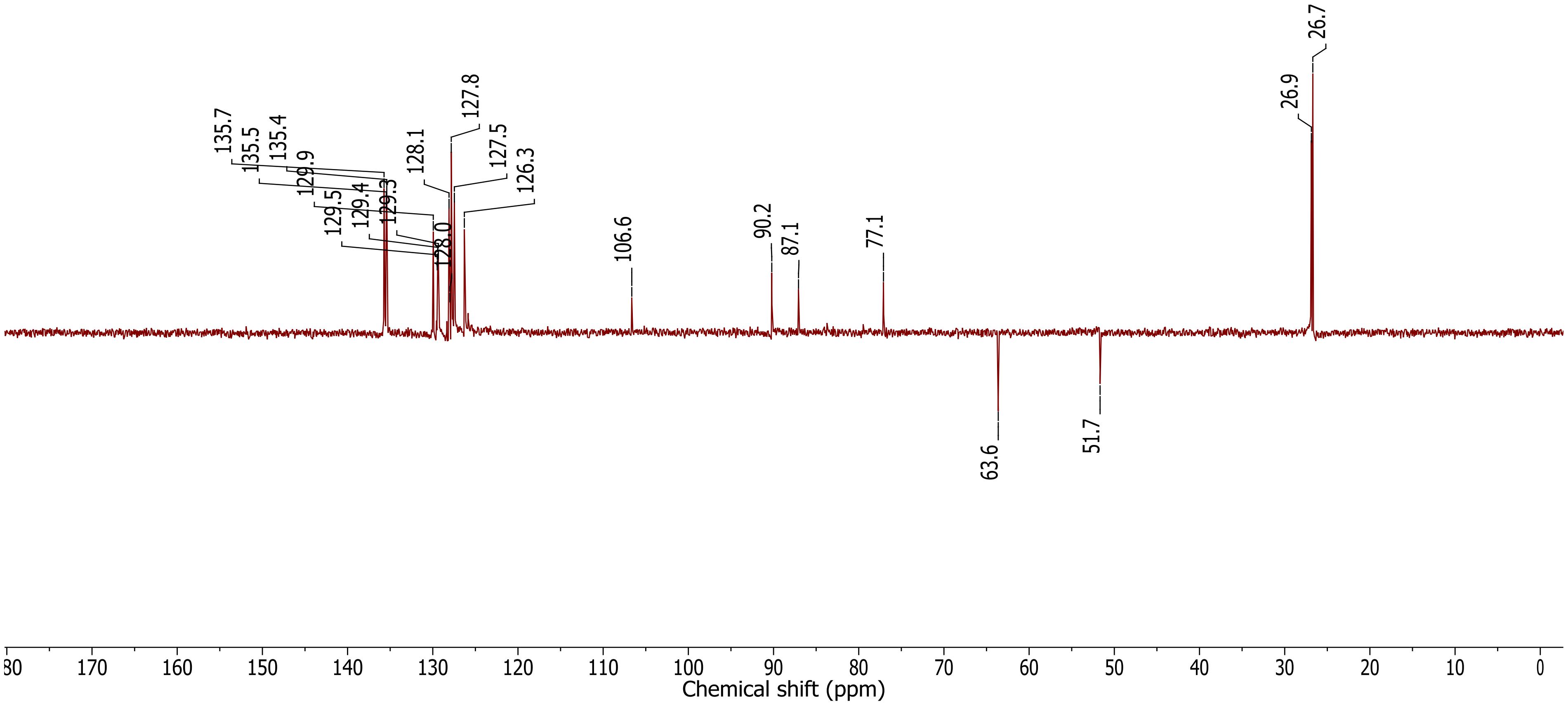

Supplementary Figure 10. ^{1}H NMR Spectrum (399.78 MHz, $CDCl_3$) of Compound **9**

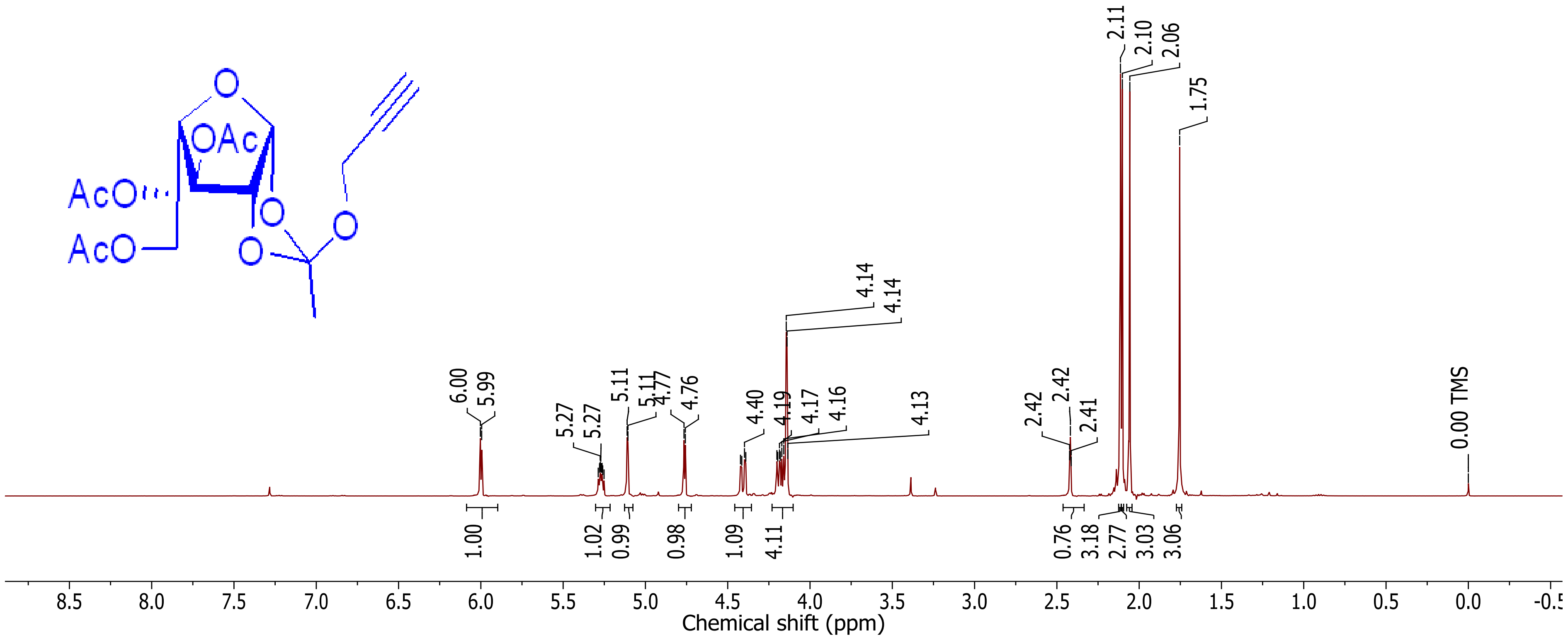

Supplementary Figure 11. ^{13}C NMR Spectrum (100.53 MHz, $CDCl_3$) of Compound **9**

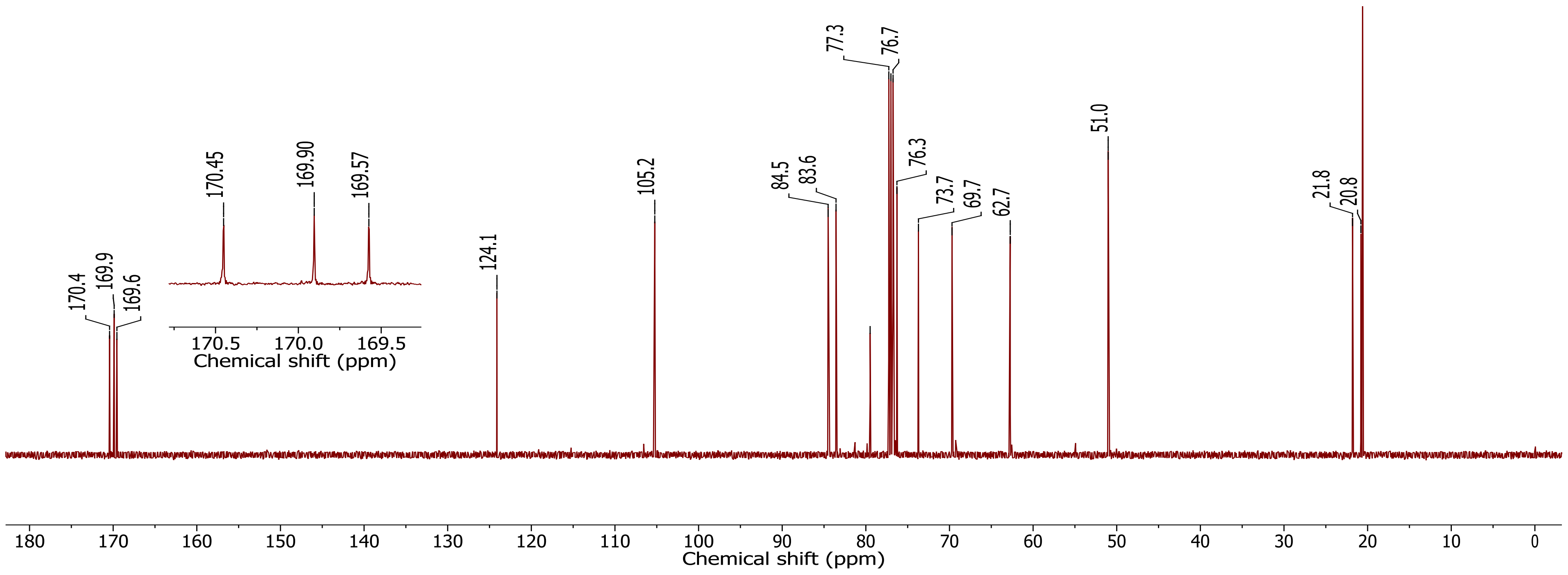

Supplementary Figure 12. DEPT NMR Spectrum (100.53 MHz, $CDCl_3$) of Compound **9**

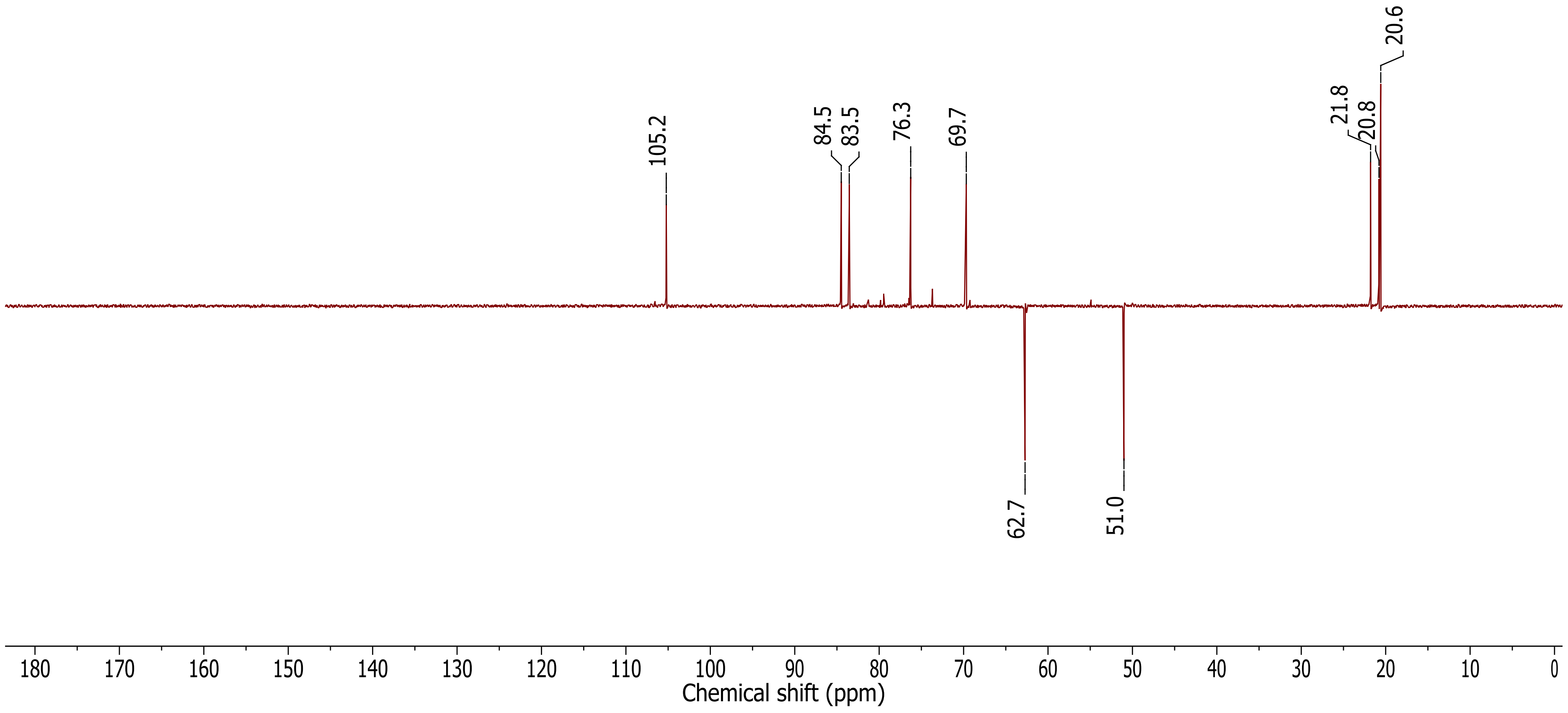

Supplementary Figure 13. ^{1}H NMR Spectrum (399.78 MHz, $CDCl_3$) of Compound **18**

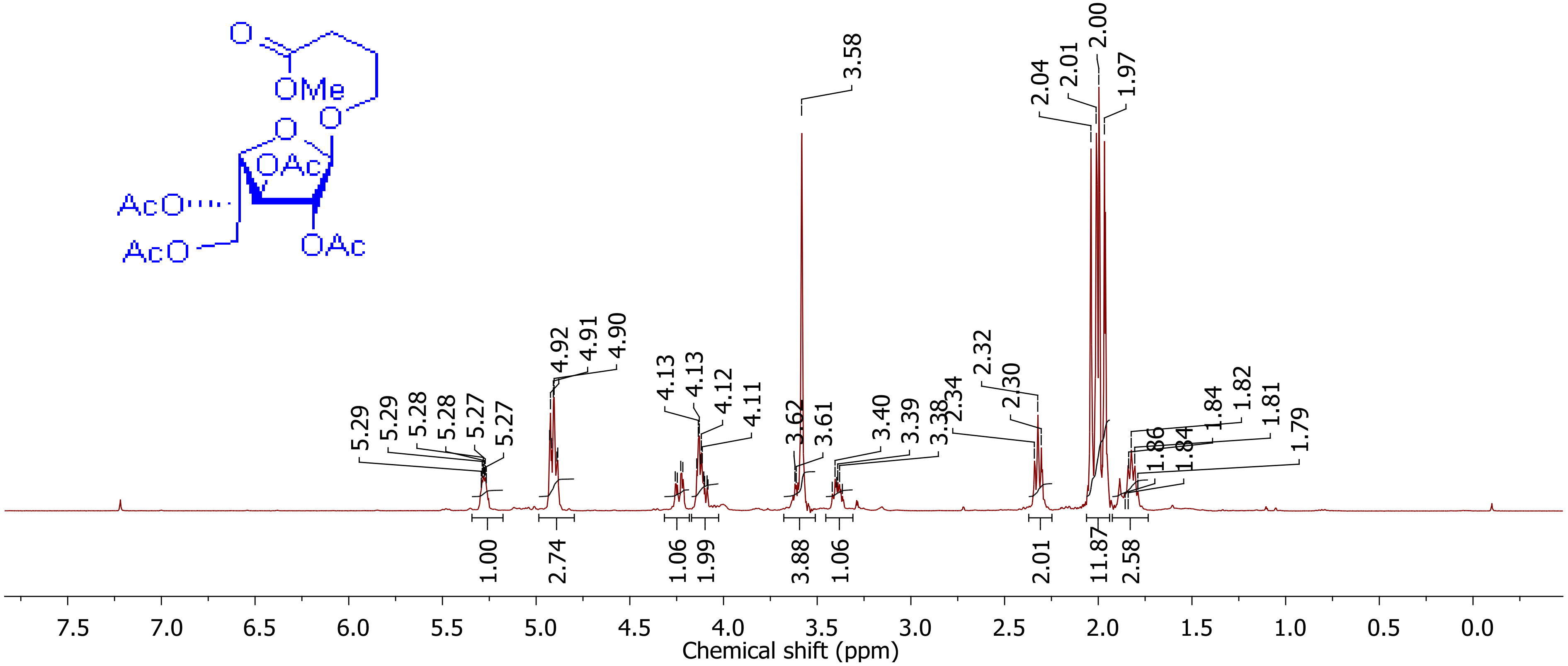

Supplementary Figure 14. ^{13}C NMR Spectrum (100.53 MHz, $CDCl_3$) of Compound **18**

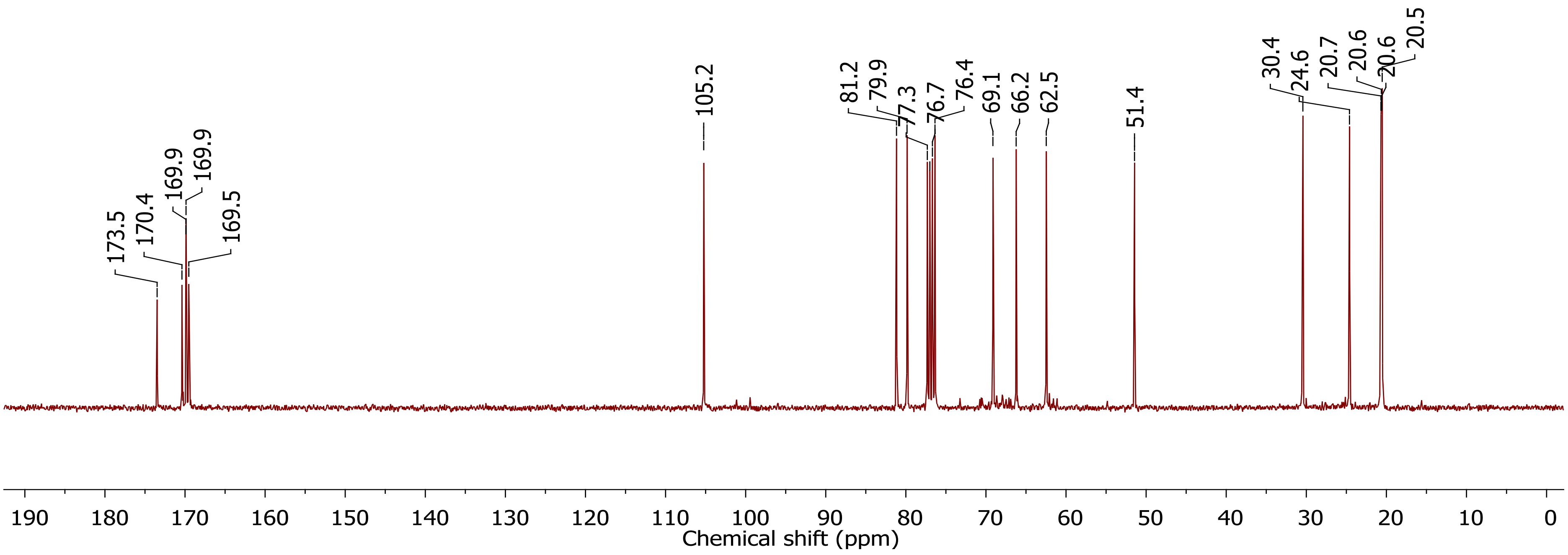

Supplementary Figure 15. DEPT NMR Spectrum (100.53 MHz, $CDCl_3$) of Compound **18**

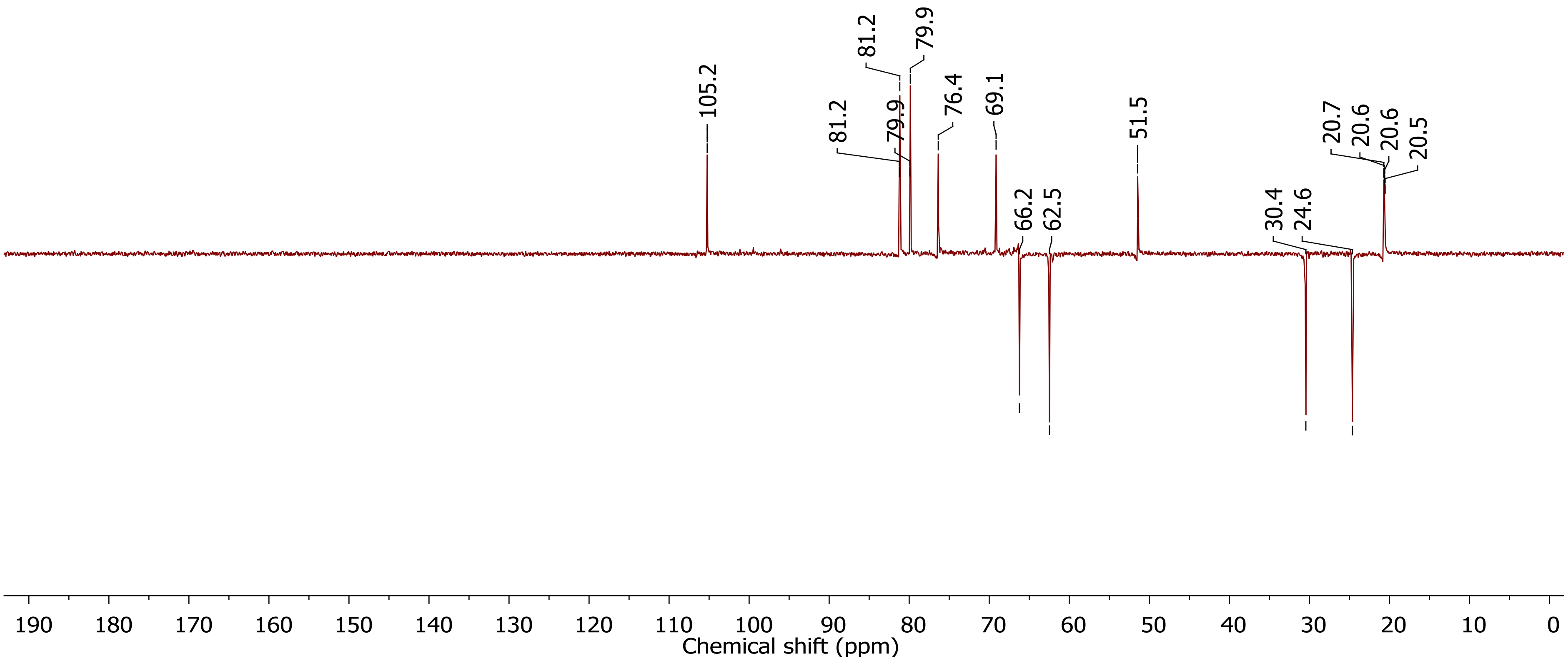

Supplementary Figure 16. ^{1}H NMR Spectrum (399.78 MHz, $CDCl_3$) of Compound **10**

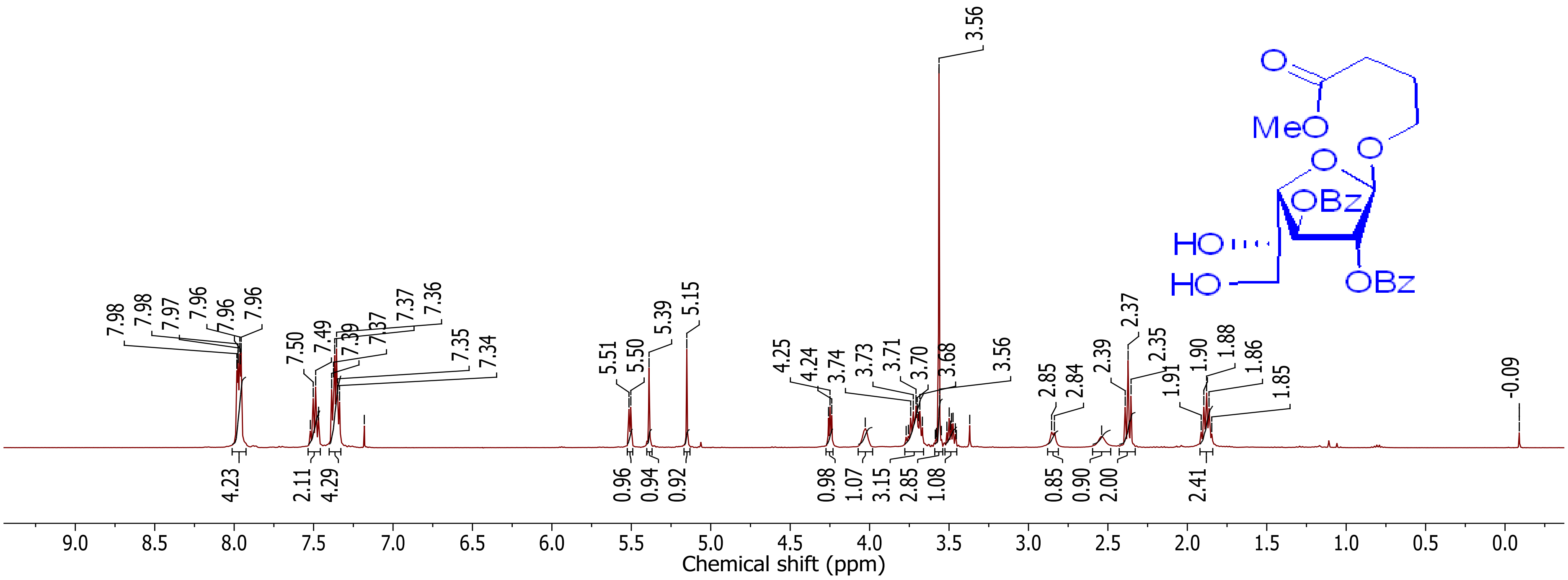

Supplementary Figure 17. ^{13}C NMR Spectrum (100.53 MHz, $CDCl_3$) of Compound **10**

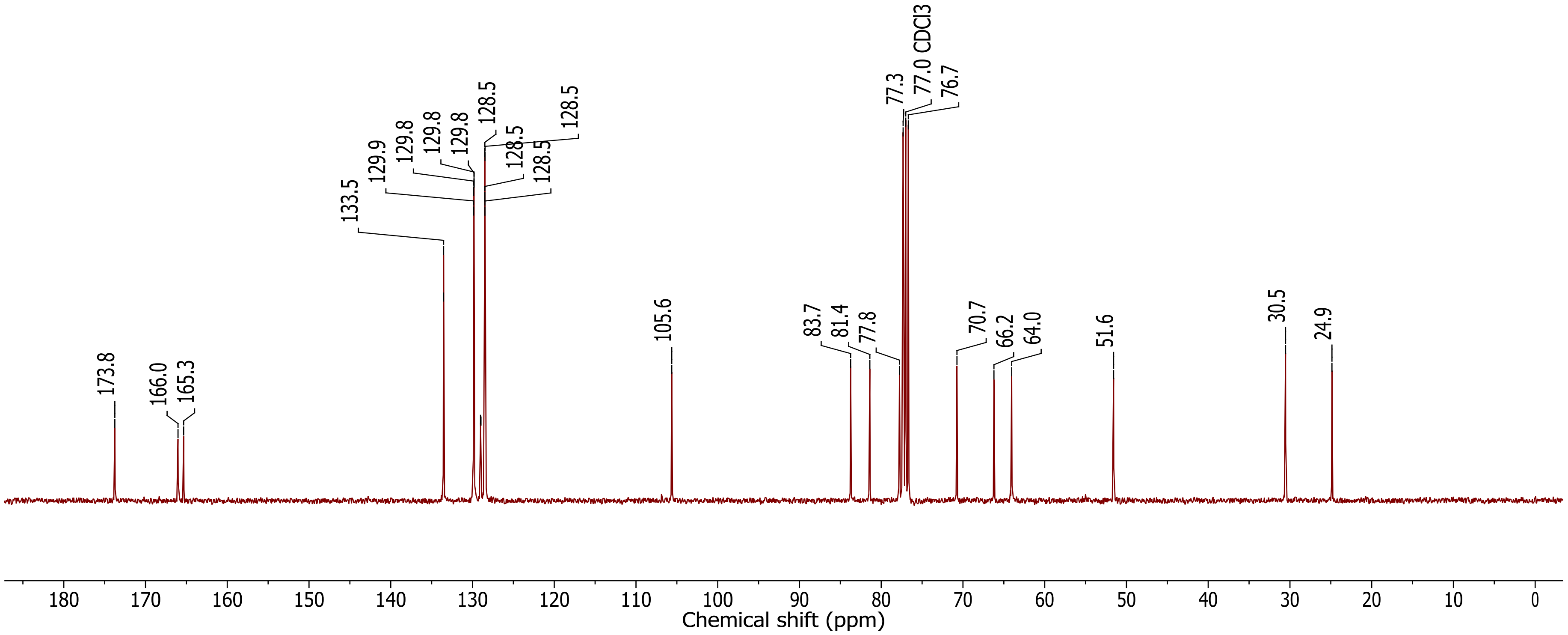

Supplementary Figure 18. DEPT NMR Spectrum (100.53 MHz, $CDCl_3$) of Compound **10**

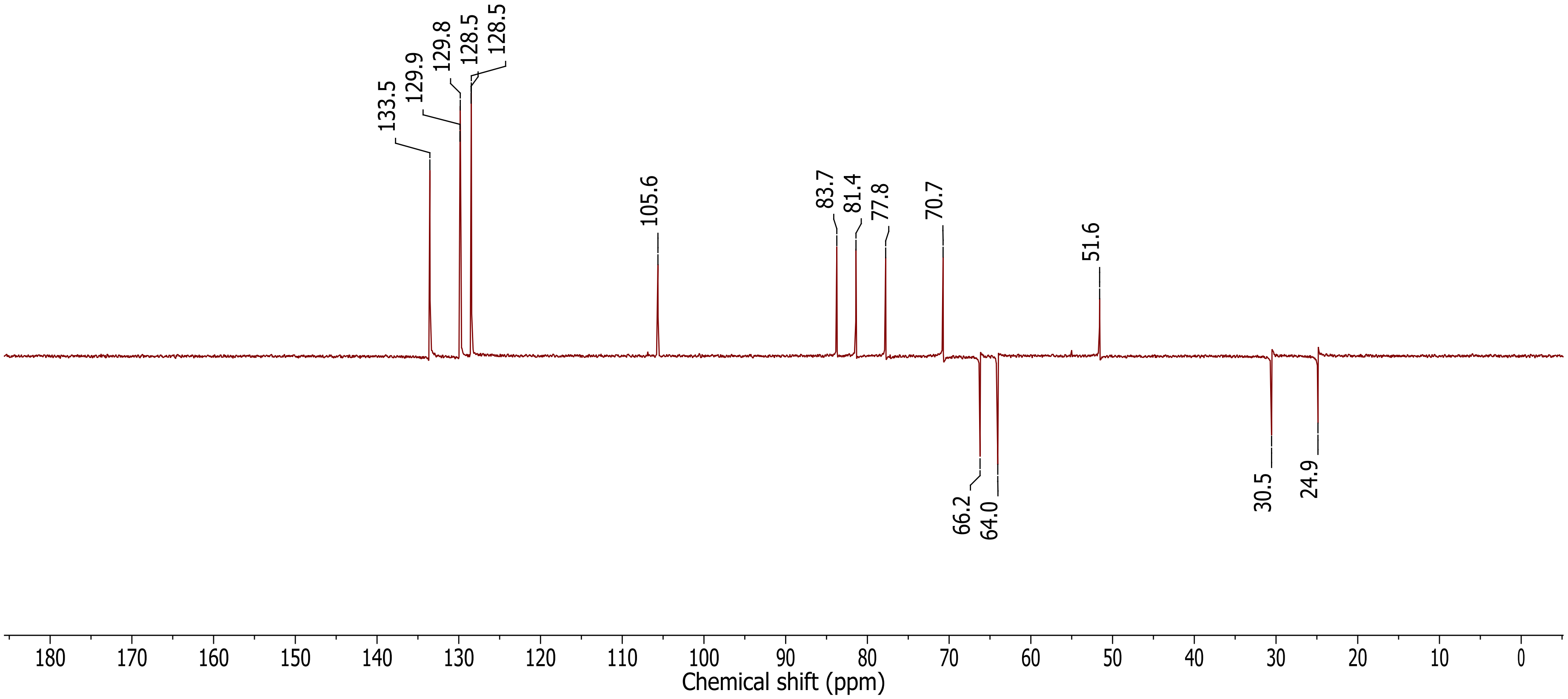

Supplementary Figure 19. ^{1}H NMR Spectrum (399.78 MHz, $CDCl_3$) of Compound **19**

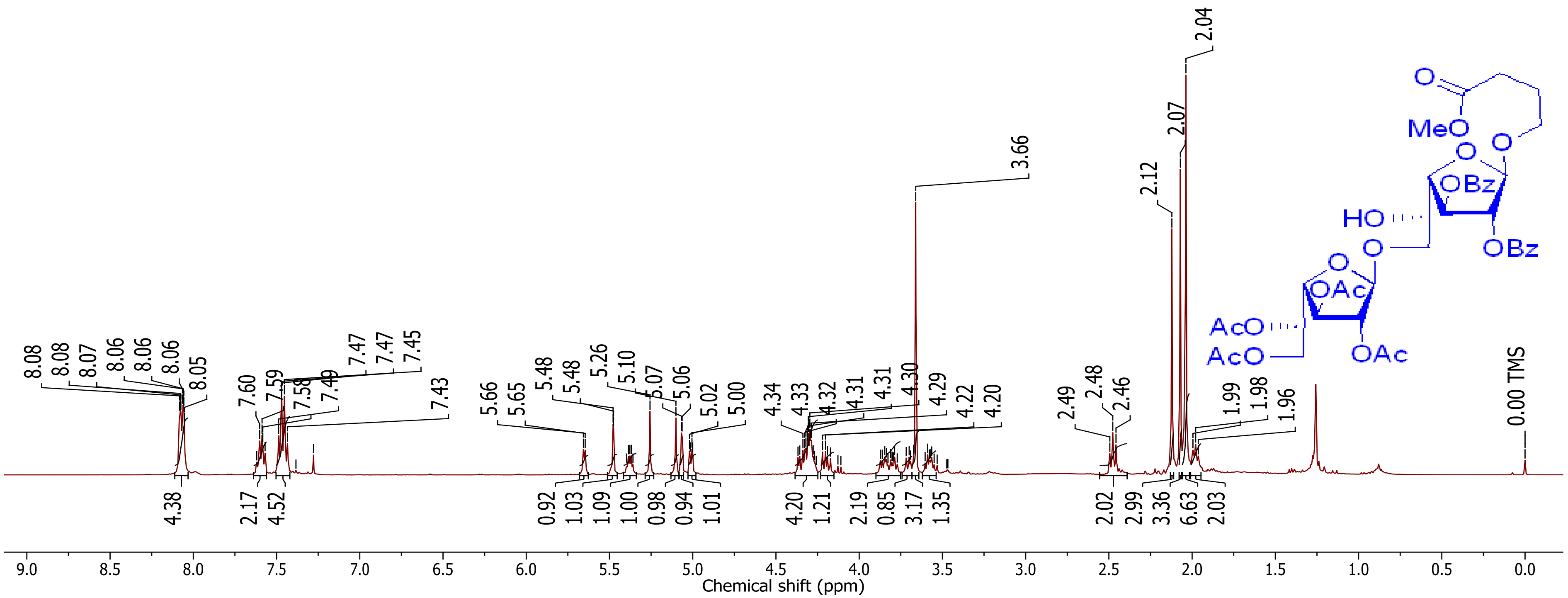

Supplementary Figure 20. ^{13}C NMR Spectrum (100.53 MHz, $CDCl_3$) of Compound **19**

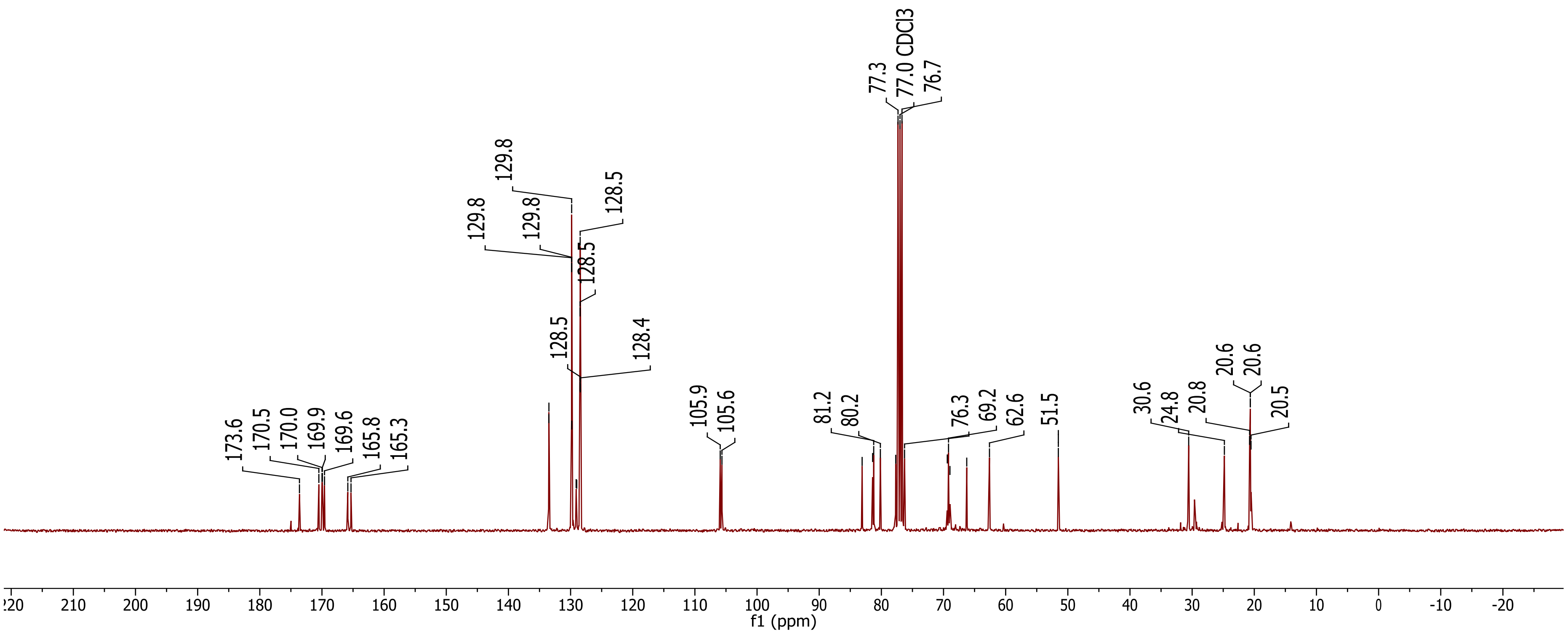

Supplementary Figure 21. DEPT NMR Spectrum (100.53 MHz, $CDCl_3$) of Compound **19**

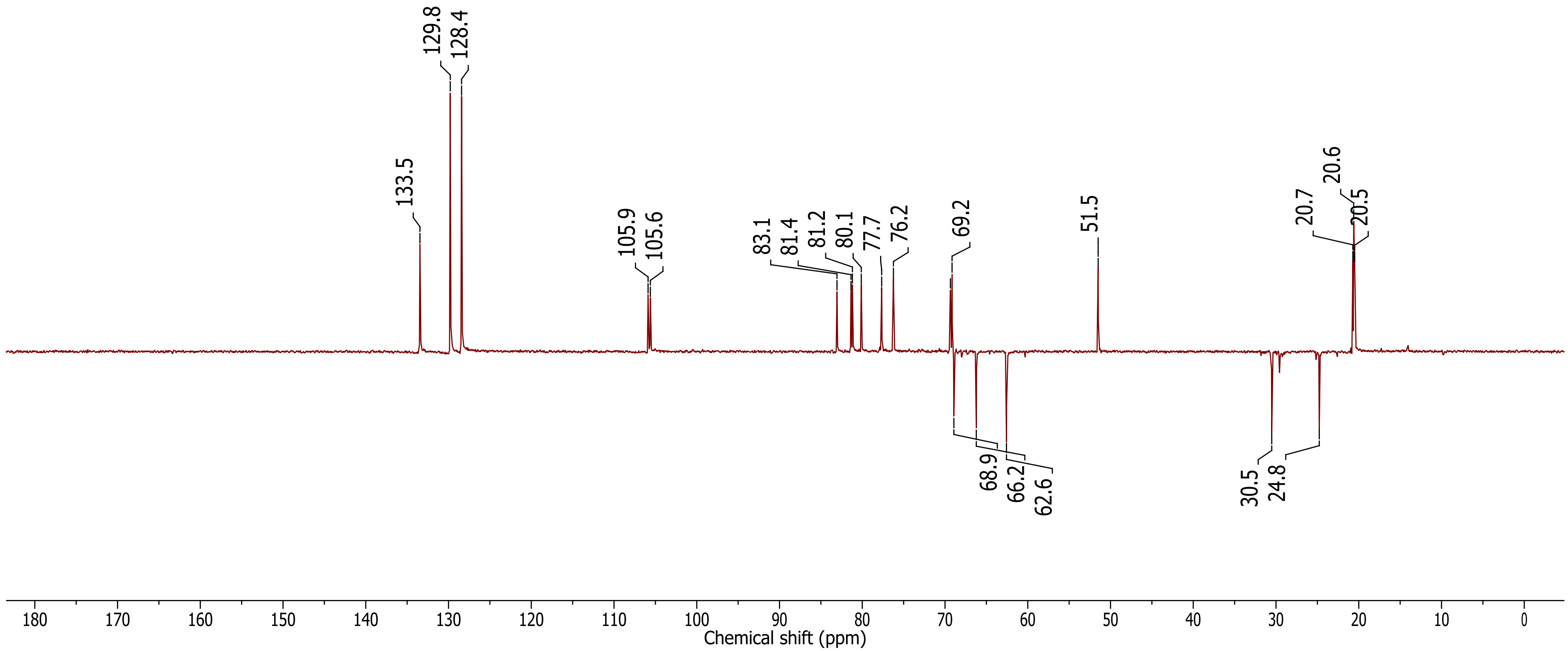

Supplementary Figure 22. ^{1}H NMR Spectrum (399.78 MHz, $CDCl_3$) Of Compound **20**

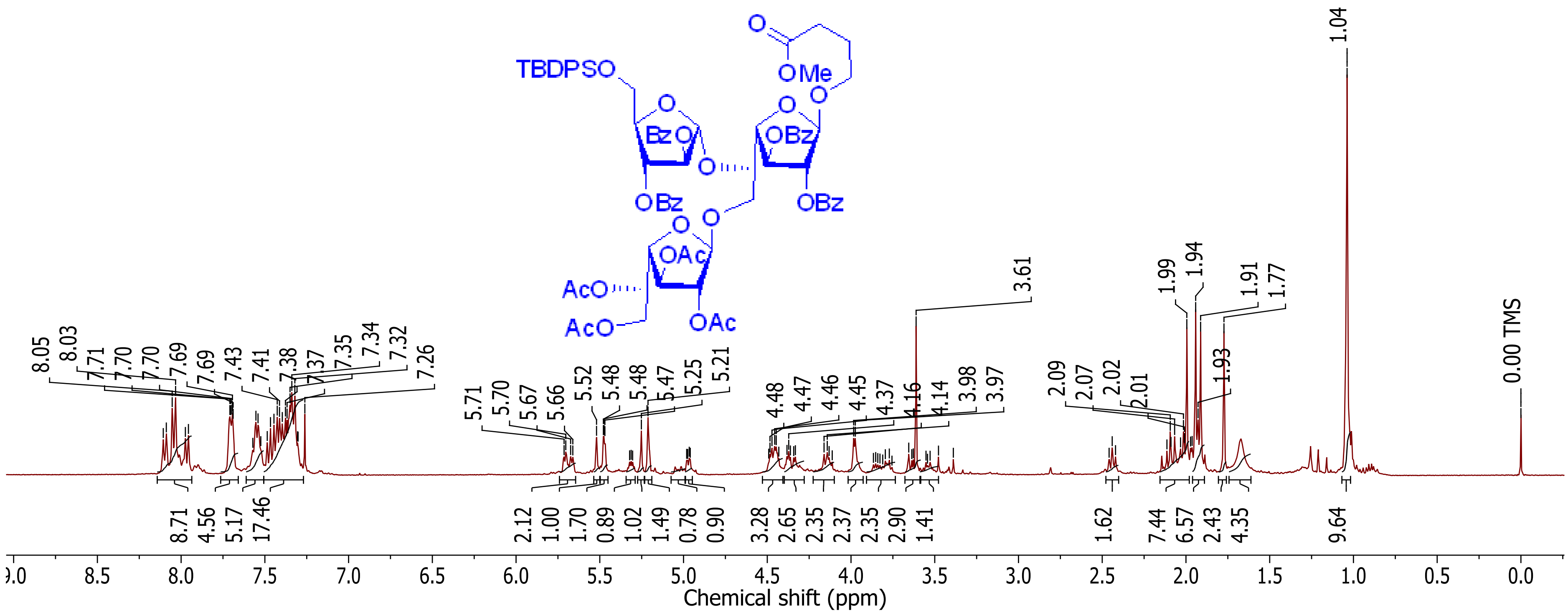

Supplementary Figure 23. ^{13}C NMR Spectrum (100.53 MHz, $CDCl_3$) Of Compound **20**

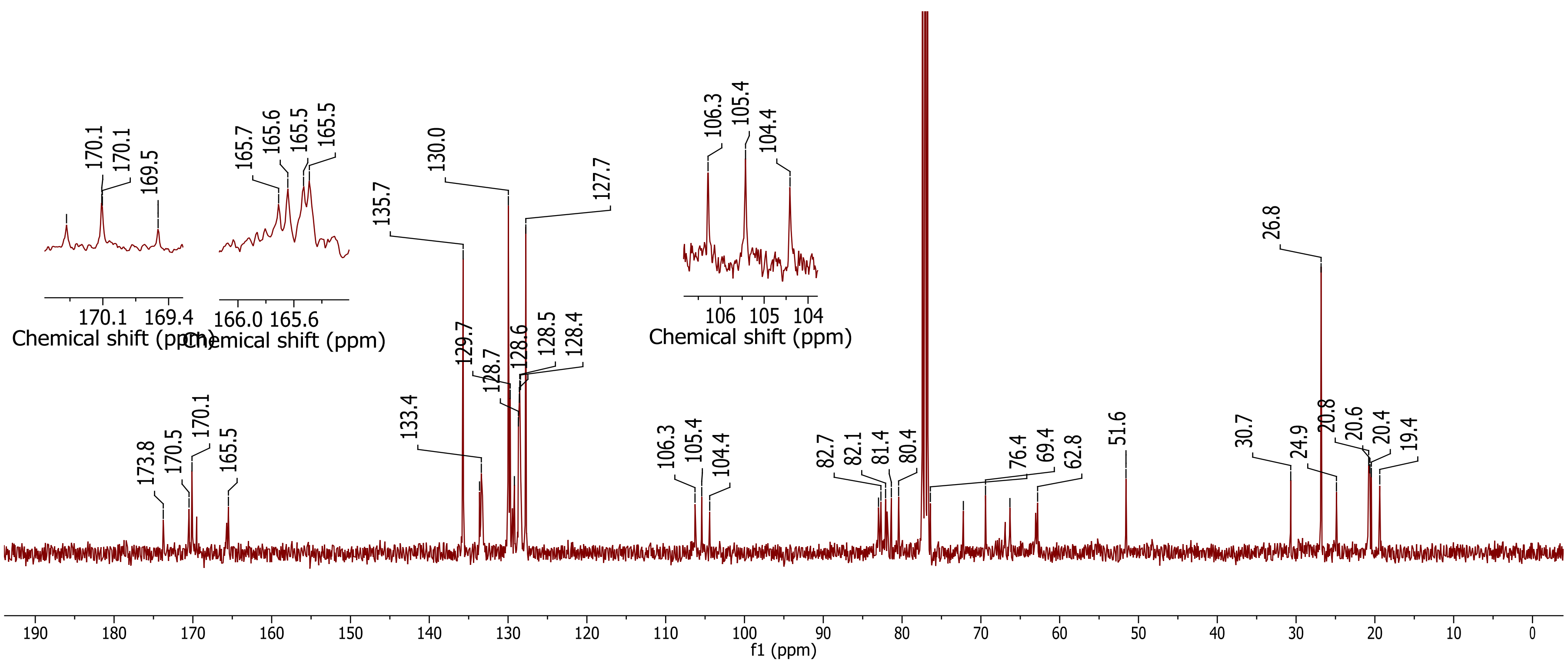

Supplementary Figure 24. DEPT NMR Spectrum (100.53 MHz, $CDCl_3$) Of Compound **20**

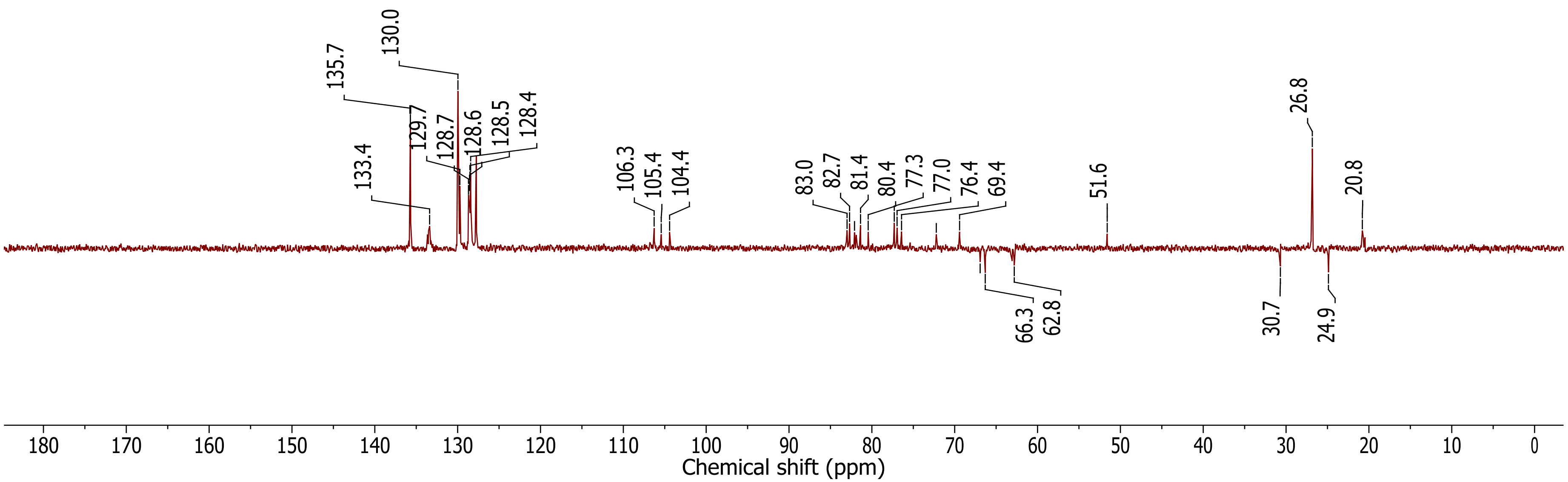

Supplementary Figure 25. ^{1}H NMR Spectrum (399.78 MHz, $CDCl_3$) of Compound **7**

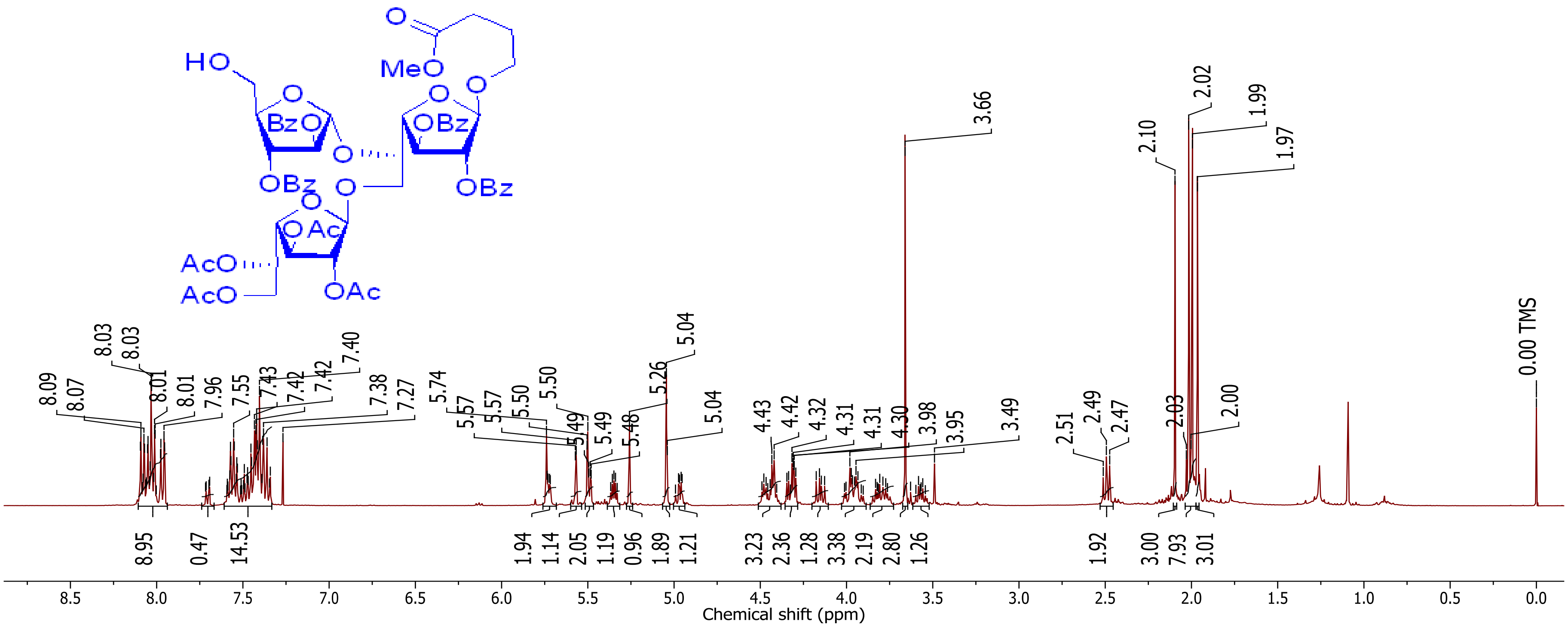

Supplementary Figure 26. ^{13}C NMR Spectrum (100.53 MHz, $CDCl_3$) of Compound **7**

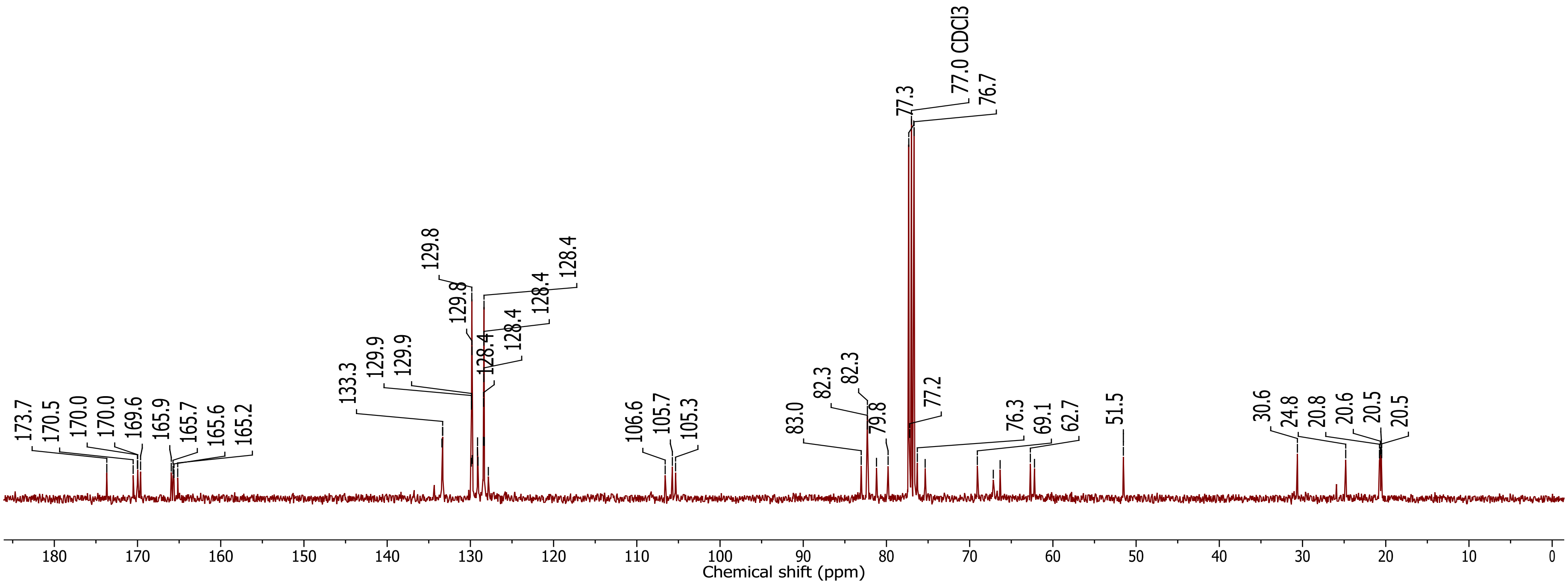

Supplementary Figure 27. DEPT NMR Spectrum (100.53 MHz, $CDCl_3$) of Compound **7**

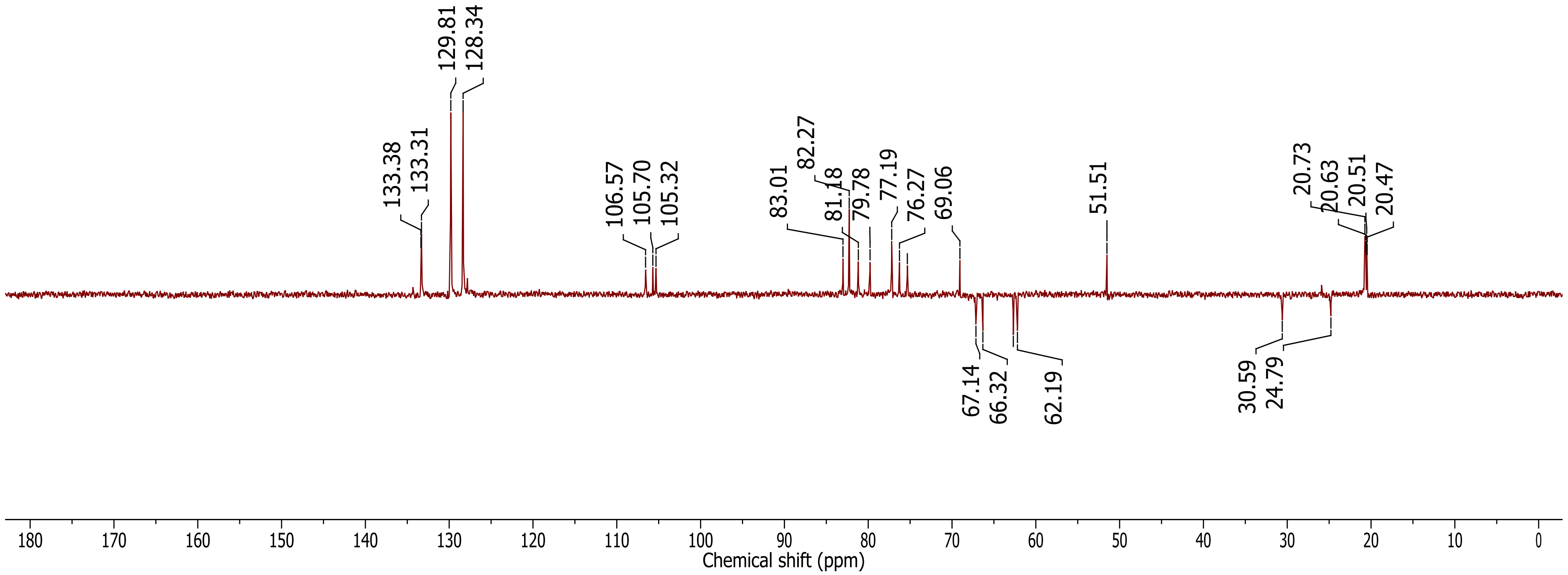

Supplementary Figure 28. ^{1}H NMR Spectrum (399.78 MHz, $CDCl_3$) of Compound **21**

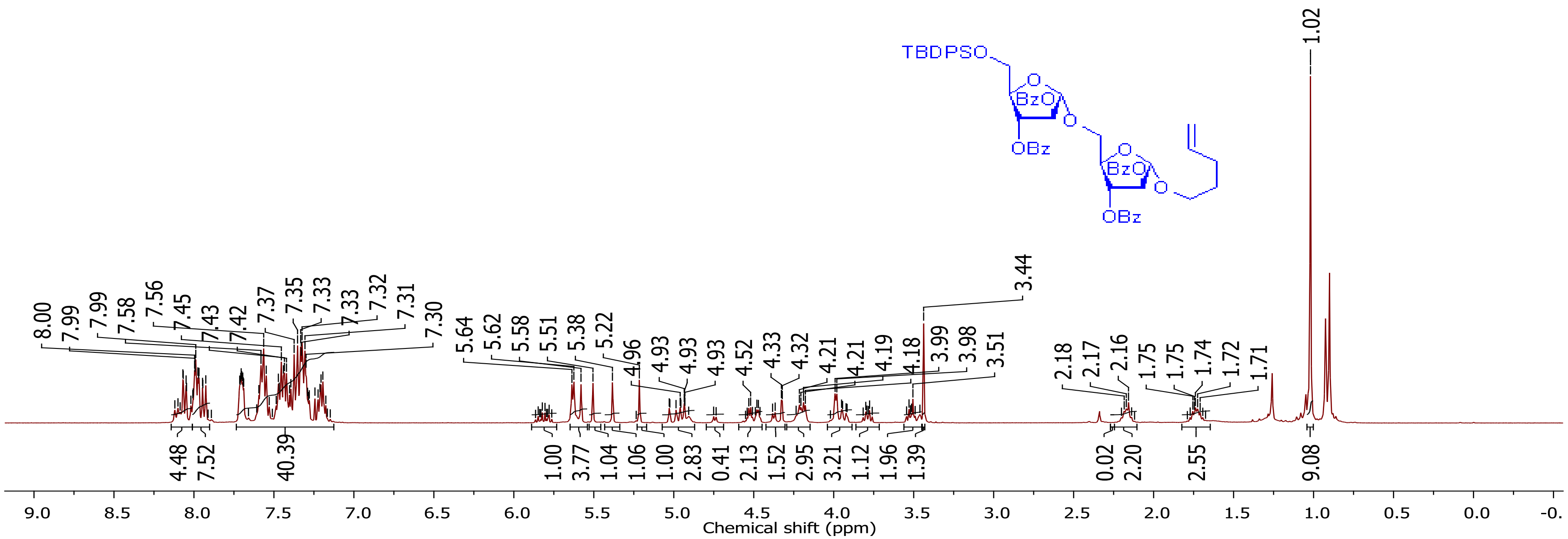

Supplementary Figure 29. ^{13}C NMR Spectrum (100.53 MHz, $CDCl_3$) of Compound **21**

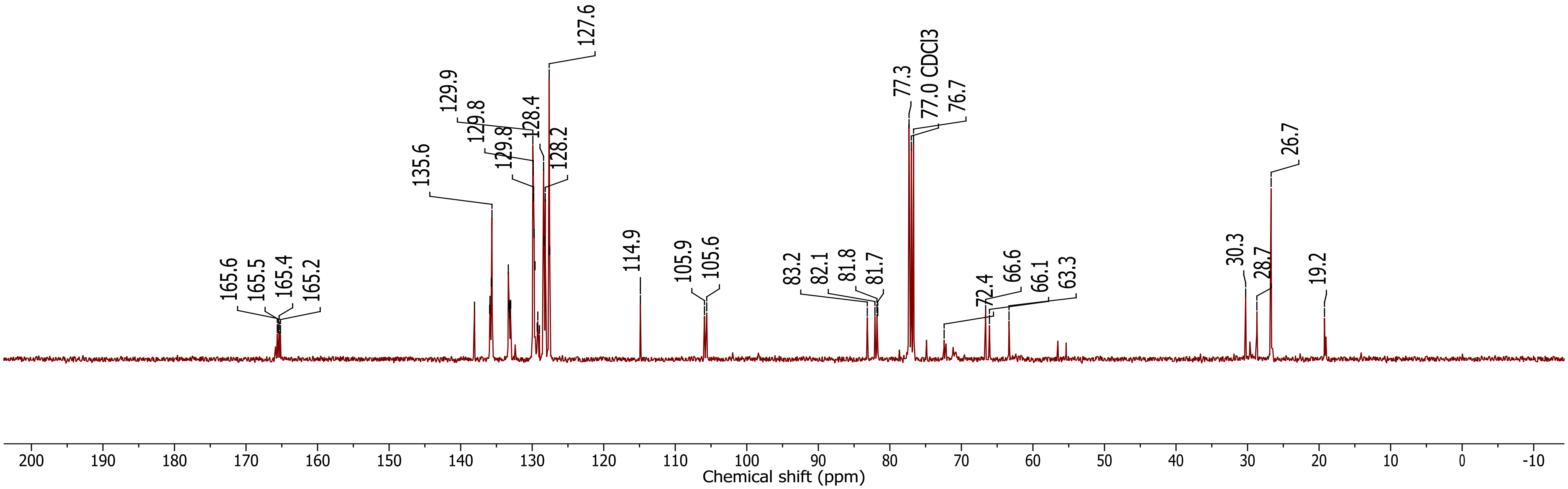

Supplementary Figure 30. DEPT NMR Spectrum (100.53 MHz, $CDCl_3$) of Compound **21**

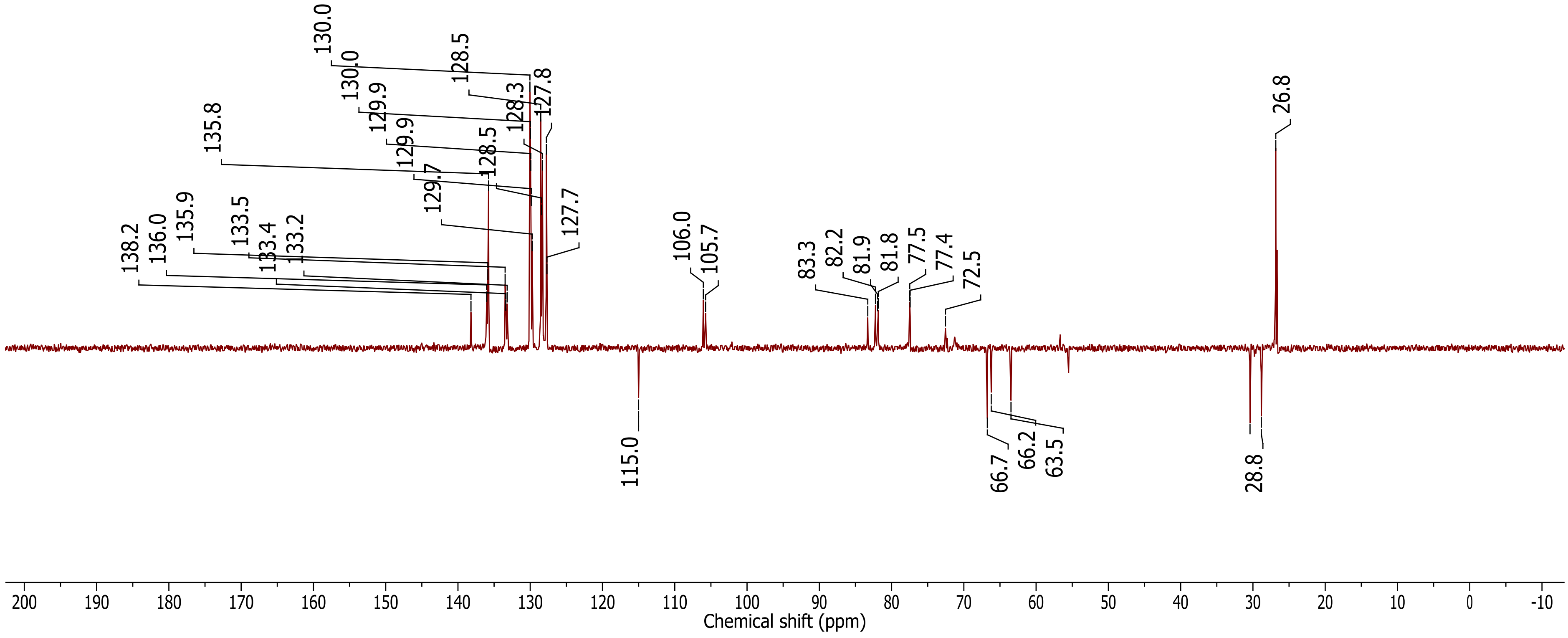

Supplementary Figure 31. ^{1}H NMR Spectrum (399.78 MHz, $CDCl_3$) of Compound **23**

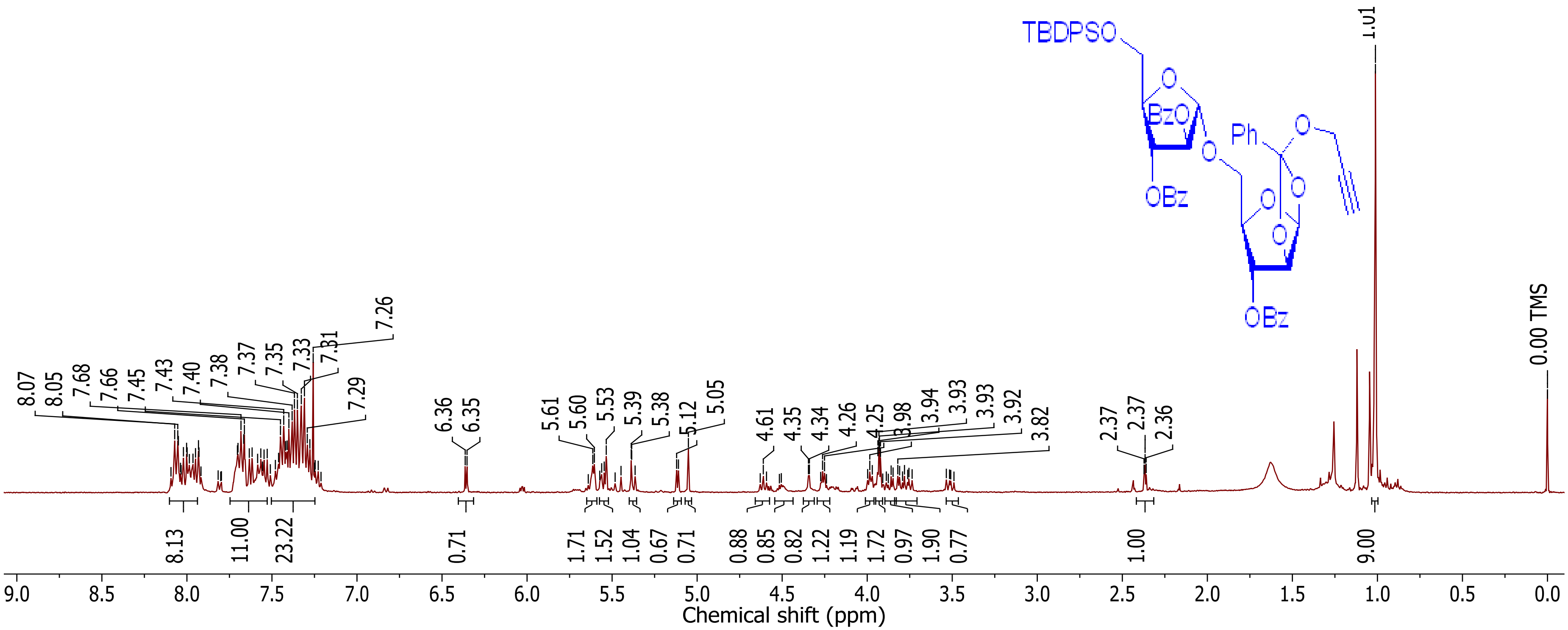

Supplementary Figure 32. ^{13}C NMR Spectrum (100.53 MHz, $CDCl_3$) of Compound **23**

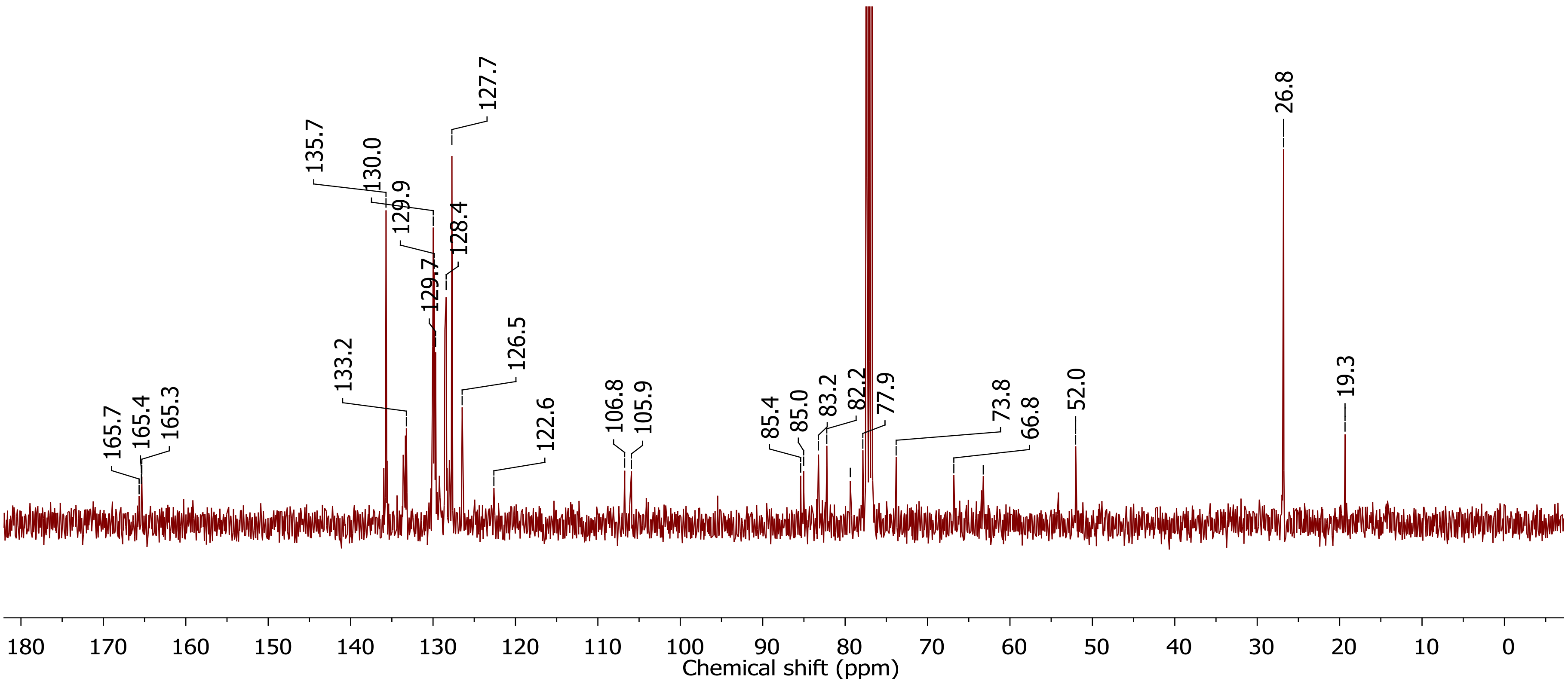

Supplementary Figure 33. DEPT NMR Spectrum (100.53 MHz, $CDCl_3$) of Compound **23**

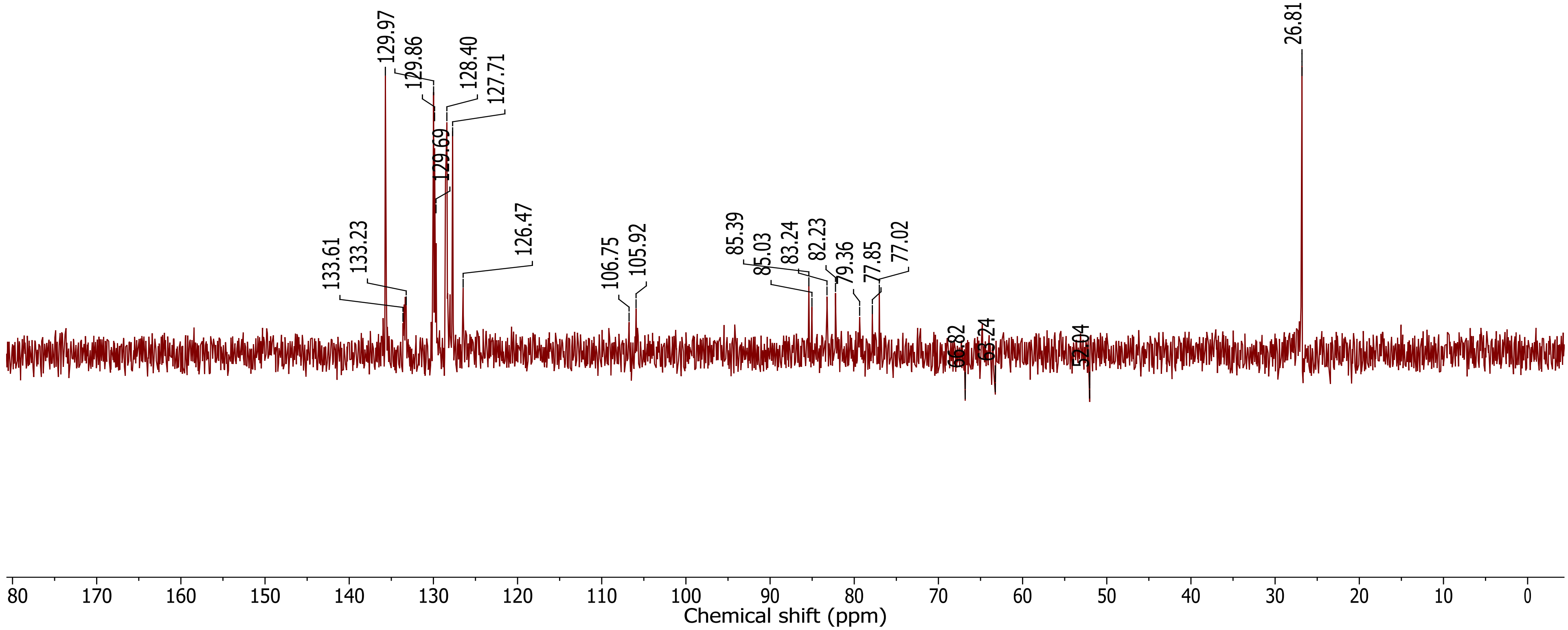

Supplementary Figure 34. ^{1}H NMR Spectrum (399.78 MHz, $CDCl_3$) of Compound **25**

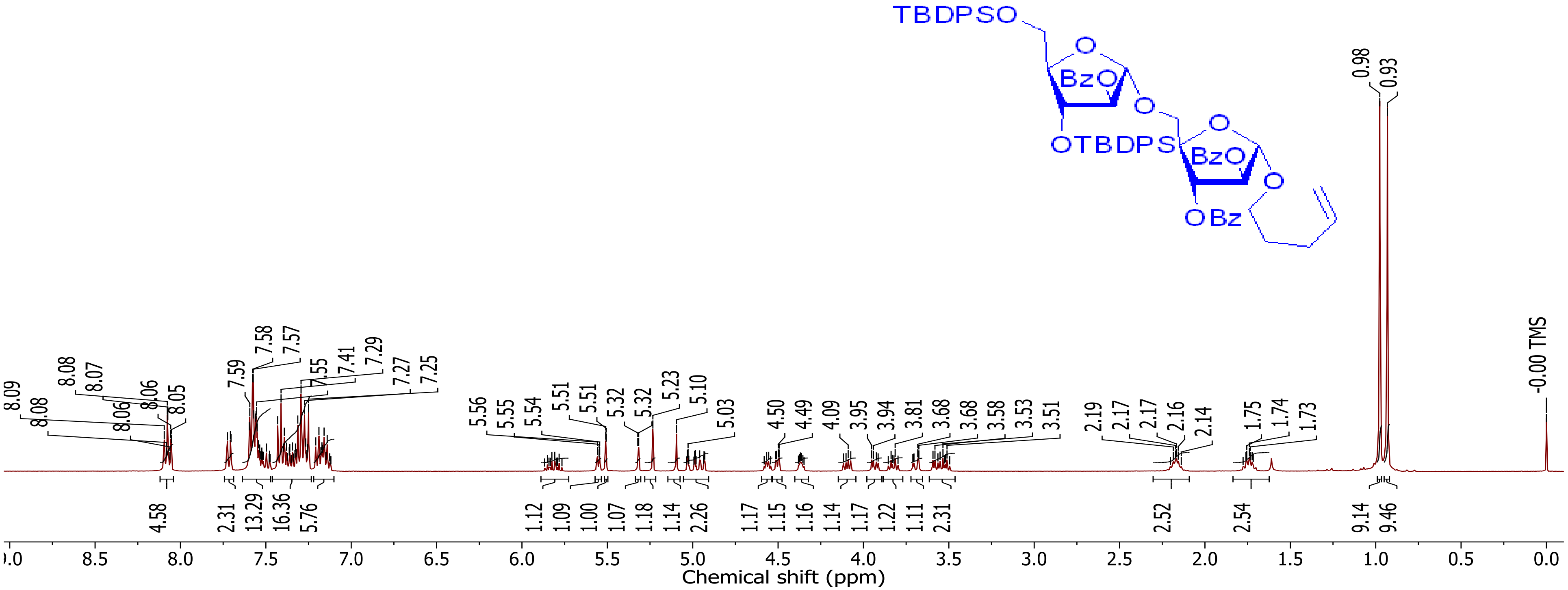

Supplementary Figure 35. ^{13}C NMR Spectrum (100.53 MHz, $CDCl_3$) of Compound **25**

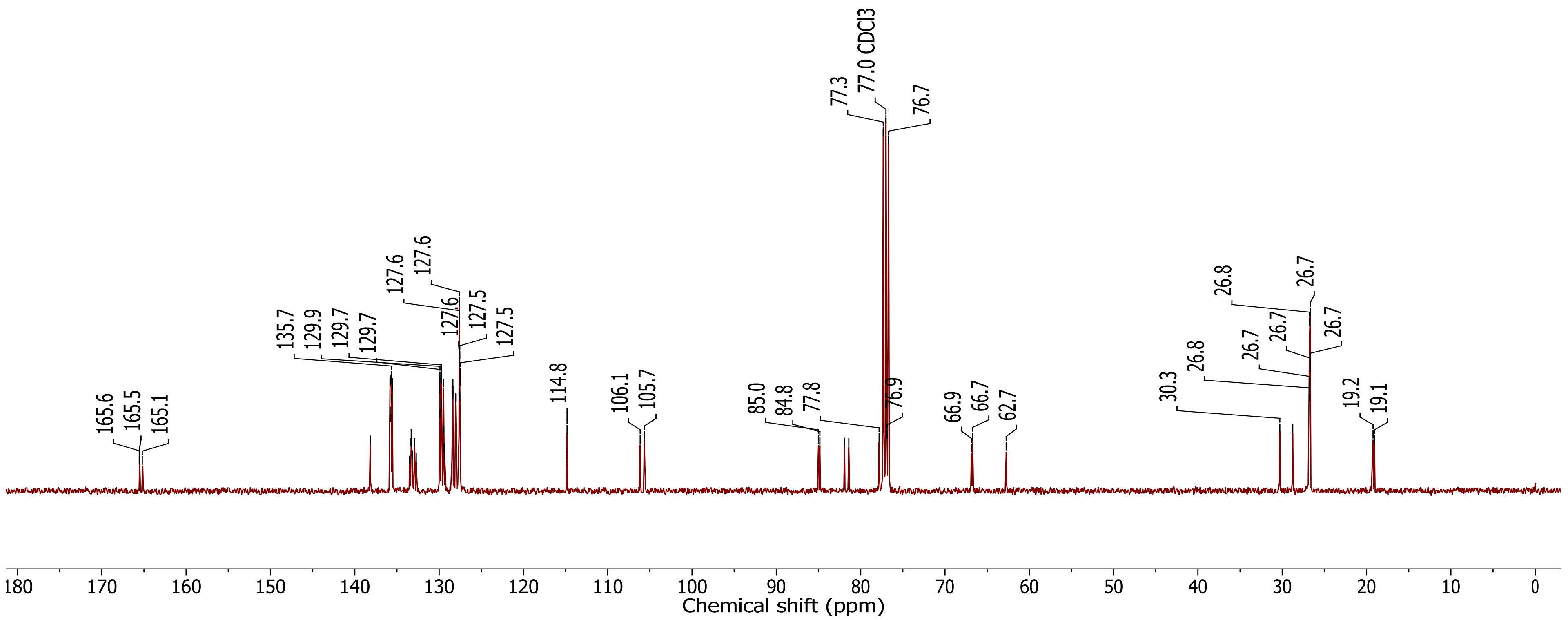

Supplementary Figure 36. DEPT NMR Spectrum (100.53 MHz, $CDCl_3$) of Compound **25**

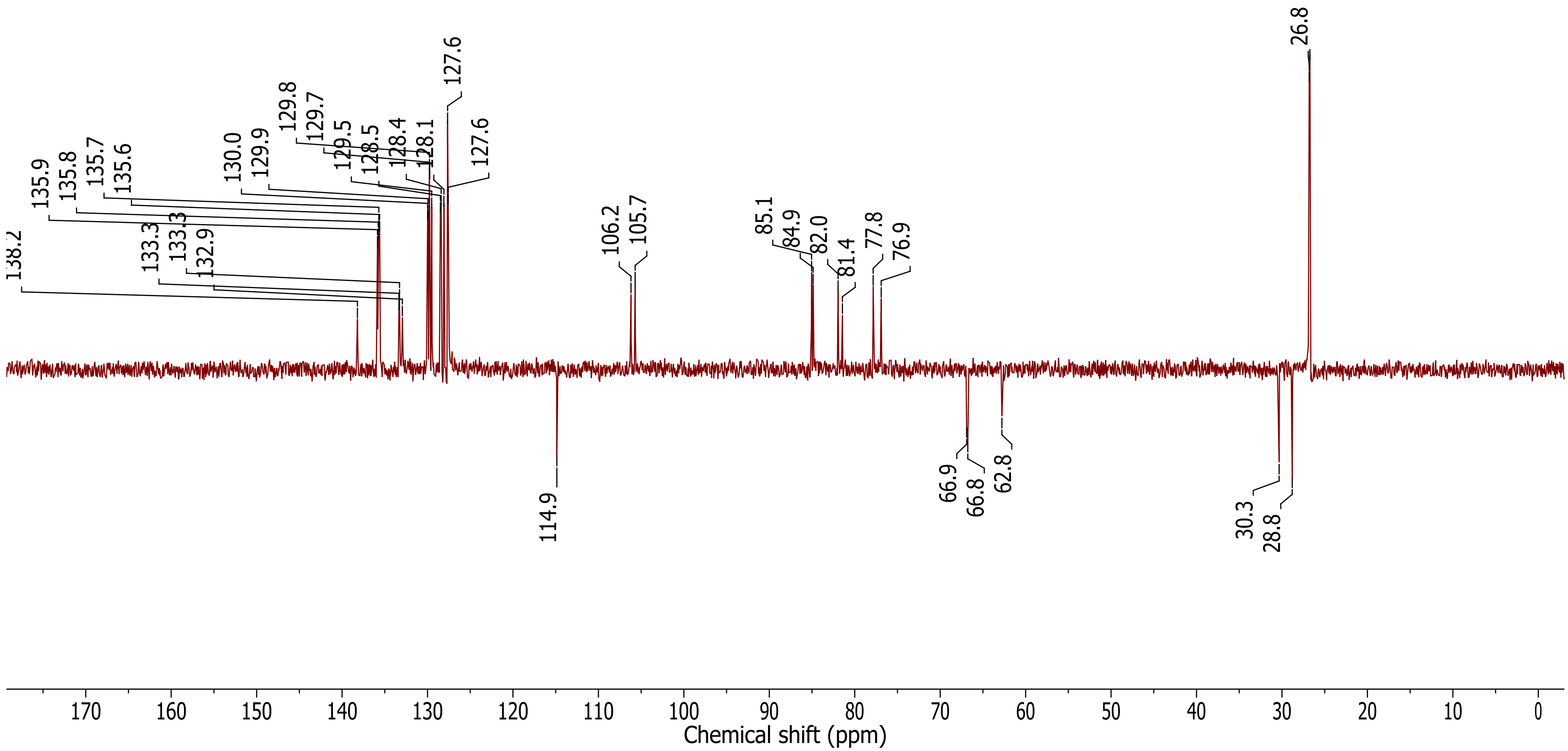

Supplementary Figure 37. ^{1}H NMR Spectrum (399.78 MHz, $CDCl_3$) of Compound **26**

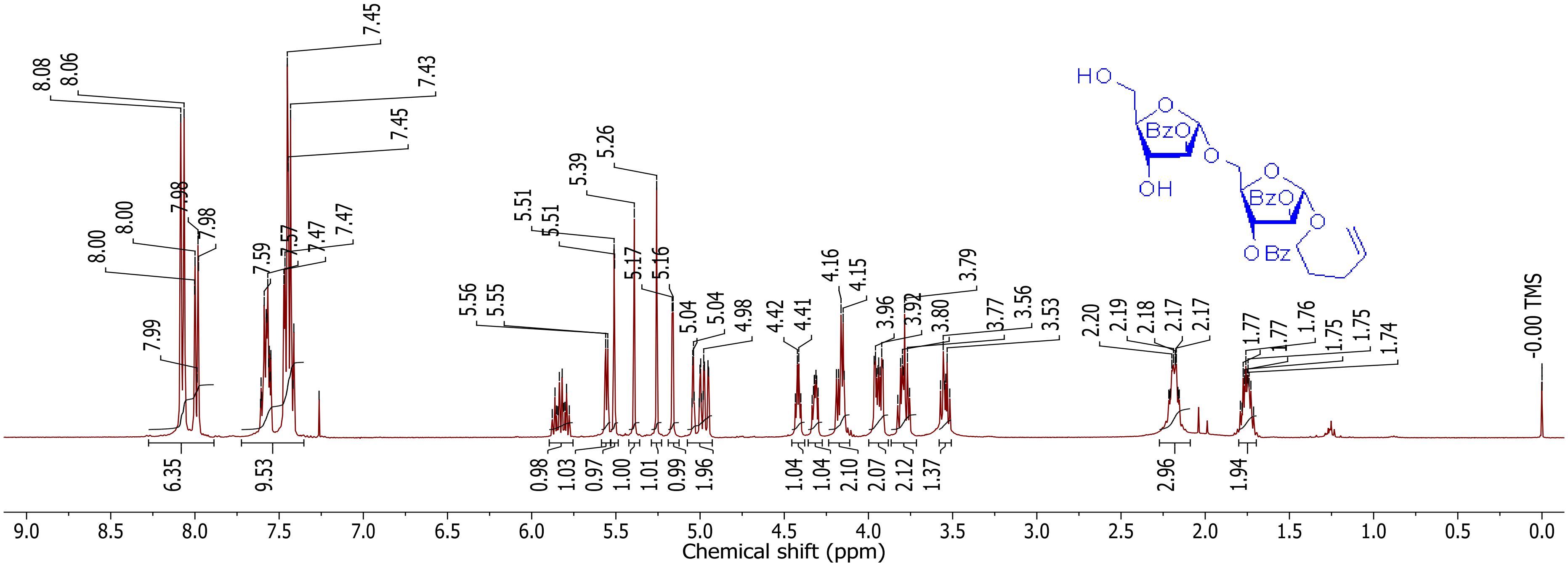

Supplementary Figure 38. ^{13}C NMR Spectrum (100.53 MHz, $CDCl_3$) of Compound **26**

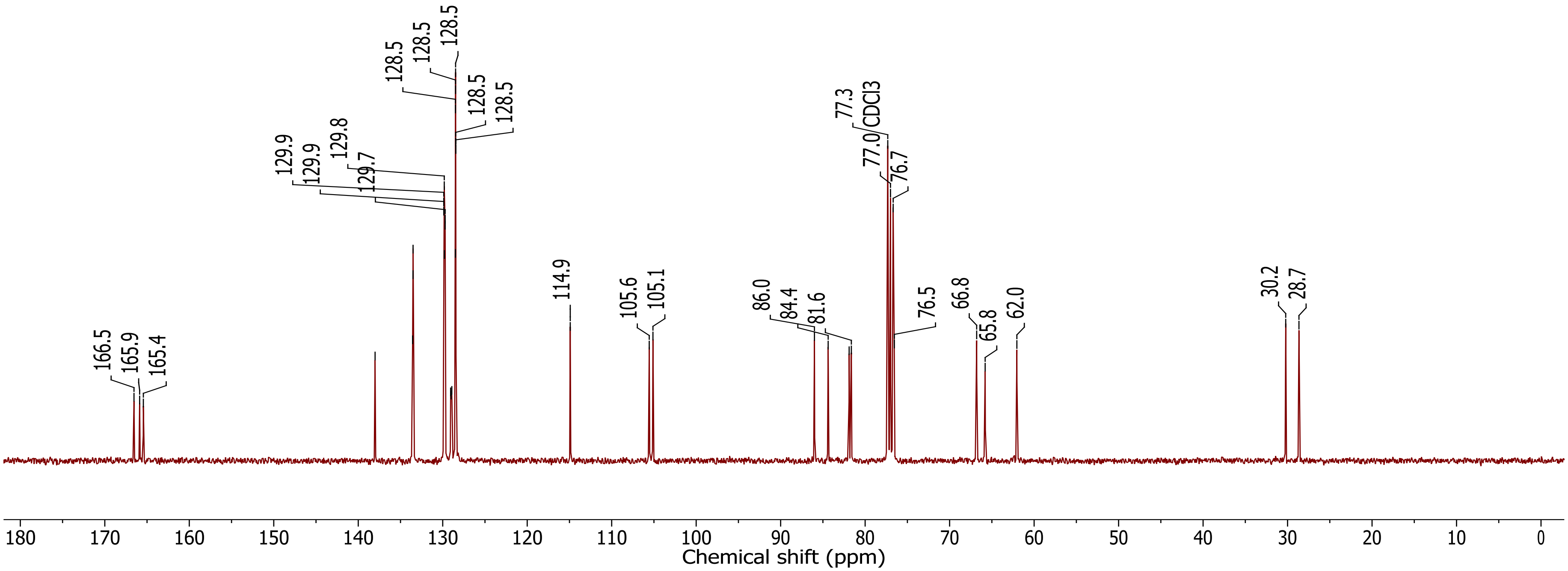

Supplementary Figure 39. DEPT NMR Spectrum (100.53 MHz, $CDCl_3$) of Compound **26**

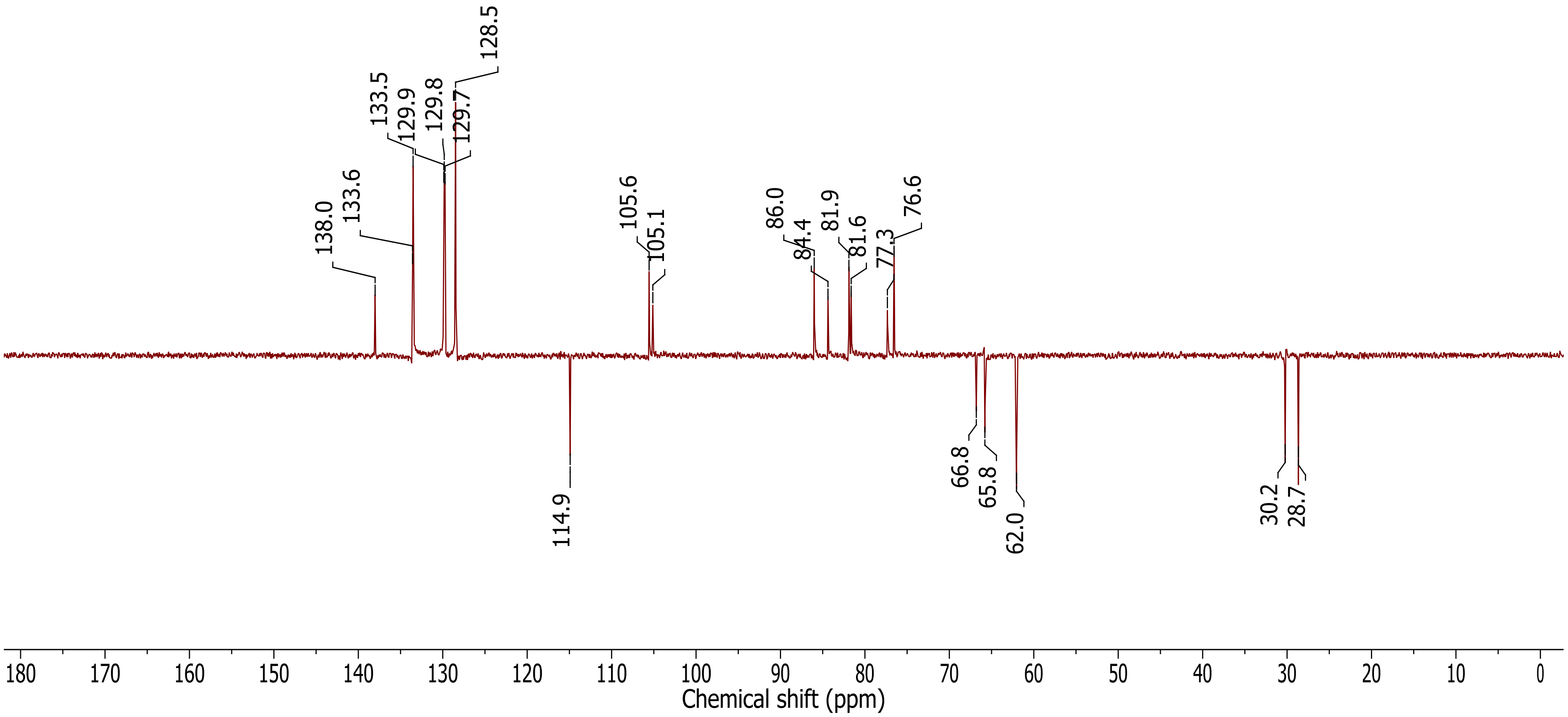

Supplementary Figure 40. ^{1}H NMR Spectrum (399.78 MHz, $CDCl_3$) of a Compound **27**

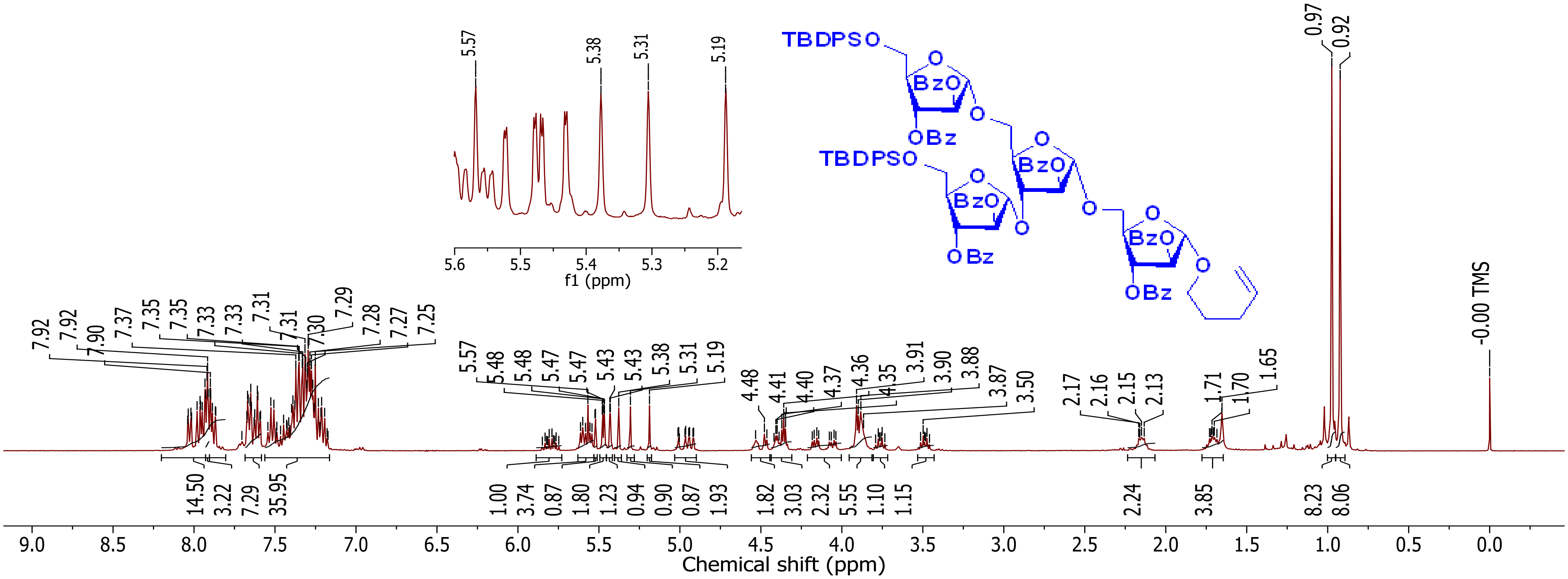

Supplementary Figure 41. ^{13}C NMR Spectrum (100.53 MHz, $CDCl_3$) of Compound to **27**

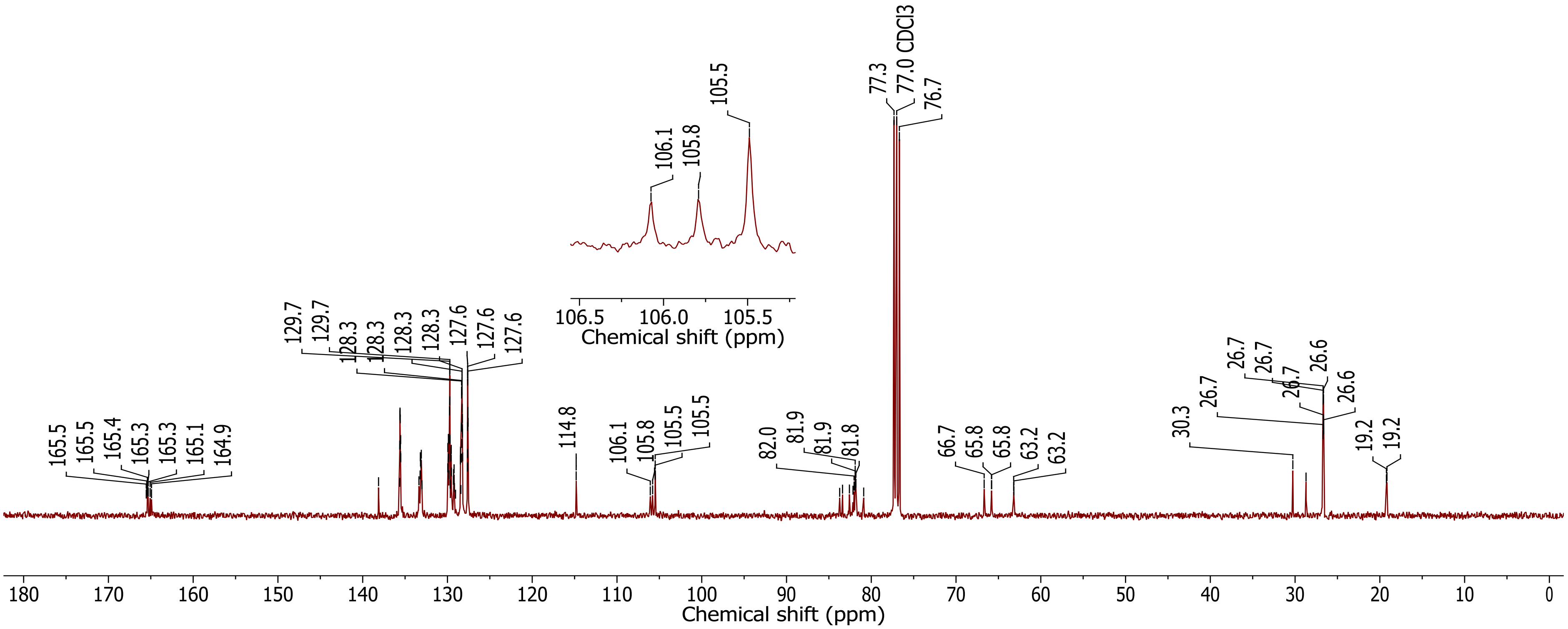

Supplementary Figure 42. DEPT NMR Spectrum (100.53 MHz, $CDCl_3$) of Compound to **27**

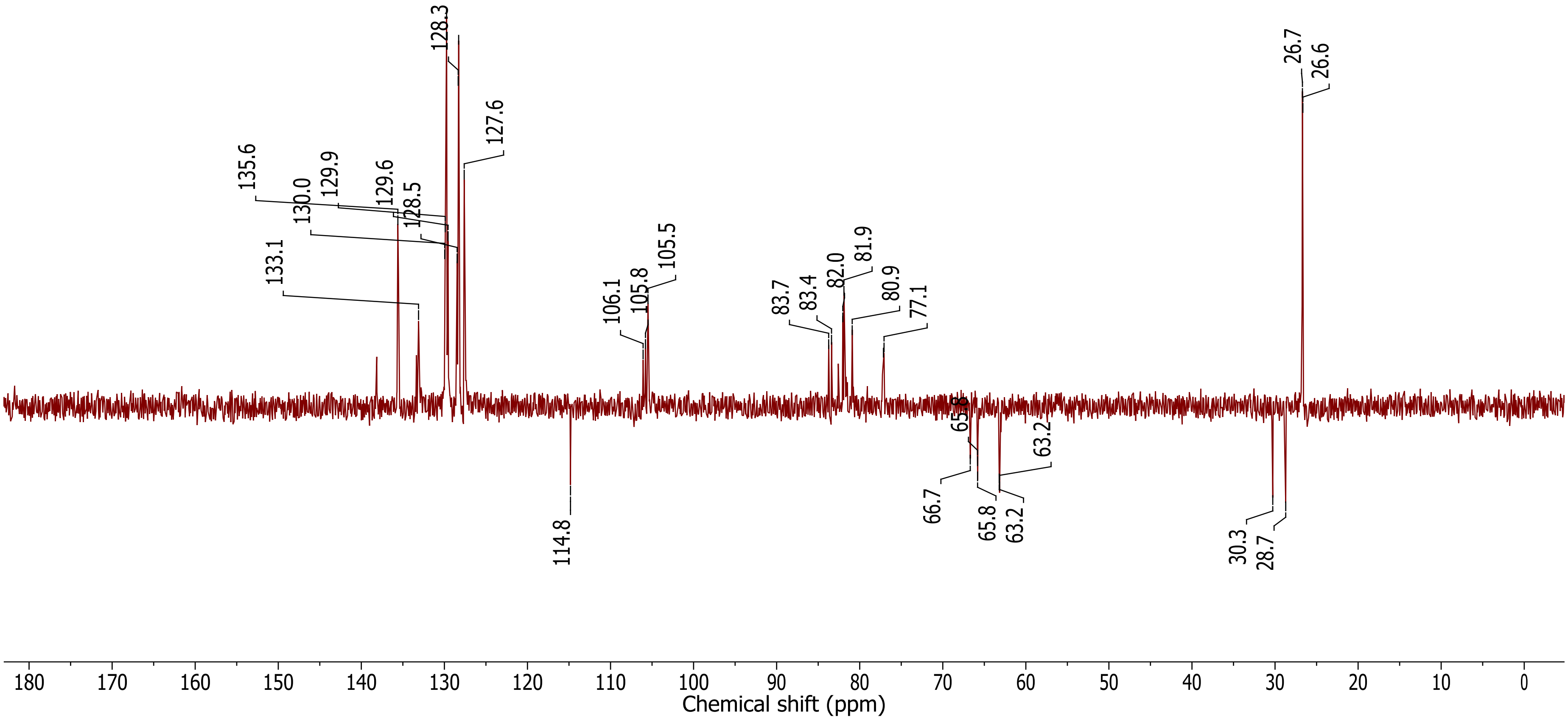

Supplementary Figure 43. ^{1}H NMR Spectrum (399.78 MHz, $CDCl_3$) of Compound *En route* to **28**

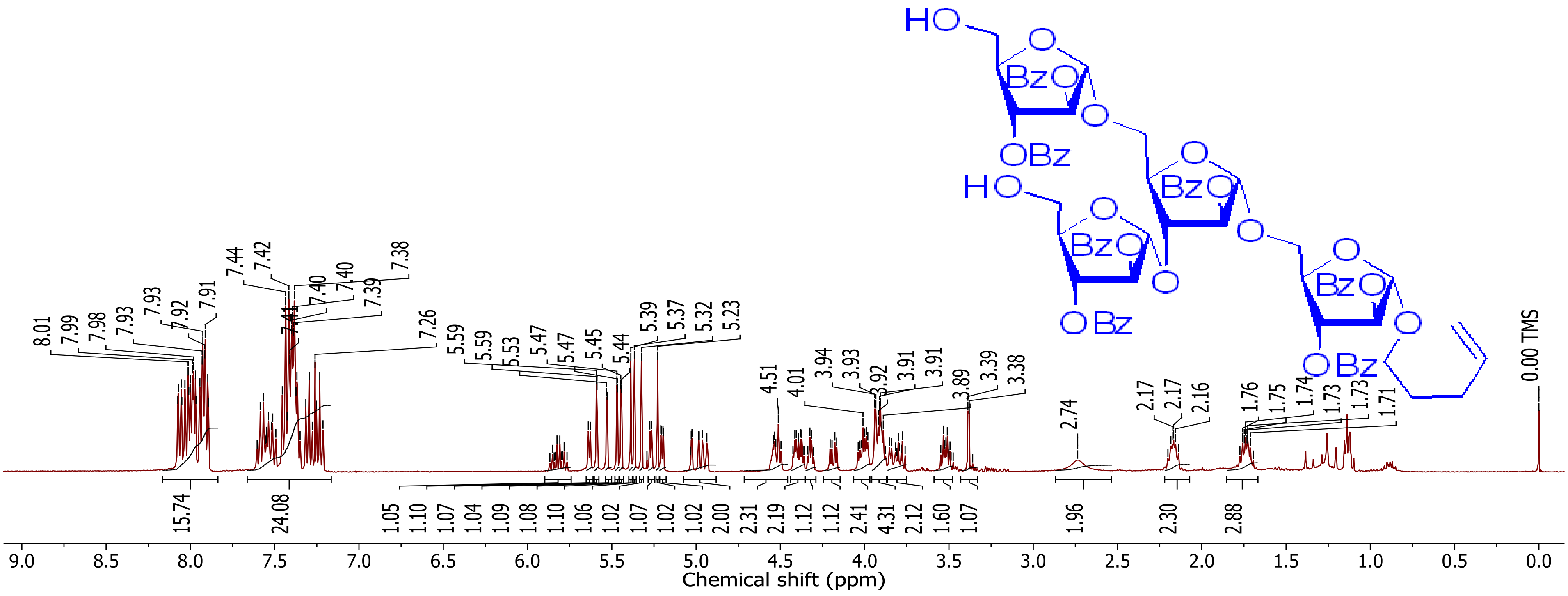

Supplementary Figure 44. ^{13}C NMR Spectrum (100.53 MHz, $CDCl_3$) of Compound *En route* to **28**

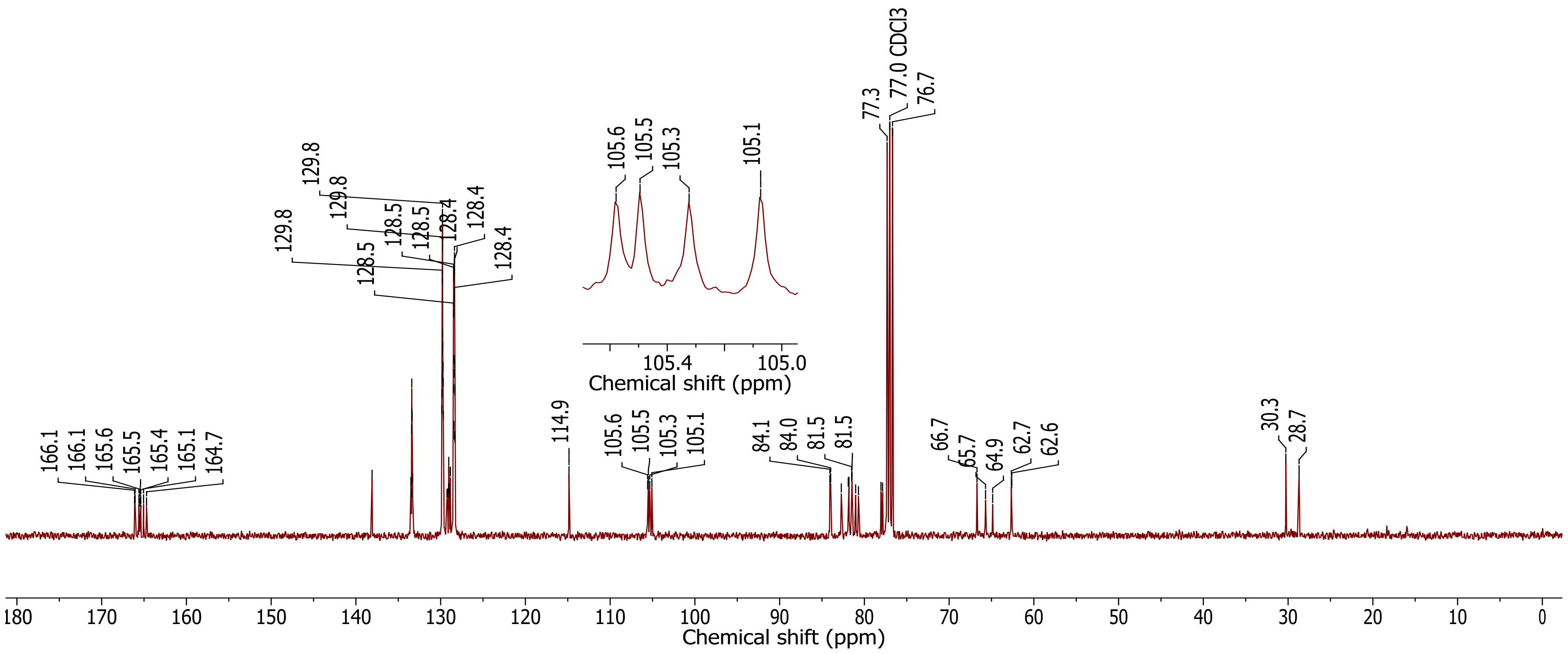

Supplementary Figure 45. DEPT NMR Spectrum (100.53 MHz, $CDCl_3$) of Compound *En route* to **28**

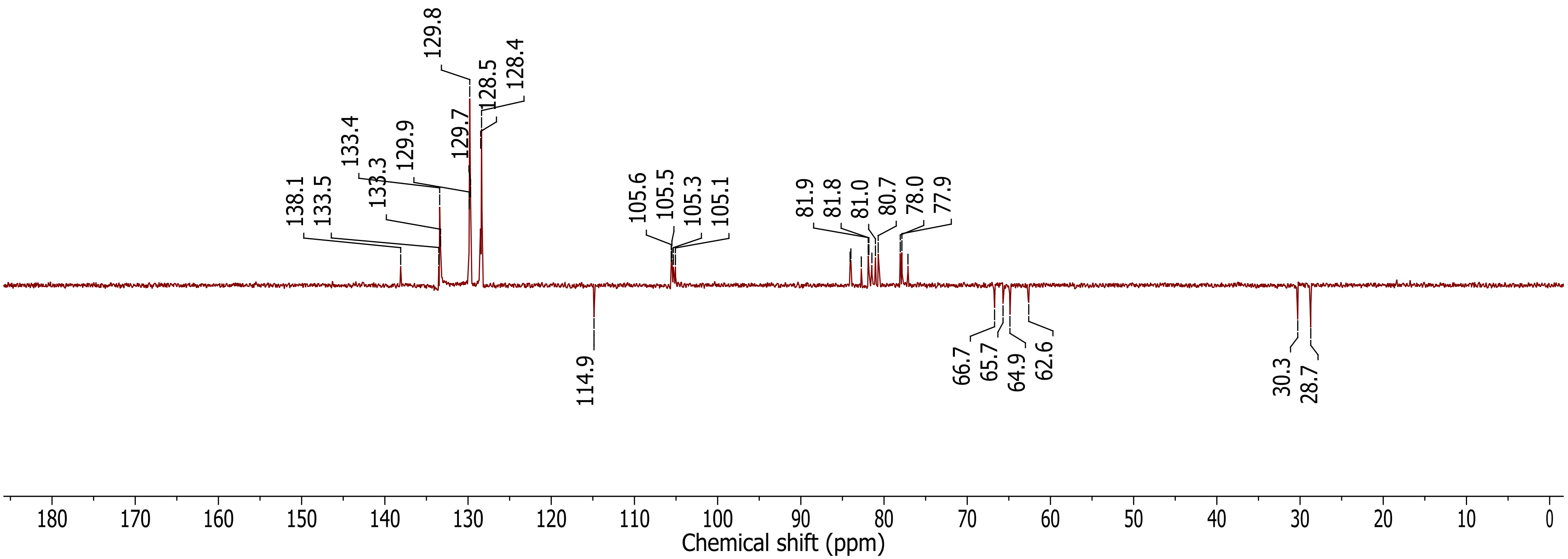

Supplementary Figure 46. ^{1}H NMR Spectrum (399.78 MHz, $CDCl_3$) of Compound **28**

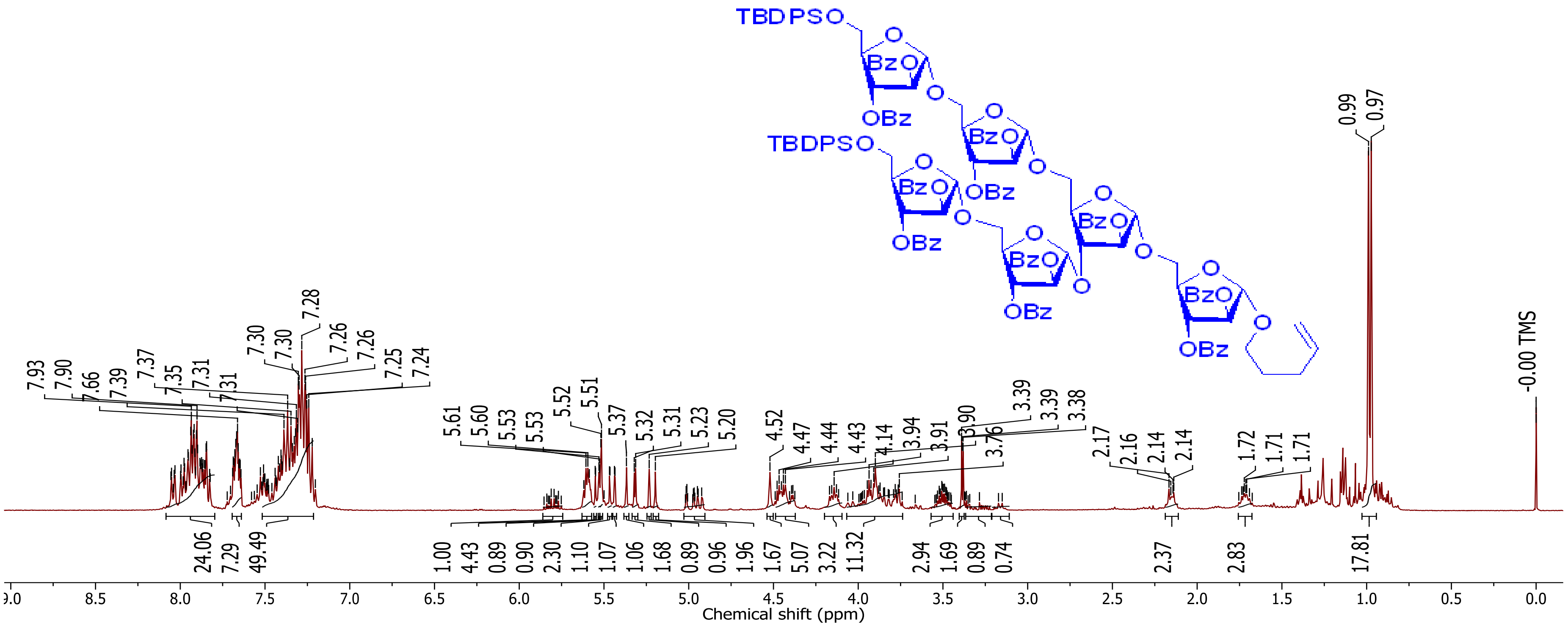

Supplementary Figure 47. ^{13}C NMR Spectrum (100.53 MHz, $CDCl_3$) of Compound **28**

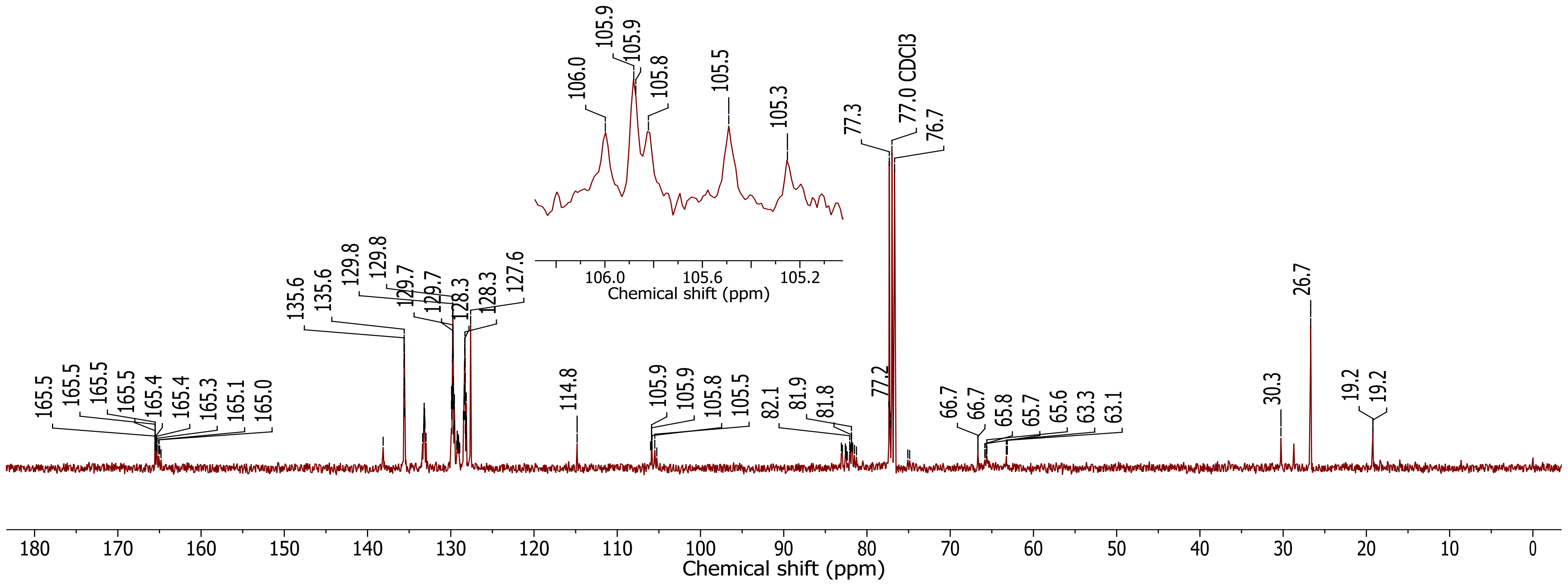

Supplementary Figure 48. DEPT NMR Spectrum (100.53 MHz, $CDCl_3$) of Compound **28**

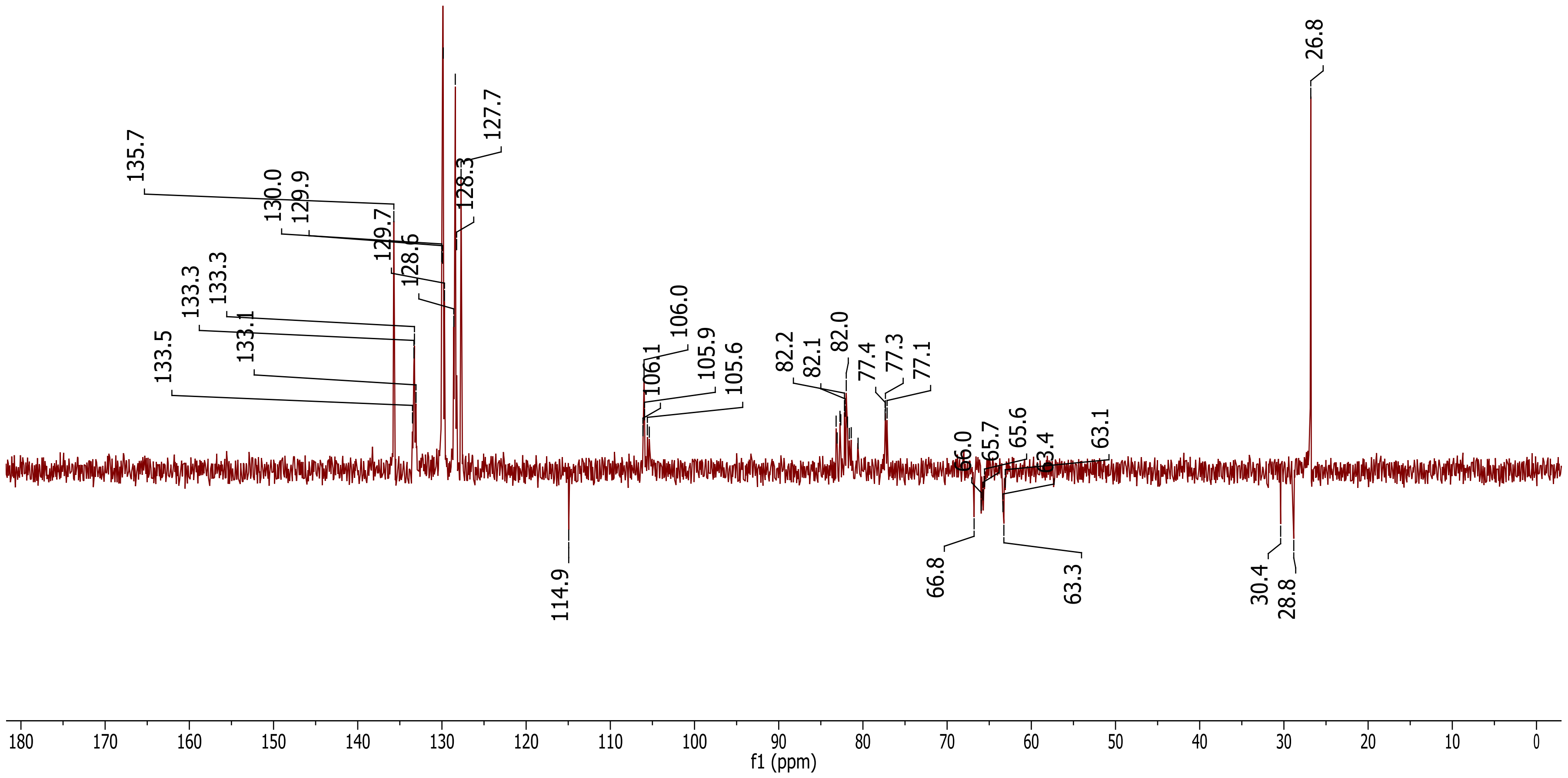

Supplementary Figure 49. ^{1}H NMR Spectrum (399.78 MHz, $CDCl_3$) of Compound **4**

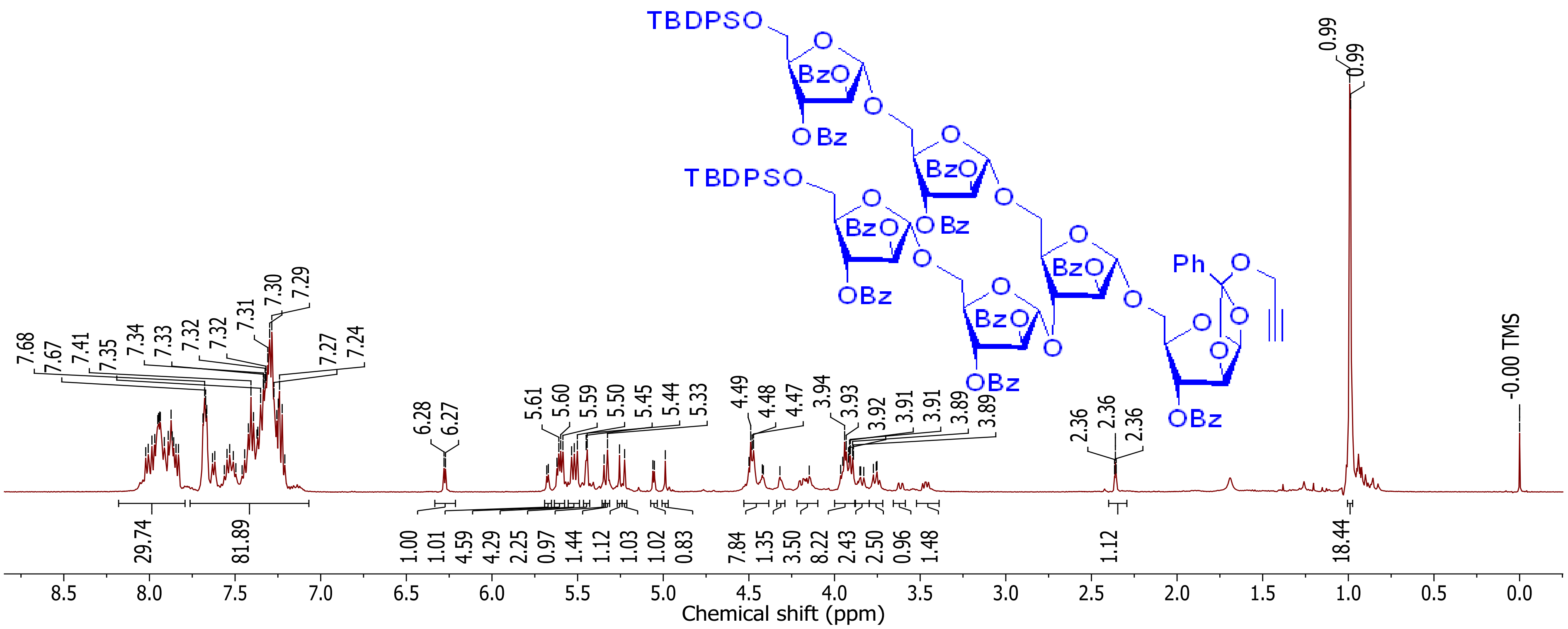

Supplementary Figure 50. ^{13}C NMR Spectrum (100.53 MHz, $CDCl_3$) of Compound **4**

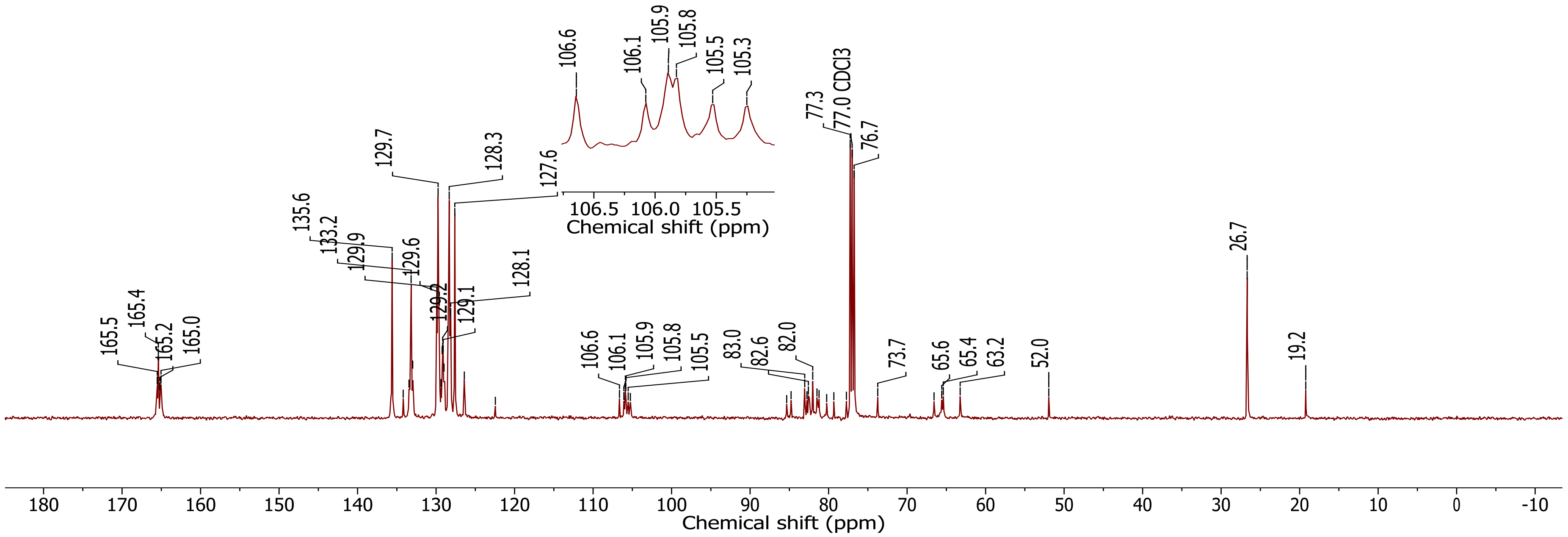

Supplementary Figure 51. DEPT NMR Spectrum (100.53 MHz, $CDCl_3$) of Compound **4**

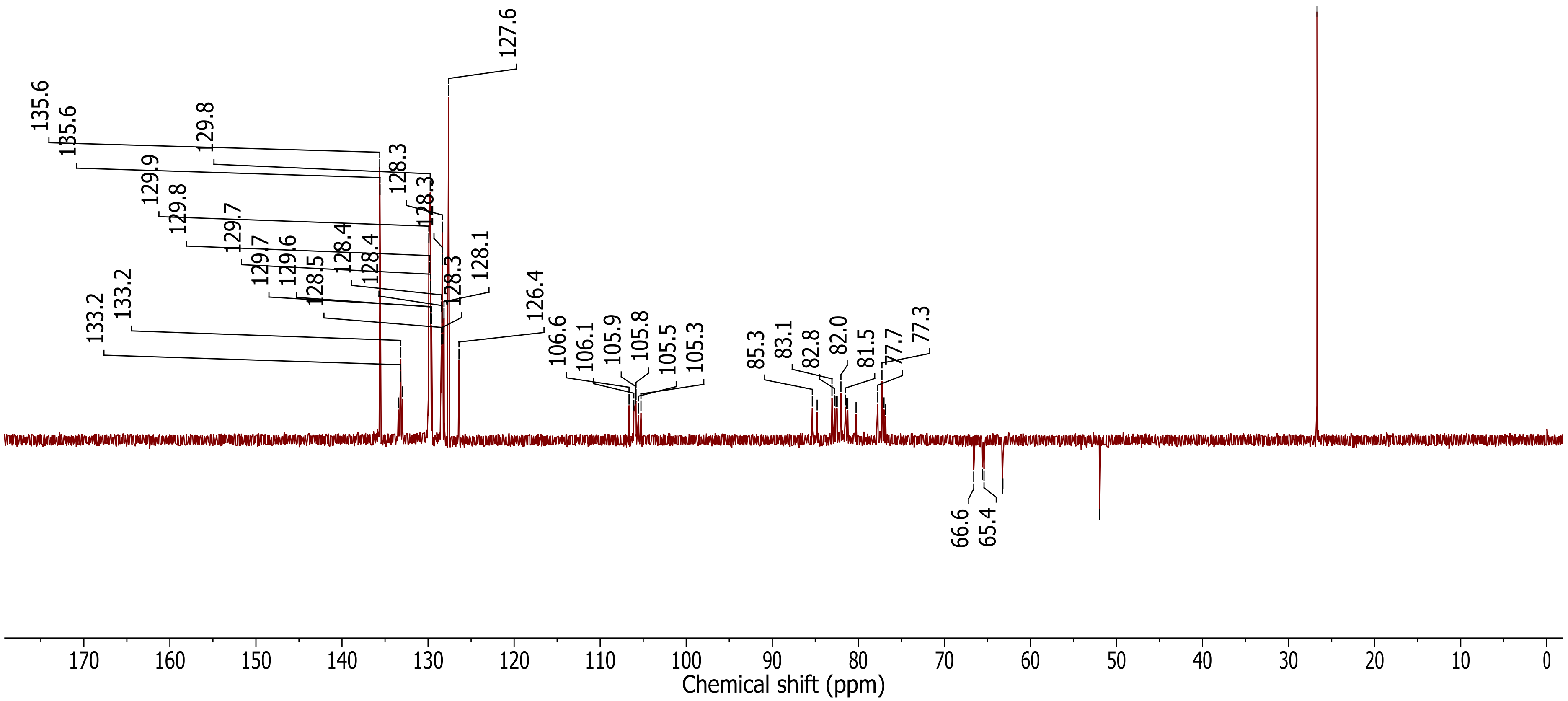

Supplementary Figure 52. ^{1}H NMR Spectrum (400.13 MHz, $CDCl_3$) of Compound **29**

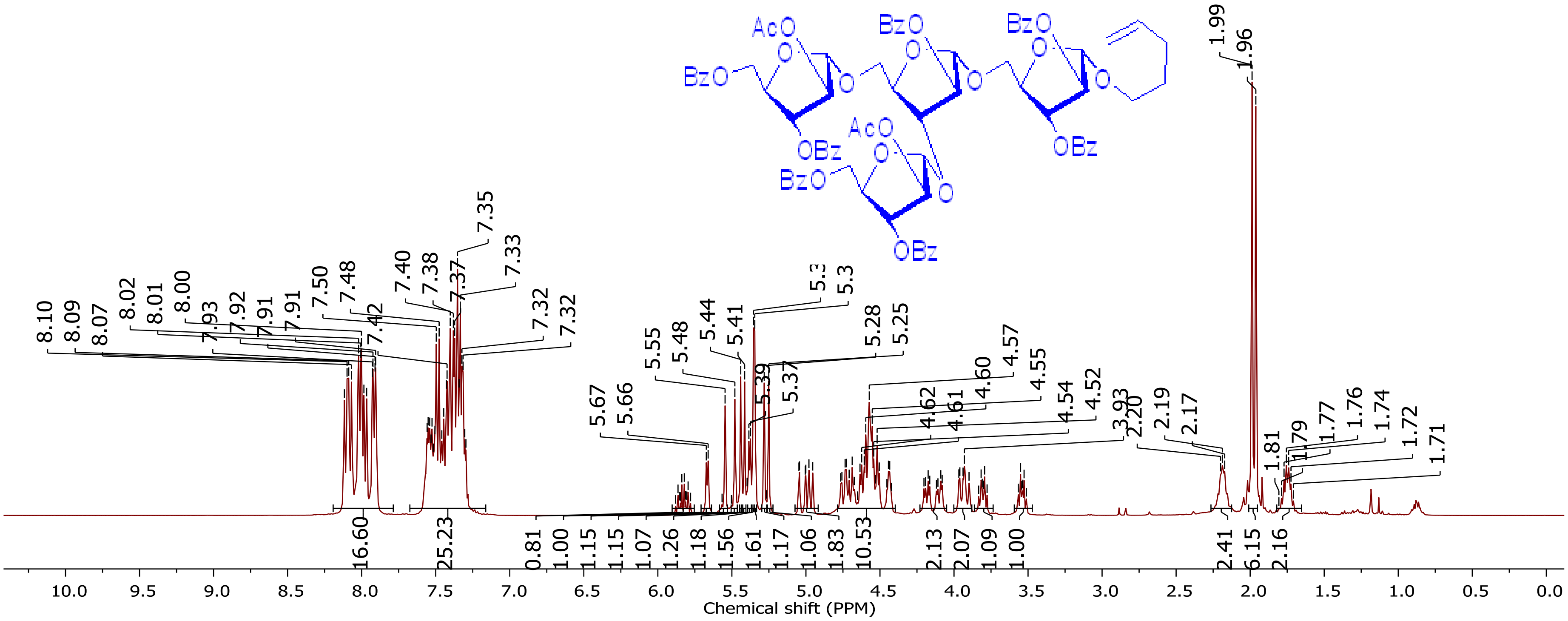

Supplementary Figure 53. ^{13}C NMR Spectrum (100.62 MHz, $CDCl_3$) of Compound **29**

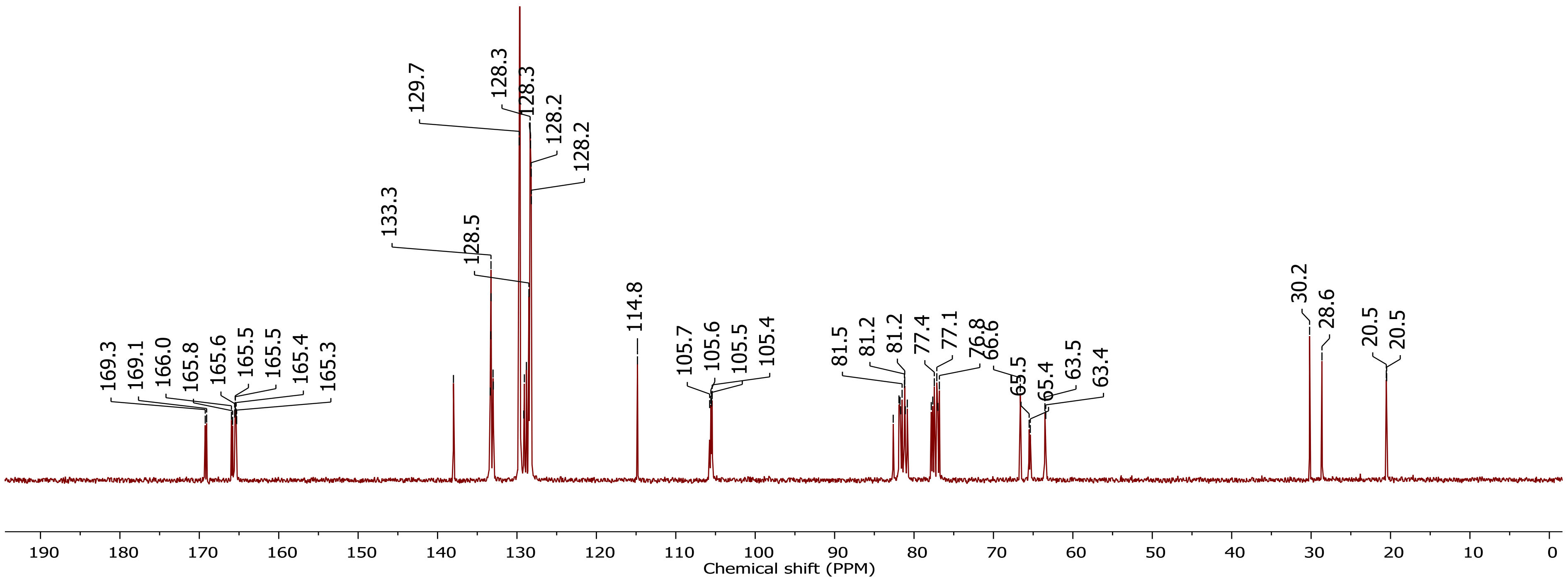

Supplementary Figure 54. DEPT NMR Spectrum (100.62 MHz, $CDCl_3$) of Compound **29**

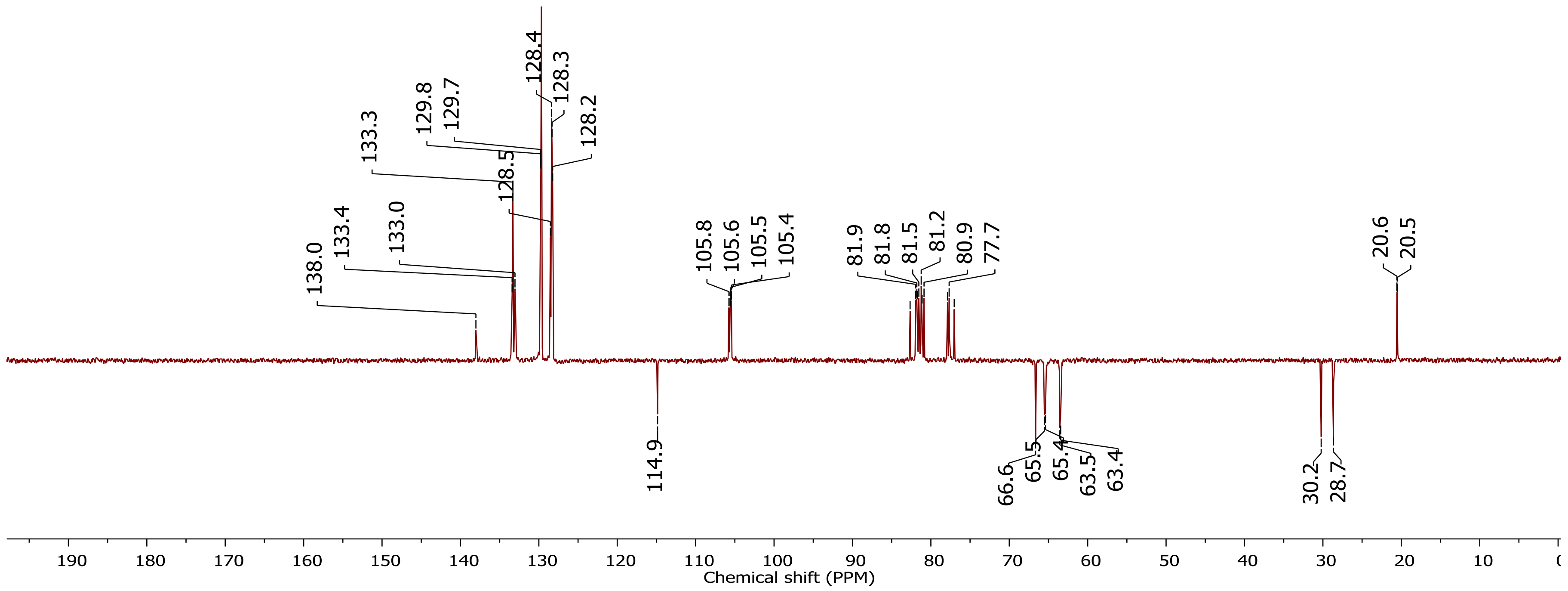

Supplementary Figure 55. ^{1}H NMR Spectrum (399.78 MHz, $CDCl_3$) of Compound **2**

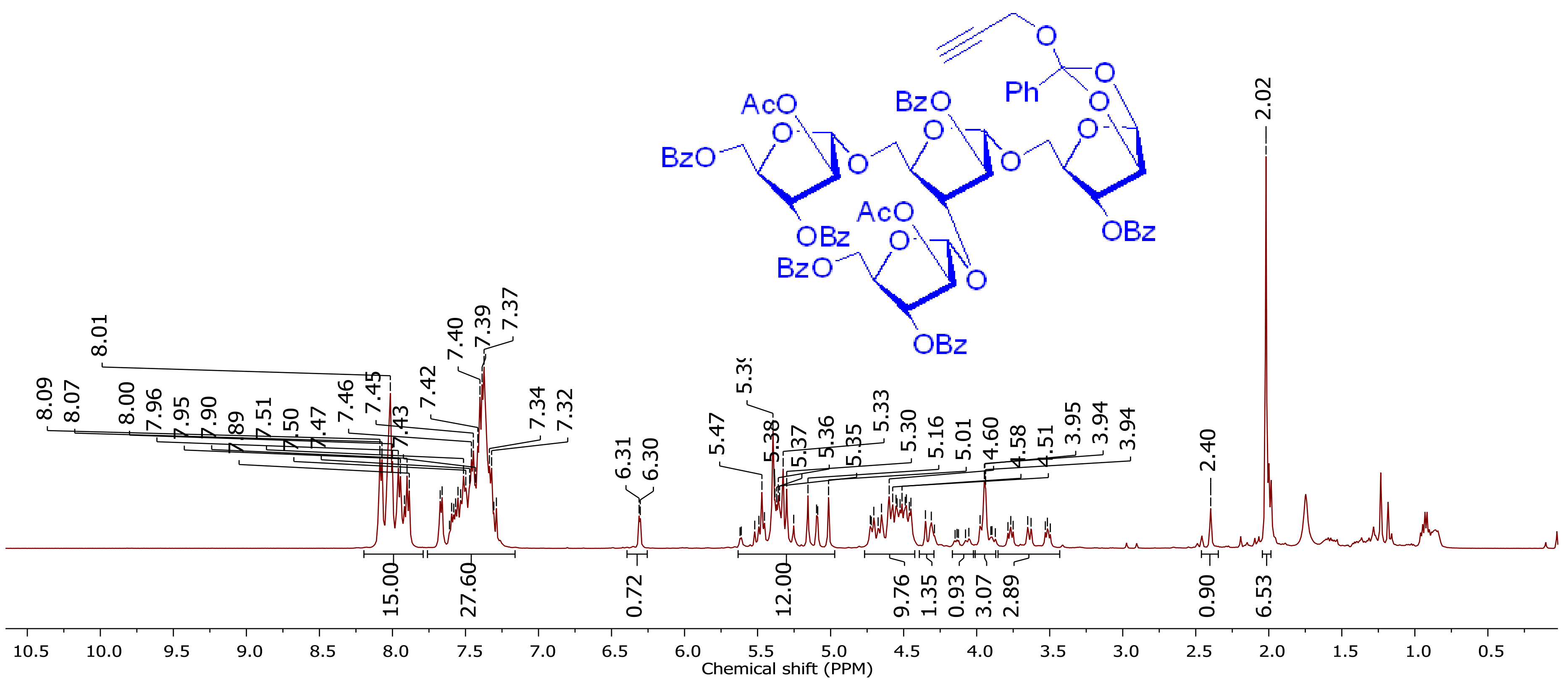

Supplementary Figure 56. ^{13}C NMR Spectrum (100.53 MHz, $CDCl_3$) of Compound **2**

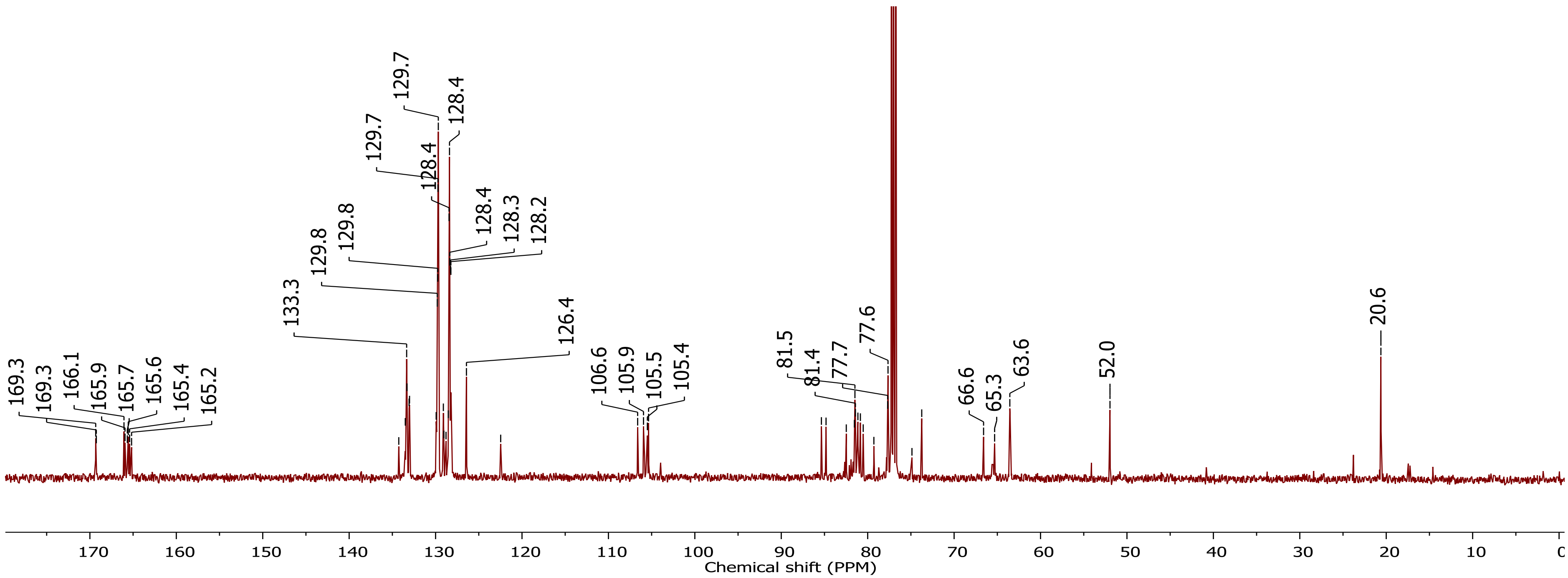

Supplementary Figure 57. DEPT NMR Spectrum (100.53 MHz, $CDCl_3$) of Compound **2**

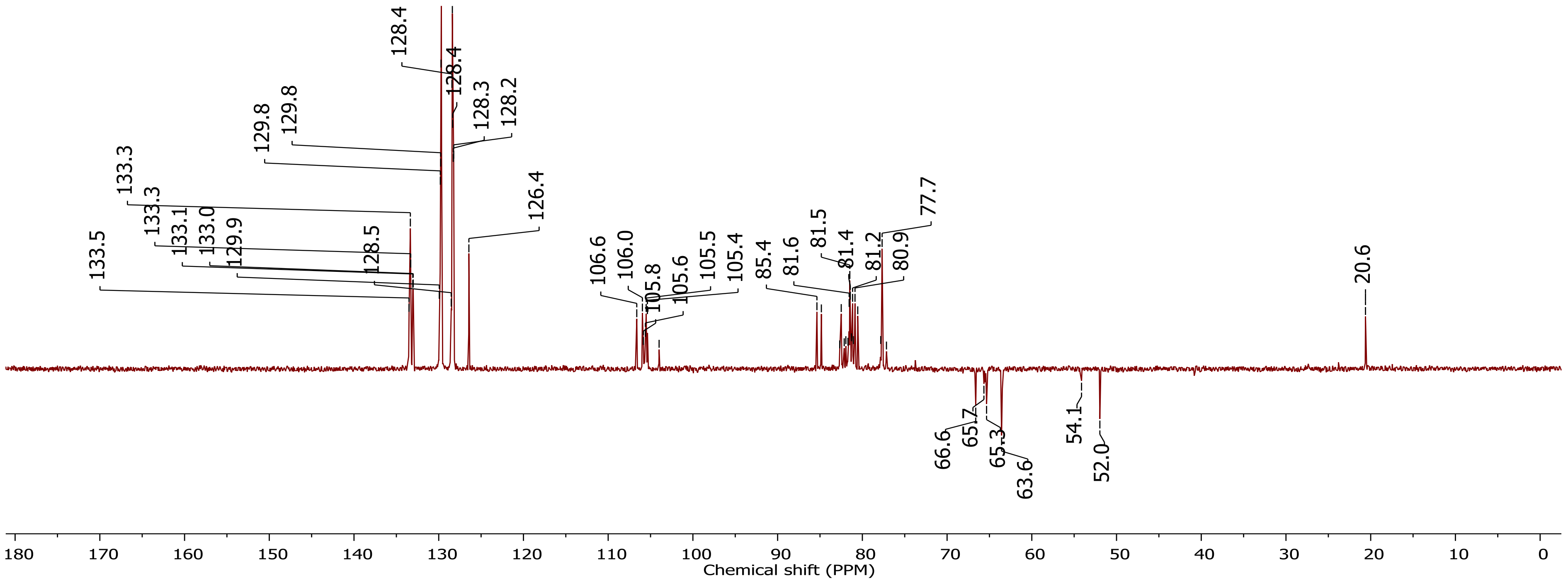

Supplementary Figure 58. ^{1}H NMR Spectrum (399.78 MHz, $CDCl_3$) Of Compound **30**

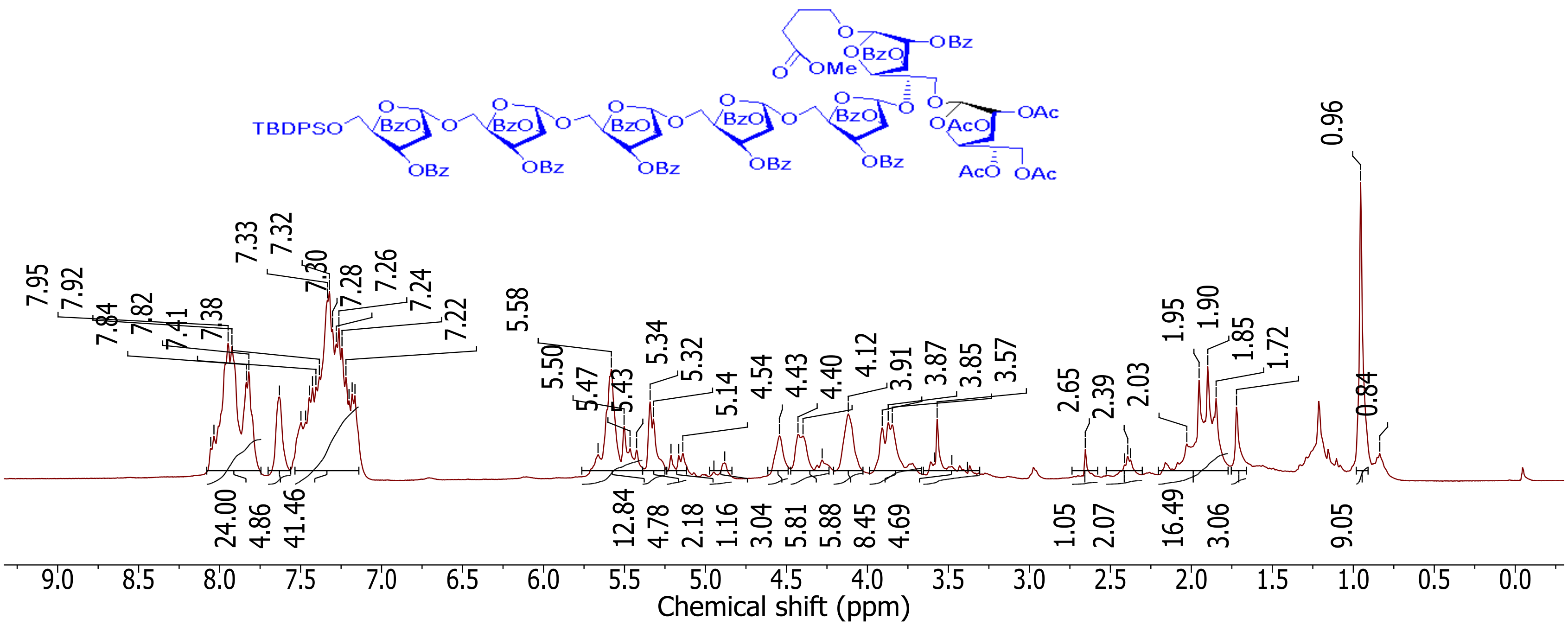

Supplementary Figure 59. ^{13}C NMR Spectrum (100.53 MHz, $CDCl_3$) Of Compound **30**

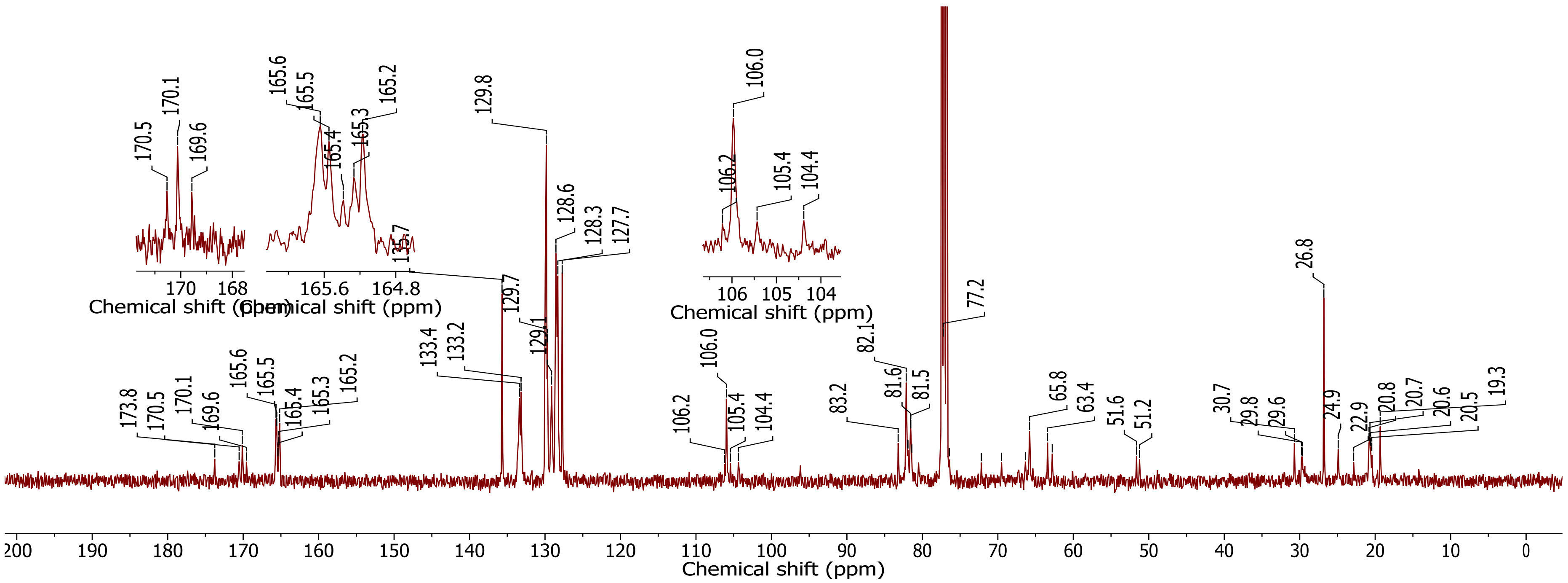

Supplementary Figure 60. DEPT NMR Spectrum (100.53 MHz, $CDCl_3$) Of Compound **30**

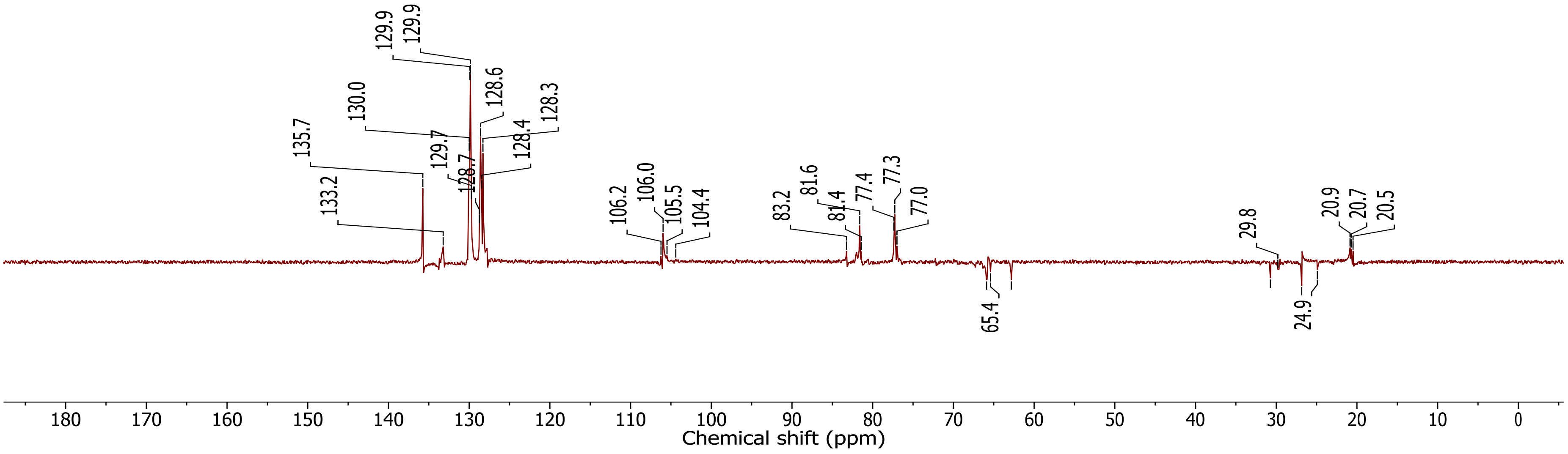

Supplementary Figure 61. ^{1}H NMR Spectrum (399.78 MHz, $CDCl_3$) of Compound **5**

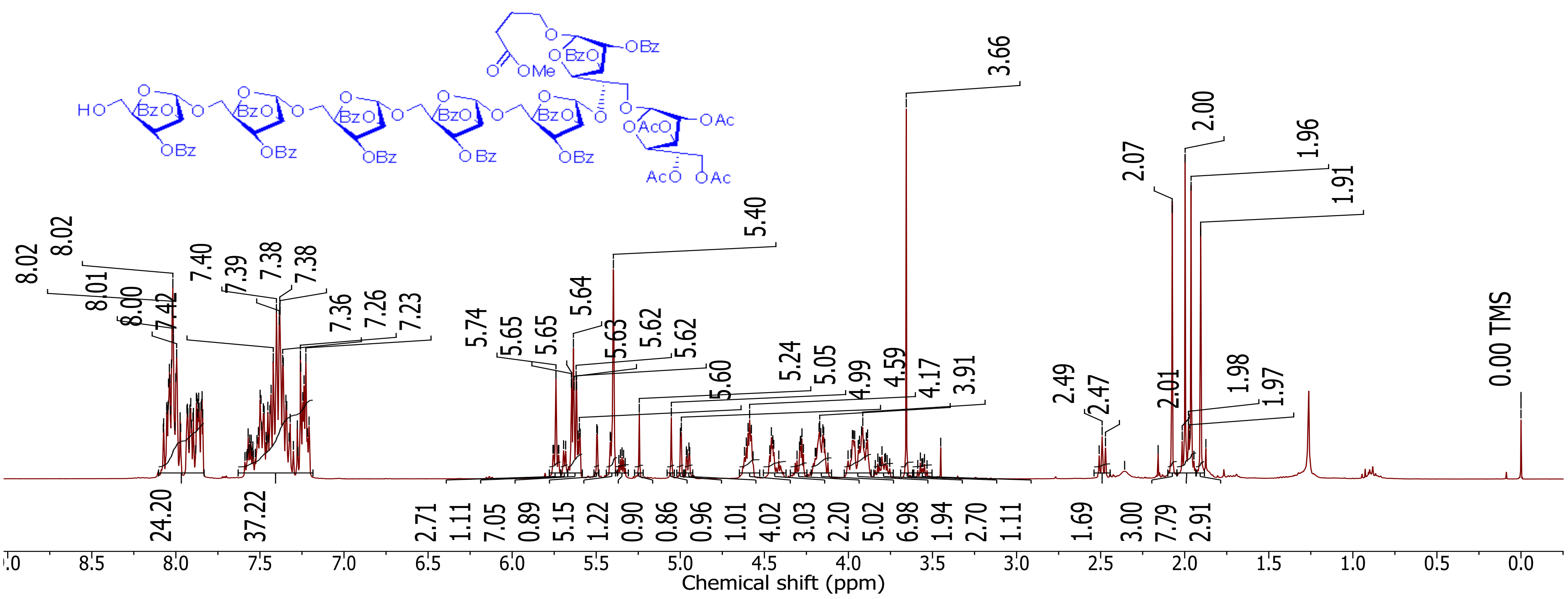

Supplementary Figure 62. ^{13}C NMR Spectrum (100.53 MHz, $CDCl_3$) of Compound **5**

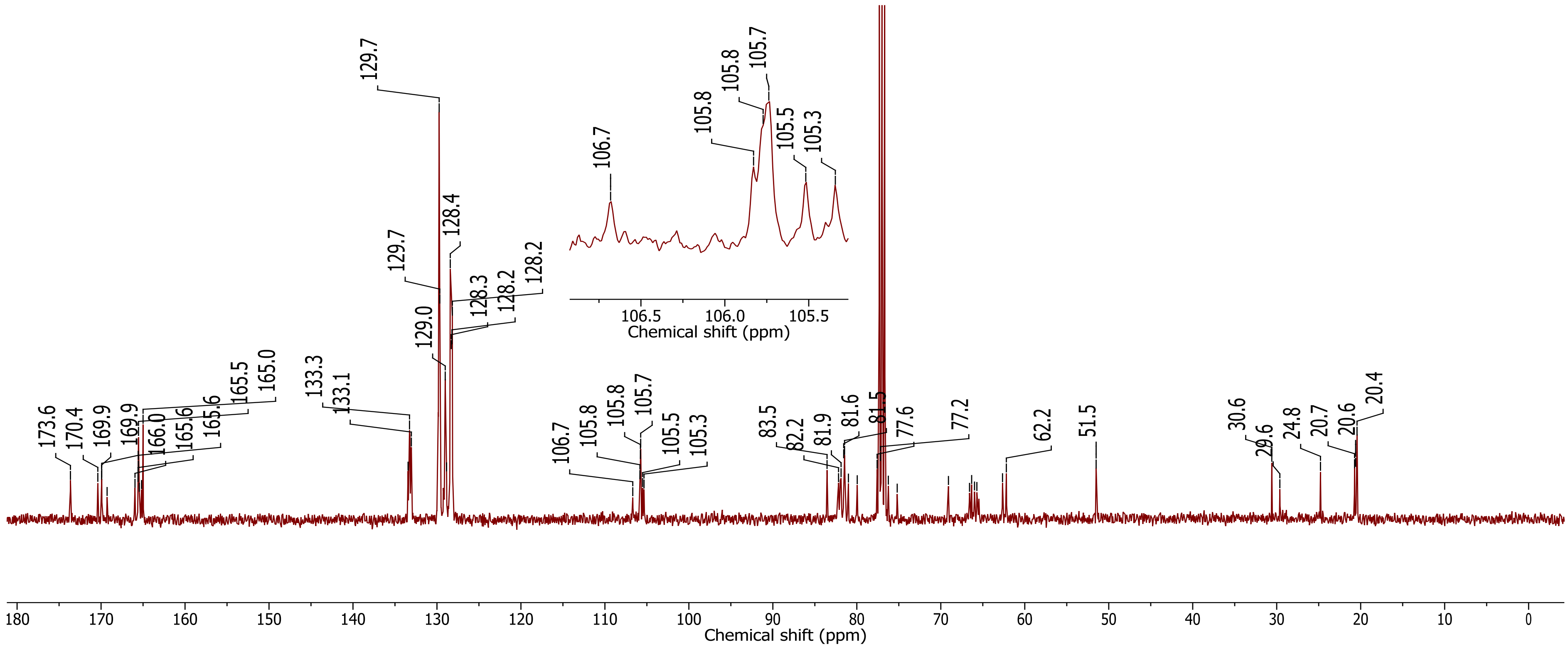

Supplementary Figure 63. DEPT NMR Spectrum (100.53 MHz, $CDCl_3$) of Compound **5**

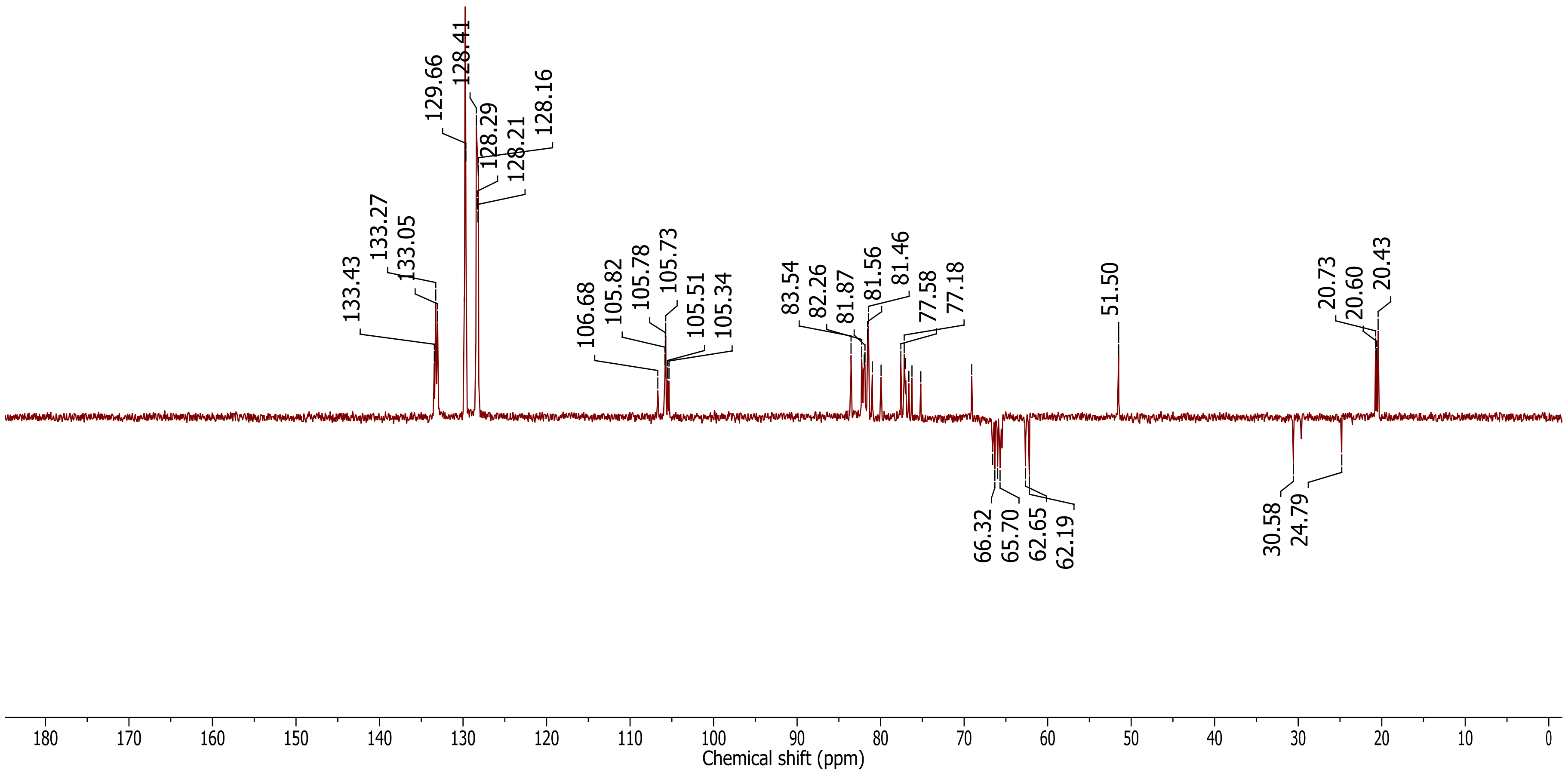

Supplementary Figure 64. ^{1}H NMR Spectrum (399.78 MHz, $CDCl_3$) of Compound **31**

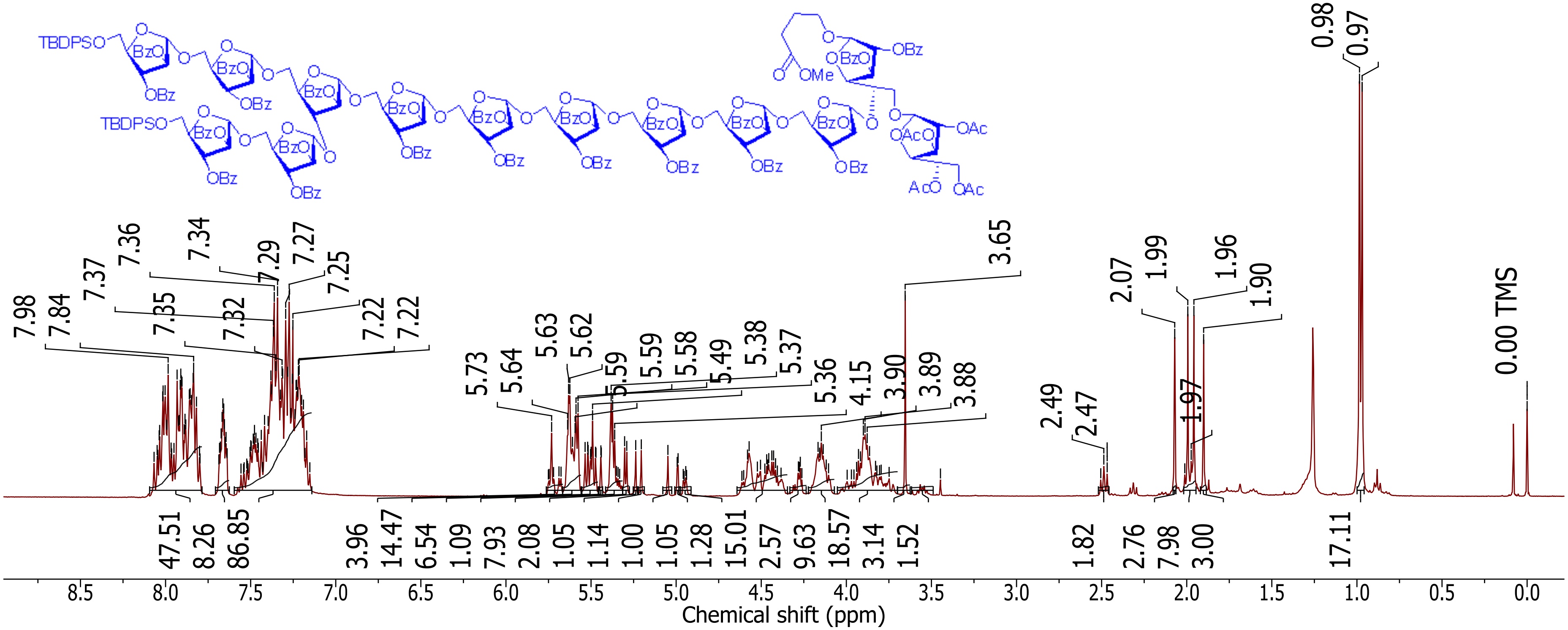

Supplementary Figure 65. ^{13}C NMR Spectrum (100.53 MHz, $CDCl_3$) of Compound **31**

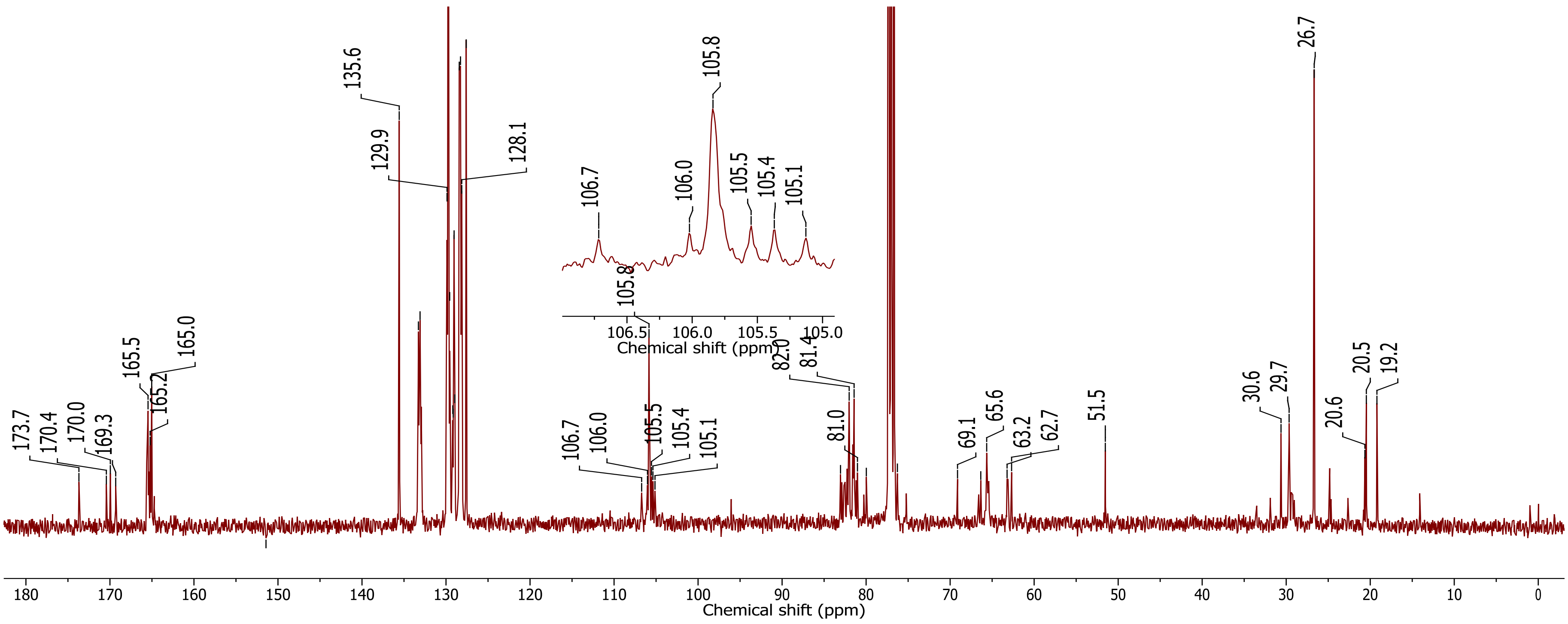

Supplementary Figure 66. DEPT NMR Spectrum (100.53 MHz, $CDCl_3$) of Compound **31**

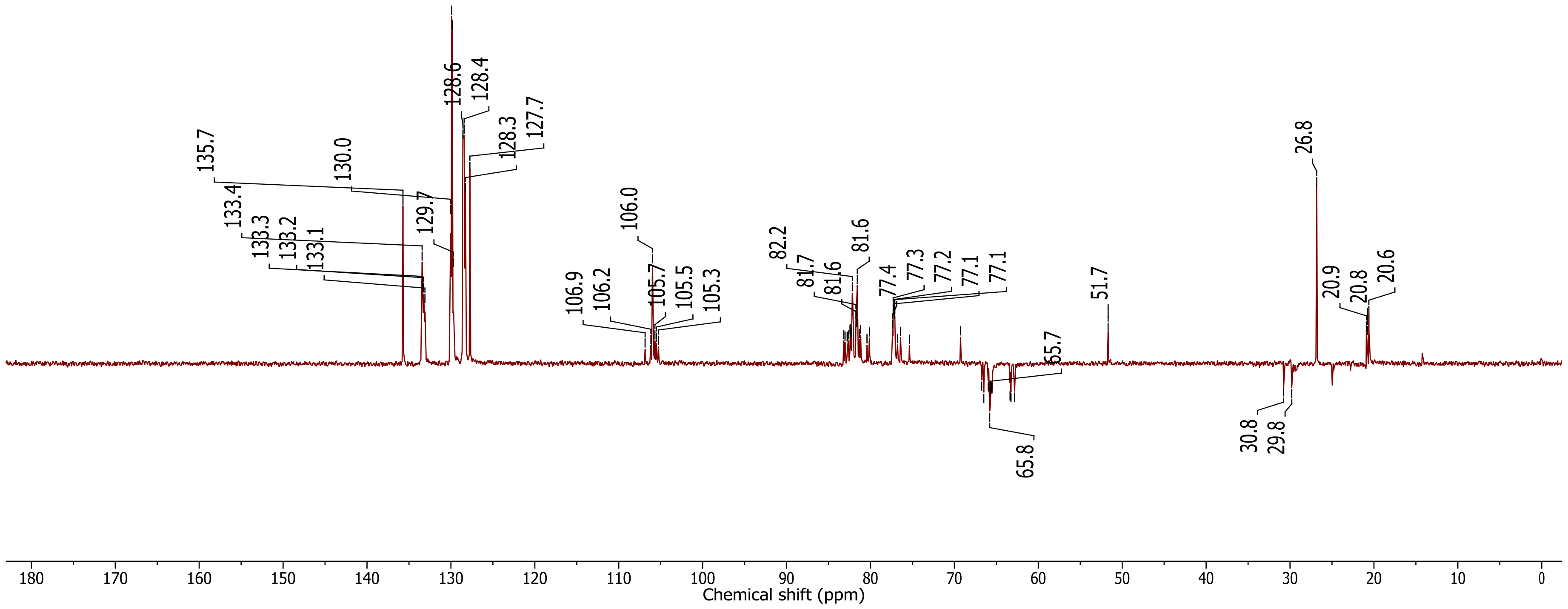

Supplementary Figure 67. ^{1}H NMR Spectrum (399.78 MHz, $CDCl_3$) of Compound **3**

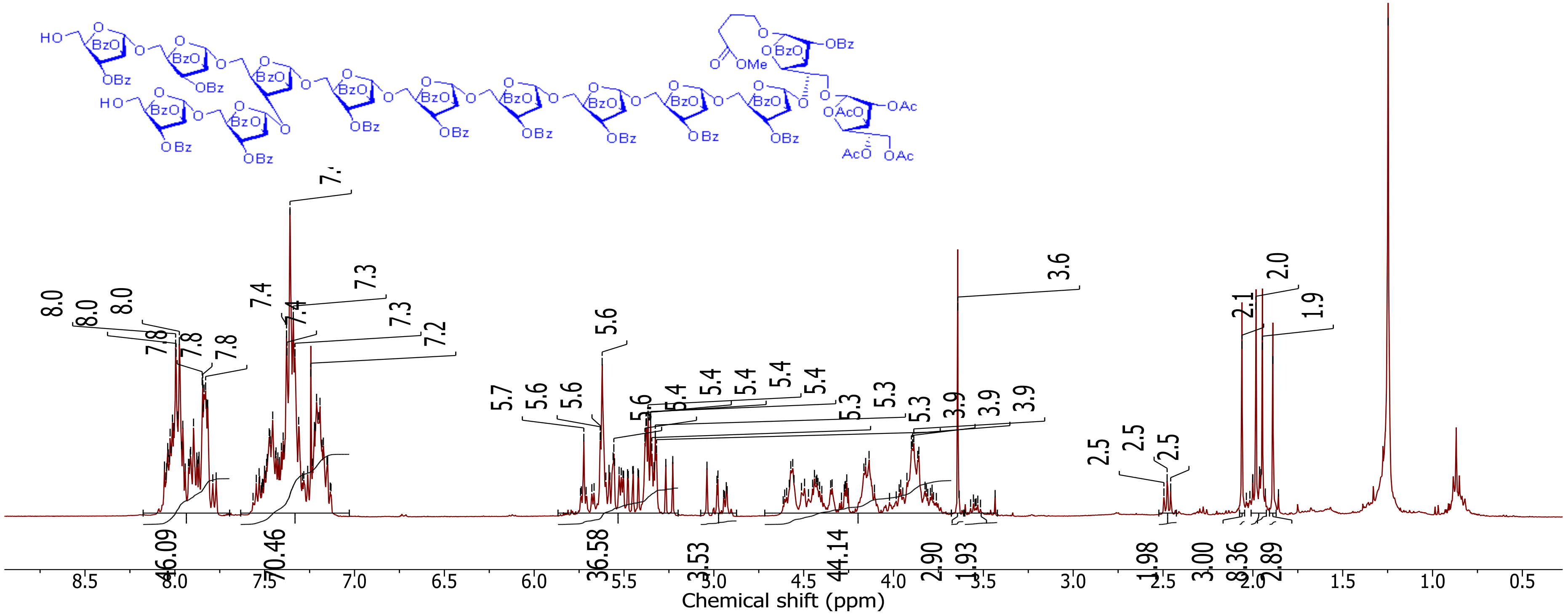

Supplementary Figure 68. ^{13}C NMR Spectrum (100.53 MHz, $CDCl_3$) of Compound **3**

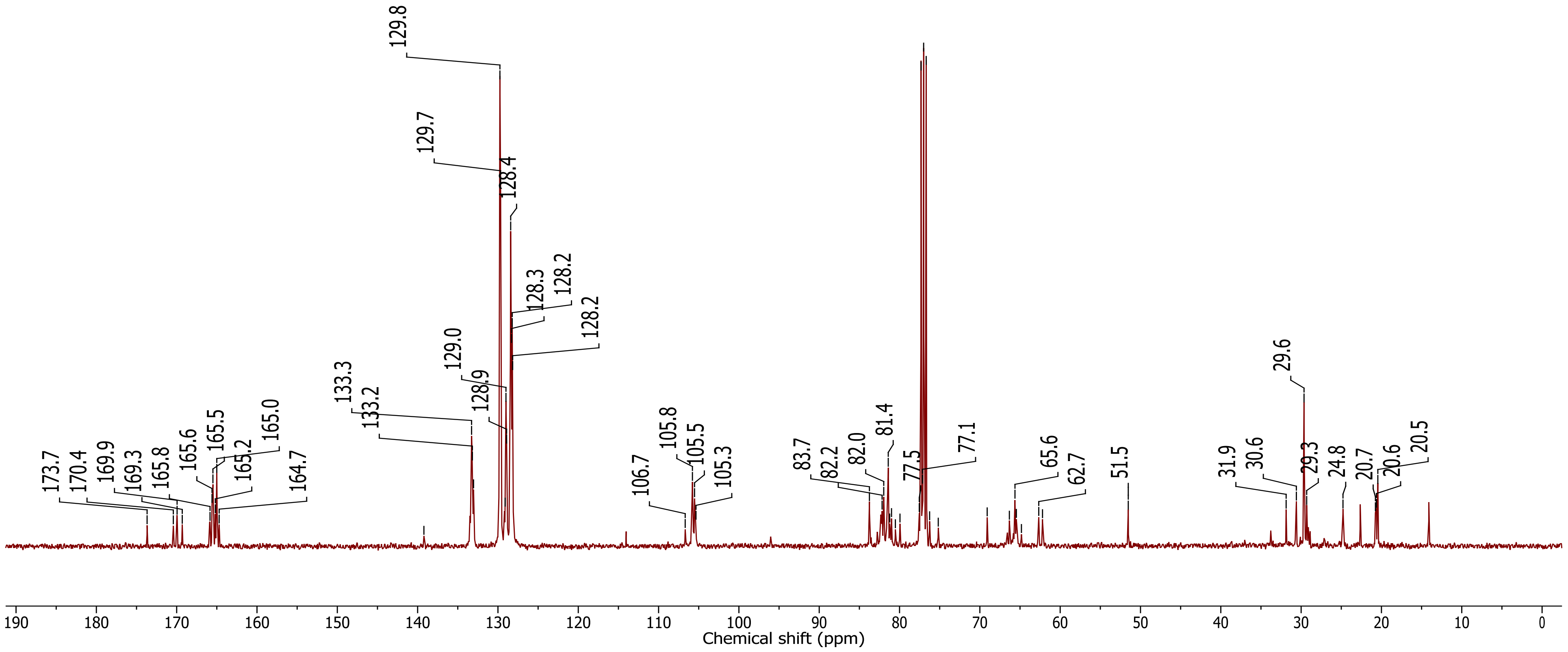

Supplementary Figure 69. DEPT NMR Spectrum (100.53 MHz, $CDCl_3$) of Compound **3**

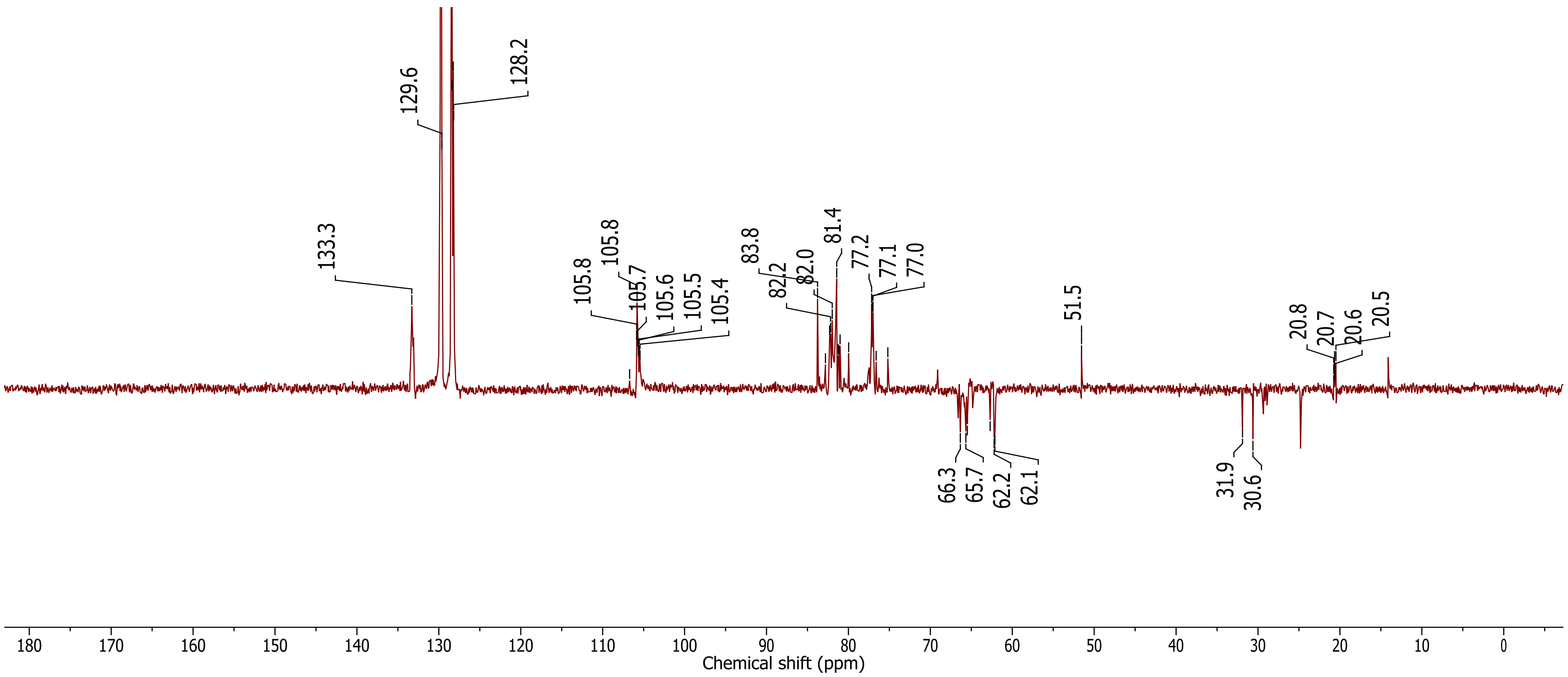

Supplementary Figure 70. ^{1}H NMR (400.13 MHz, $CDCl_3$) spectrum of **32**

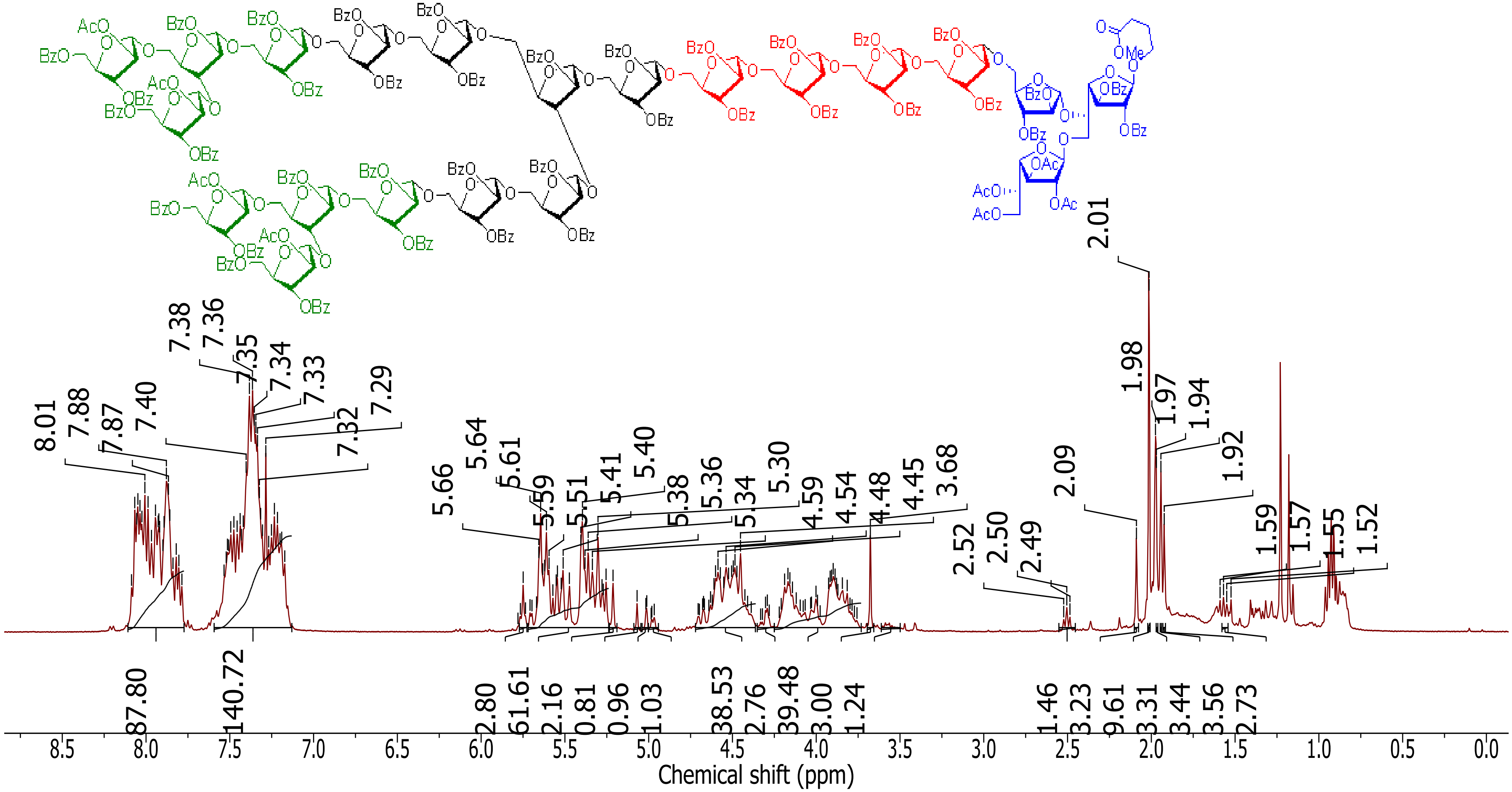

Supplementary Figure 71. ^{13}C NMR (100.61 MHz, $CDCl_3$) spectrum of **32**

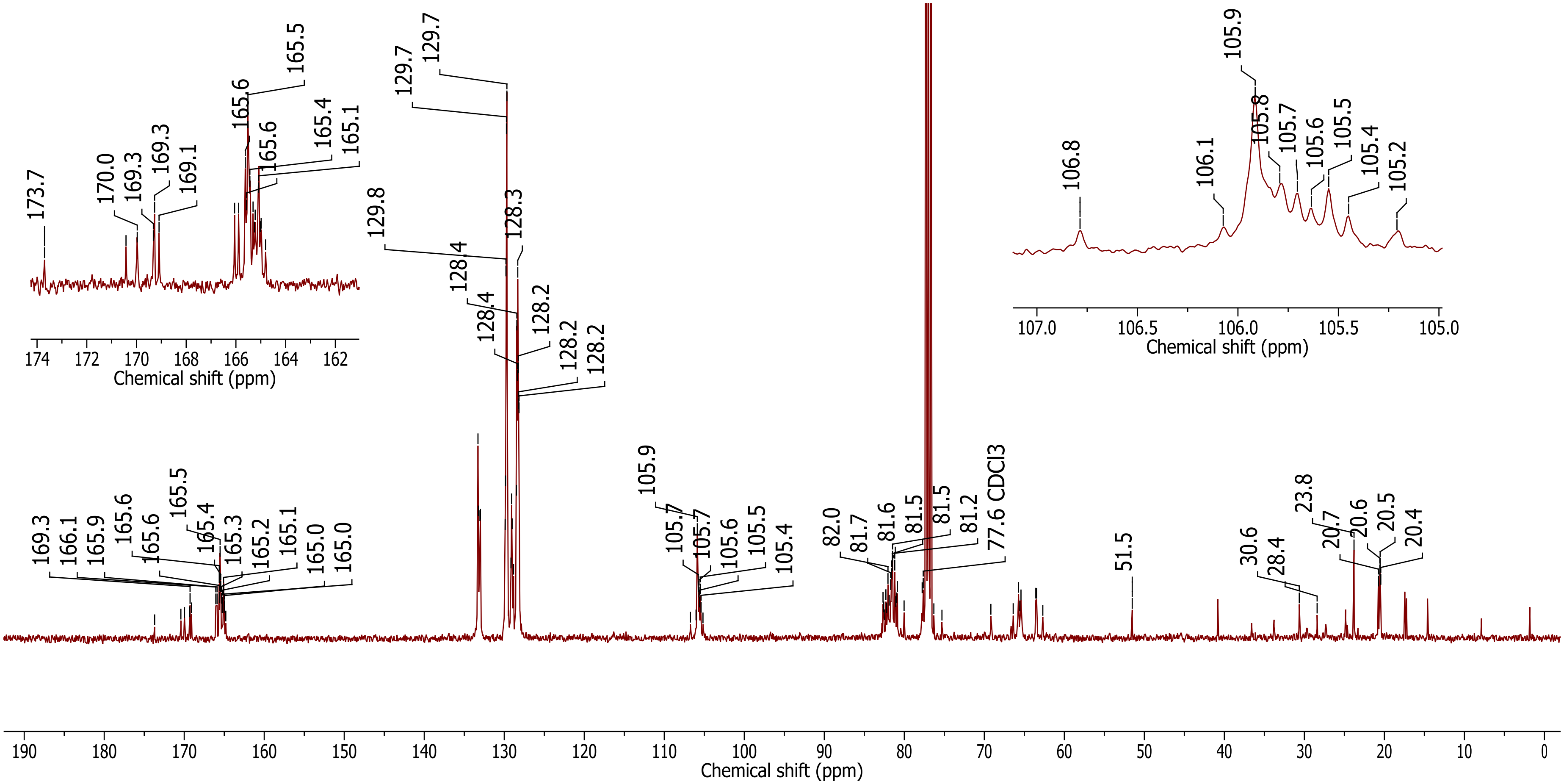

Supplementary Figure 72. ^{1}H NMR (600.40 MHz, D_2O) spectrum of **1**

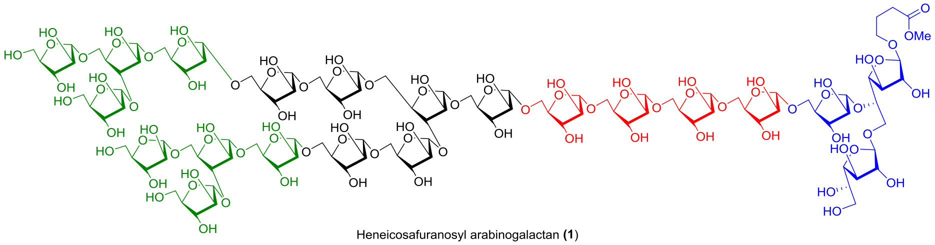

Heneicosafuranosyl arabinogalactan (**1**)

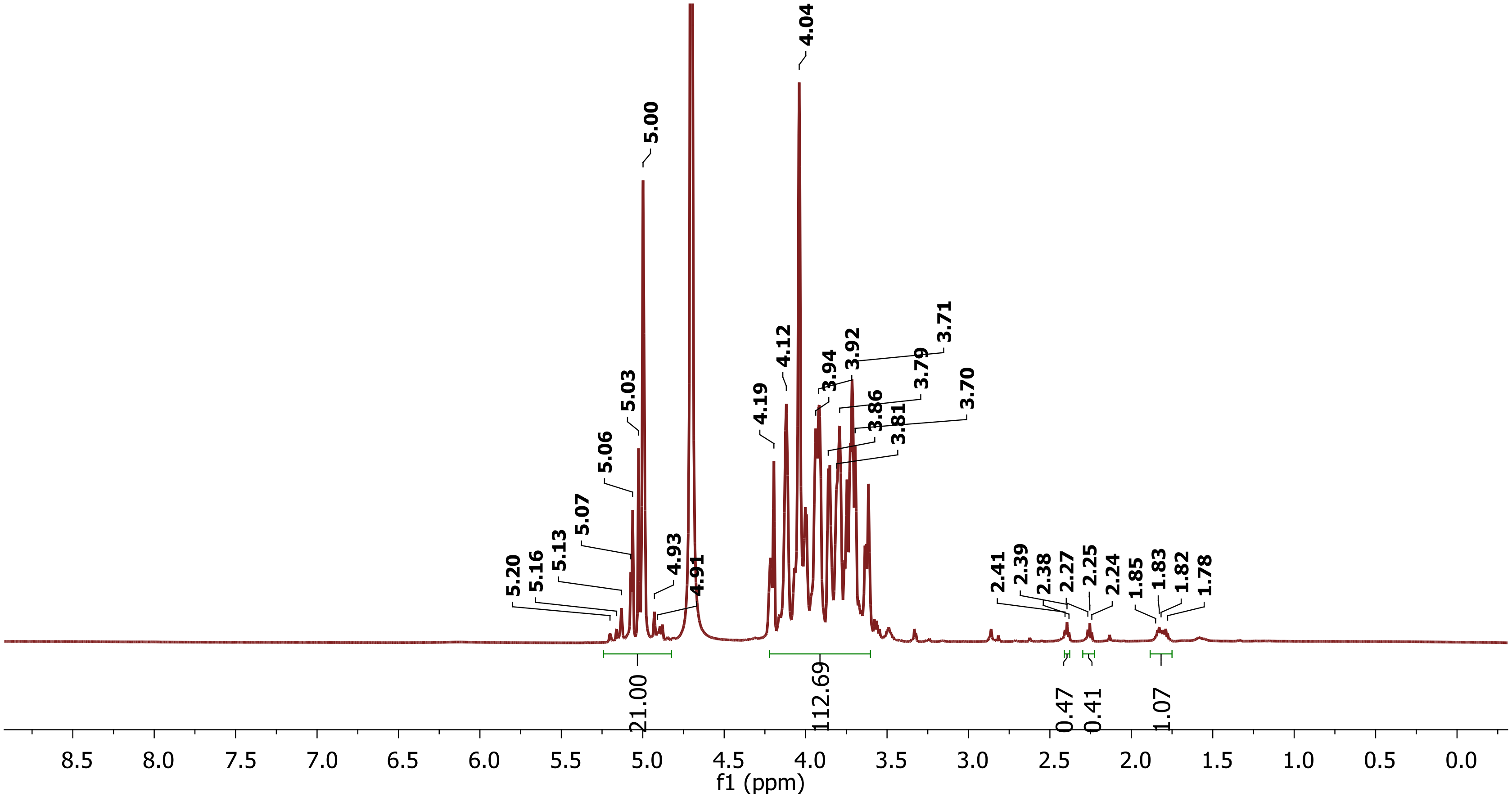

Supplementary Figure 73. ^{13}C NMR (150.97 MHz, D_2O) spectrum of **1**

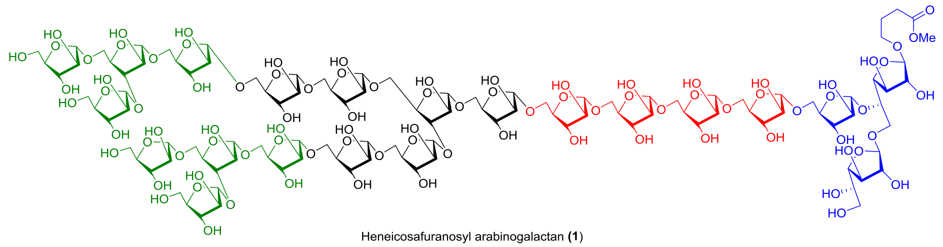

Heneicosafuranosyl arabinogalactan (**1**)

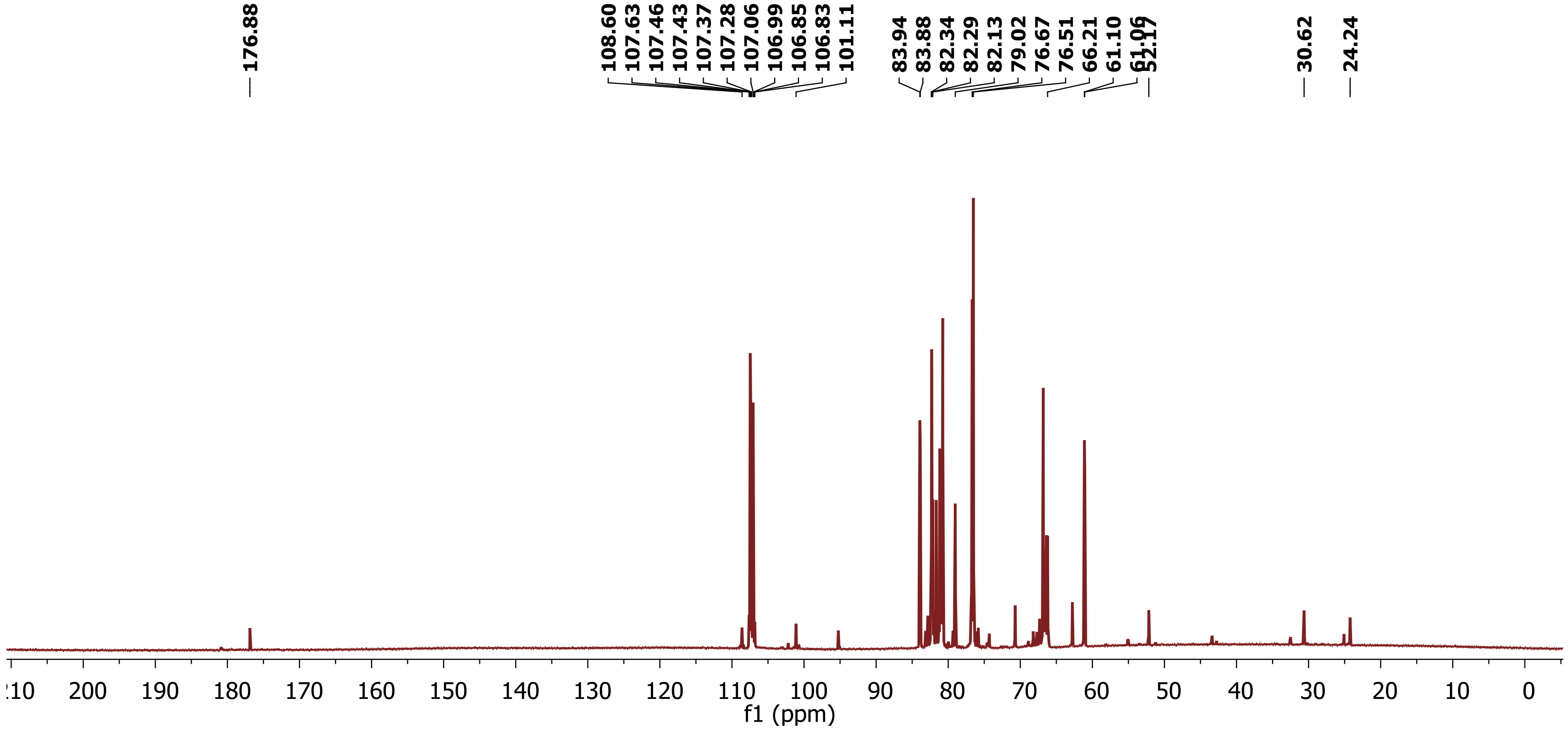

Supplementary Figure 74. MALDI-TOF spectrum of compound **1**

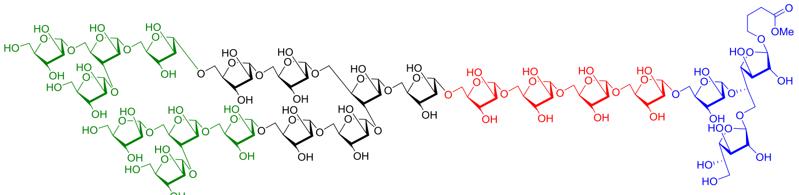

Heneicosafuranosyl arabinogalactan (**1**)

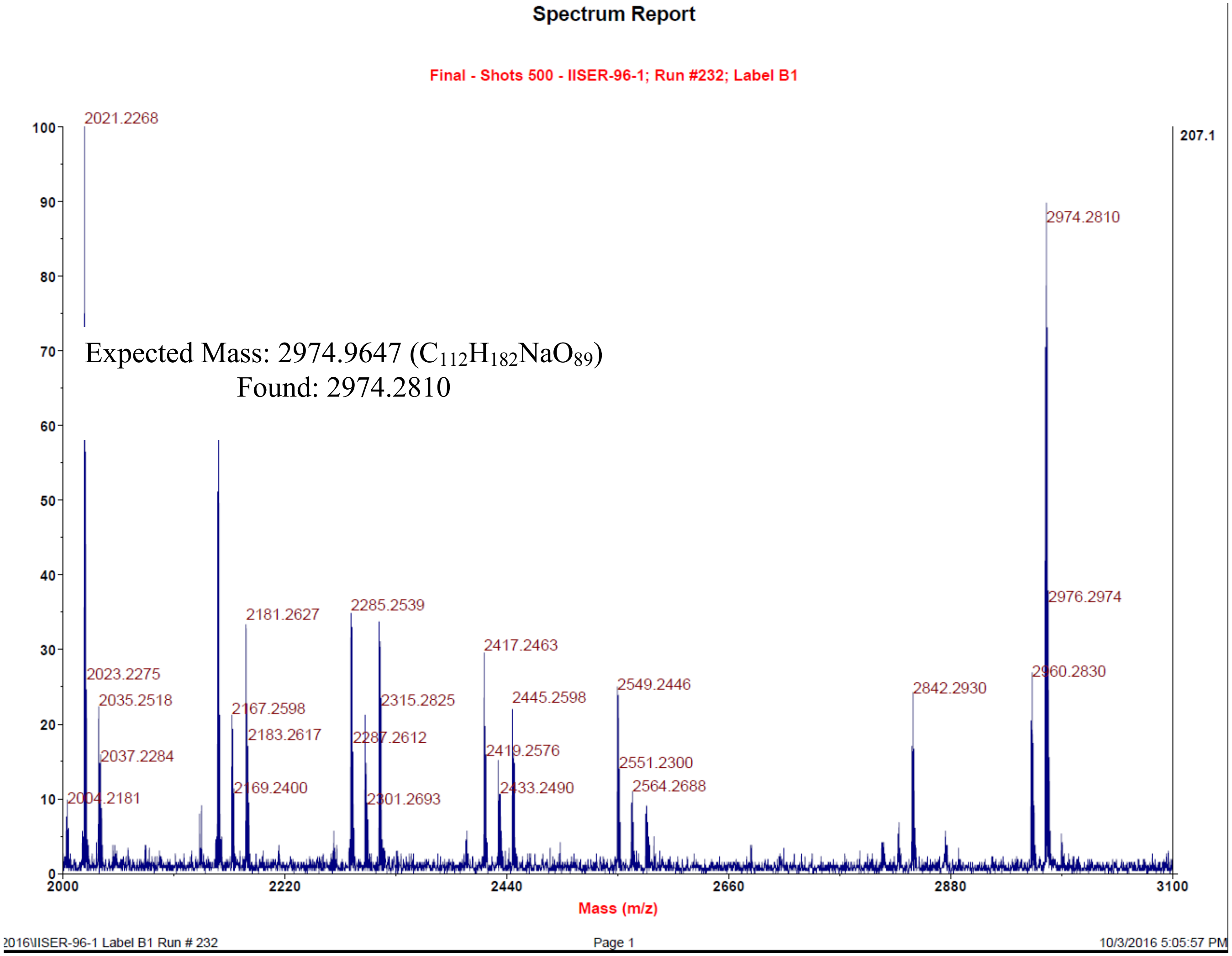

Supplementary Methods

Supplementary Method A[1]

To a CH_2Cl_2 solution (5 mL) containing glycosyl donor (0.1 to 1 mmol) and aglycon (0.1 to 1 mmol)) with 4Å molecular sieves powder (0.1 to 1.0 g) was added a catalytic amount of $AuCl_3$ (7 mol%) [AgOTf (7 mol%) as an additive wherever mentioned] and stirred at 25 ^{0}C. After 2 h (for oligosaccharides up to 24 h), the reaction mixture was neutralized by the addition of Et_3N and filtered through a pad of celite and concentrated *in vacuo*. The resulting residue was purified by silica gel column chromatography using ethyl acetate-petroleum ether to obtain 1,2-*trans* glycosides as a fluffy solids.

Supplementary Method B[2]

To a CH_2Cl_2 solution (5 mL) containing glycosyl donor (0.1 to 1 mmol) and aglycon (0.1 to 1 mmol)) with 4Å molecular sieves powder (0.1 to 1.0 g) was added 3 molar eq. of NIS at 0 ^{0}C in ice bath and stirred for 10 min. After 10 min catalytic amount of TfOH (0.3 eq.) was added to the reaction mixture at 0 °C and stirred at 25 ^{0}C. After complete disappearance of the donor (adjudged by TLC), the reaction mixture was neutralized by the addition of Et_3N and filtered through a pad of celite. The filtrate was washed with sat. aqueous solutions of sodium bicarbonate and sodium thiosulphate. Combined organic layers were dried over sodium sulphate and concentrated *in vacuo*. The resulting residue was purified by flash silica gel column chromatography using ethyl acetate-petroleum ether to obtain 1,2-*trans* glycosides.

Supplementary Method C[3]

Pent-4-enyl furanoside (1 to 50 mmol) was dissolved in anhydrous CH_2Cl_2 (10 to 500 mL) and cooled to 0 ^{0}C. Br_2 (1.1 molar eq.) in CH_2Cl_2 was added dropwise to the reaction mixture with constant stirring at 0 ^{0}C. Additionally, the reaction mixture was stirred for 10 min. at 0 ^{0}C and concentrated under reduced pressure to give furanosyl bromide as white foam which was immediately used in the next step without further purification.

The crude furanosyl bromide was redissolved (10 to 500 mL) in anhydrous CH_2Cl_2, propargyl alcohol (1.5 to 2 molar eq.) and 2, 6-lutidine (2 to 3 molar eq). Catalytic amount of tetra *n*-butyl ammonium iodide was added to the reaction and stirred for 4 h to overnight at room temperature. The reaction mixture was diluted with CH_2Cl_2 (100 to 500 mL) and water (100 to 500 mL) and the aqueous layer was extracted with CH_2Cl_2 (2x), the organic extract was washed with saturated oxalic acid solution and saturated sodium bicarbonate solution. The organic phase was collected, dried over sodium sulphate and concentrated *in vacuo*. Crude residue of the orthoester was purified by silica gel column chromatography (EtOAc:petroleum ether) to obtain propargyl 1,2-*O*-orthoester as a white foam/solid.

Supplementary Method D[3]

To a solution of *O*-TBDPS protected saccharide (0.1 to 10 mmol) in THF:py (10:2 to 100:20 mL) was added HF·py (2 molar eq. per *O*-TBDPS) and the reaction mixture was stirred at 25 ^{0}C for 4 h to 12 h. The reaction was arrested by adding saturated aqueous solution of $NaHCO_3$ and extracted with EtOAc and washed with dil.HCl. The EtOAc layer was dried over sodium sulphate and concentrated *in vacuo*. The crude residue was purified by flash column chromatography (EtOAc:petroleum ether).

Supplementary Method E

A 0.5 M NaOMe in MeOH (2 mL) was added to a solution of compound **32** (110 mg, 15.4 µmol) in 1:1 MeOH-CH_2Cl_2 (4 mL) and stirred at 25 ^{0}C. After 4 h, the reaction mixture was quenched by the addition of Amberlite®-IR120 (H^+) resin, filtered and the filtrate was concentrated *in vacuo* to obtain a residue that was washed sequentially with chloroform and ethyl acetate to remove majority of the methyl benzoate. The remaining residue was purified by column chromatography using Biogel®-P4 gel (90-180 µm, exclusion limit 4000 Da). The compound was collected using Millipore water, concentrated *in vacuo* and further lyophilized for 24 h to obtain the heneicosafuranosyl arabinogalactan **1** (34 mg, 74%) as a white solid.

Compound Characterization Data

Prop-2-yn-1-yl 3-*O*-benzoyl-5-*O*-(*t*-butyldiphenylsilyl)-α-D-arabinofuranoside 1,2-*O*-orthobenzoate **8b**

This compound is prepared using the reported procedure[3,4] using orthoester **11** (50.00 g, 99.90 mmol), NaOMe (1.08 g, 19.98 mmol) in MeOH, followed by TBDPSCl (30.20 g, 109.9 mmol), Im. (7.48 g, 109.9 mmol, in DMF, and followed by BzCl, (28.08 g, 199.9 mmol) in pyridine. Yield: (38.68 g, 61% : 3 steps); $[\alpha]_D^{25}$ ($CHCl_3$, *c* 1.2): –20.0°; IR (cm^{-1}, $CHCl_3$): 3293, 3066, 2939, 1727, 1595, 1453, 1266, 1106, 704; ^1H NMR (399.78 MHz, $CDCl_3$): δ 0.99 (s, 9H), 2.37 (t, J = 2.2 Hz, 1H), 3.63 (dd, J = 7.5, 4.1 Hz, 2H), 3.84 – 4.02 (m, 2H), 4.46 (t, J = 7.5 Hz, 1H), 5.09 (d, J = 4.3 Hz, 1H), 5.67 (s, 1H), 6.33 (d, J = 4.3 Hz, 1H), 6.97 – 7.86 (m, 18H), 8.09 (d, J = 7.9 Hz, 2H); ^{13}C NMR (100.53 MHz, $CDCl_3$): δ 19.1, 26.7, 26.7, 26.7, 51.9, 63.4, 73.7, 77.5, 79.3, 85.0, 87.2, 106.5, 122.4, 126.3, 126.3, 127.5, 127.6, 127.6, 127.6, 128.2, 128.3, 128.4, 128.4, 129.2, 129.6, 129.6, 129.6, 129.8, 129.8, 133.1, 133.1, 133.4, 134.2, 135.3, 135.4, 135.4, 135.4, 165.1; HRMS (ESI) : m/z calcd for $[C_{38}H_{38}O_7Si+Na]^+$: 657.2284; Found: 657.2281.

Pent-4-enyl 2,3-di-*O*-benzoyl-α-D-arabinofuranoside **8a**

This compound is prepared using the supplementary methods A and D using orthoester **8b** (15.00 g, 15.75 mmol) as the starting material, pent-4-en-1-ol (2.04 g, 23.63 mmol), $AuCl_3$ (0.34 g, 1.10 mmol) in dry CH_2Cl_2, followed by HF.Py (1.80 g, 63.01 mmol). Yield: 4.43 g, 66% over 2 steps; $[\alpha]_D^{25}$ ($CHCl_3$, *c* 1.0): –32.8 °; IR(cm^{-1}, $CHCl_3$): 3512, 3071, 2931, 1723, 1601, 1451, 1265, 1176, 710; ^1H NMR (399.78, $CDCl_3$): δ 1.60 – 1.94 (m, 2H), 2.08 – 2.31 (m, 2H), 2.43 (bs, 1H), 3.54 (dt, J = 9.5, 6.2 Hz, 1H), 3.79 (dt, J = 9.5, 6.6 Hz, 1H), 3.99 (d, J = 11.7 Hz, 2H), 4.32 (q, J = 4.1 Hz, 1H), 4.89 – 5.08 (m,

2H), 5.23 (s, 1H), 5.40 – 5.48 (m, 1H), 5.53 (d, J = 1.3 Hz, 1H), 5.82 (ddt, J = 16.9, 10.2, 6.6 Hz, 1H), 7.24 – 7.77 (m, 6H), 7.79 – 8.35 (m, 4H); ^{13}C NMR (100.53 MHz, $CDCl_3$): δ 28.7, 30.2, 62.3, 66.7, 77.8, 81.7, 83.6, 105.5, 114.9, 128.4, 128.4, 128.5, 128.5, 129.0, 129.1, 129.7, 129.8, 129.9, 129.9, 133.5, 133.5, 138.0, 165.3, 166.1; HRMS (ESI): m/z calcd for $[C_{24}H_{26}O_7+Na]^+$: 449.1576; Found: 449.1573.

Prop-2-yn-1-yl 3,5-Di-*O*-(*t*-butyldiphenylsilyl)-α-D-arabinofuranoside 1,2-*O*-orthobenzoate **8d**

This compound is prepared by the reported procedure[3,4] using orthoester **11** (25.00 g, 49.95 mmol) as the starting material, NaOMe (0.54 g, 9.99 mmol) in MeOH, followed by excess TBDPSCl (30.20 g, 109.88 mmol), imidazole (7.48 g, 109.88 mmol) in DMF. Yield: 29.54 g, 74% over 3 steps. $[\alpha]_D^{25}$ ($CHCl_3$, *c* 1.0): –3.0°; IR (cm^{-1}, $CHCl_3$): 3298, 3065, 2936, 2114, 1594, 1466, 1272, 1110, 701; ^1H NMR (399.78 MHz, $CDCl_3$): δ 0.84 (s, 9H), 1.09 (s, 9H), 2.27 (t, J = 2.4 Hz, 1H), 3.17 (dd, J = 10.4, 8.5 Hz, 1H), 3.34 (dd, J = 10.3, 6.4 Hz, 1H), 3.79 (dABq, J = 15.5, 2.4 Hz, 2H), 3.92 (s, 1H), 4.42 (s, 1H), 4.83 (d, J = 4.2 Hz, 1H), 6.26 (d, J = 4.2 Hz, 1H), 6.99 – 7.52 (m, 21H), 7.59 – 7.75 (m, 4H); ^{13}C NMR (100.53 MHz, $CDCl_3$): δ 19.0, 19.1, 26.7, 26.7, 26.7, 26.8, 26.8, 26.9, 51.7, 63.6, 73.5, 77.1, 79.5, 87.1, 90.2, 106.6, 122.0, 126.3, 126.3, 127.4, 127.5, 127.5, 127.5, 127.8, 127.8, 127.8, 128.1, 128.1, 129.3, 129.4, 129.5, 129.5, 129.9, 130.0, 132.8, 133.0, 133.2, 133.3, 134.5, 135.4, 135.4, 135.4, 135.4, 135.7, 135.7, 135.7, 135.7; HRMS (ESI) : m/z calcd for $[C_{47}H_{52}O_6Si_2+Na]^+$: 791.3200; Found: 791.3218

Prop-2-yn-1-yl 3,5,6-tri-*O*-acetyl-α-D-galactofuranoside 1,2-*O*-orthoacetate **9**

This compound is prepared using the above mentioned supplementary method C starting with pentenyl glycosides **17**[2] (10.00 g, 24.01 mmol), Br_2 (4.22 g, 26.92 mmol), propargyl alcohol (3.37 g,

60.04 mmol), and 2,6-lutidine (7.22 g, 60.04 mmol) in anhydrous CH_2Cl_2 (100 mL). Yield: (7.51 g, 81%); $[\alpha]_D^{25}$ ($CHCl_3$, *c* 1.2): –31.8 ^{0}; IR (cm^{-1}, $CHCl_3$): 2927, 1740, 1226, 1009; ^1H NMR (399.78 MHz, $CDCl_3$): δ 1.75 (s, 3H), 2.06 (s, 3H), 2.10 (s, 3H), 2.11 (s, 3H), 2.42 (t, *J* = 2.4 Hz, 1H), 4.14 (d, *J* = 2.5 Hz, 2H), 4.17 (dd, *J* = 12.0, 6.0 Hz, 1H), 4.19 (dd, *J* = 7.8, 2.5 Hz, 1H), 4.41 (dd, *J* = 12.2, 3.9 Hz, 1H), 4.76 (d, *J* = 4.0 Hz, 1H), 5.11 (d, *J* = 2.5 Hz, 1H), 5.27 (ddd, *J* = 7.8, 6.0, 4.0 Hz, 1H), 6.00 (d, *J* = 4.0 Hz, 1H); ^{13}C NMR (100.53 MHz, $CDCl_3$): δ 20.6 (2C), 20.8, 21.8, 51.0, 62.7, 69.7, 73.7, 76.3, 79.5, 83.6, 84.5, 105.2, 124.1, 169.6, 169.9, 170.4, HRMS (ESI): m/z calcd for $[C_{17}H_{22}O_{10}+Na]^+$: 409.1111; Found: 409.1118.

[3-(Methoxycarbonyl) propyl] 2,3,5,6-tetra-*O*-acetyl-β-D-galactofuranoside **18**

This compound is prepared using the aforementioned supplementary method A using methyl 4-hydroxybutanoate (2.29 g, 19.41 mmol) and **9** (5.00 g, 12.94 mmol) as the starting substrates, $AuCl_3$ (0.27 g, 0.91 mmol), and AgOTf (0.23 g, 0.91 mmol) in anhydrous CH_2Cl_2 (50 mL). Yield: 4.64 g, 80%. $[\alpha]_D^{25}$ ($CHCl_3$, *c* 1.2): –31.8°; IR (cm^{-1}, $CHCl_3$): 2945, 1742, 1229, 1050, 754; ^1H NMR (399.78 MHz, $CDCl_3$): δ 1.81 (q, J = 6.6, 6.0 Hz, 2H), 1.97 (s, 3H), 2.00 (s, 3H), 2.01 (s, 3H), 2.04 (s, 3H), 2.32 (t, J = 7.3 Hz, 2H), 3.39 (dt, J = 9.8, 5.9 Hz, 1H), 3.58 (s, 3H), 3.59 – 3.64 (m, 1H), 4.06 – 4.17 (m, 2H), 4.24 (dd, J = 11.8, 4.4 Hz, 1H), 4.85 – 4.94 (m, 3H), 5.19 – 5.43 (m, 1H); ^{13}C NMR (100.53 MHz, $CDCl_3$): δ 20.5, 20.6, 20.6, 20.7, 24.6, 30.4, 51.4, 62.5, 66.2, 69.1, 76.4, 79.8, 81.2, 105.2, 169.5, 169.9, 169.9, 170.4, 173.5; HRMS (ESI): m/z calcd for $[C_{19}H_{28}O_{12}+Na]^+$: 471.1478; Found: 471.1478.

[3-(Methoxycarbonyl) propyl] 2,3,-di-*O*-benzoyl-β-D-galactofuranoside **10**

This compound is prepared using the reported[1,2] procedure from compound **18** (4.00 g, 8.92 mmol), NaOMe (0.19 g, 3.57 mmol) in MeOH, followed by 2-methoxy propene (0.80 g, 11.15 mmol), PTSA (0.15 g, 0.89 mmol) in CH_2Cl_2, followed by BzCl, (5.02 g, 35.68 mmol) in pyridine, and then PTSA (0.15 g, 0.89 mmol) in MeOH. Yield: 3.05 g, 70% over 4 steps. $[\alpha]_D^{25}$ ($CHCl_3$, *c* 1.7): +28.9°; IR (cm^{-1}, $CHCl_3$): 3116, 2938, 1721, 1591, 1448, 1265, 1112, 712; ^1H NMR (399.78 MHz, $CDCl_3$): δ 1.88 (quintet, J = 6.8 Hz, 2H), 2.37 (t, J = 7.4 Hz, 2H), 2.54 (bs, 1H), 2.85 (d, J = 7.1 Hz, 1H), 3.42 – 3.55 (m, 1H), 3.56 (s, 3H), 3.64 – 3.82 (m, 3H), 4.03 (bs, 1H), 4.19 – 4.29 (m, 1H), 5.15 (s, 1H), 5.39 (s, 1H), 5.51 (d, J = 4.7 Hz, 1H), 7.30 – 7.42 (m, 4H), 7.44 – 7.59 (m, 2H), 7.90 – 8.04 (m, 4H); ^{13}C NMR (100.53 MHz, $CDCl_3$): δ 24.9, 30.5, 51.6, 64.0, 66.2, 70.7, 77.8, 81.4, 83.7, 105.6, 128.5, 128.5, 128.5, 128.5, 129.0, 129.0, 129.8, 129.8, 129.8, 129.9, 133.5, 133.5, 165.3, 166.0, 173.8; HRMS (ESI): m/z calcd for $[C_{25}H_{28}O_{10}+Na]^+$: 511.1580; Found: 511.1579.

[3-(Methoxycarbonyl) propyl] 2,3-di-*O*-benzoyl-6-*O*-(2,3,5,6-tetra-*O*-acetyl-β-D-galactofuranosyl)-β-D-galactofuranoside **19**

This compound is prepared using the above-mentioned supplementary method A using aglycon **10** (1.90 g, 3.88 mmol) and **9** (1.50 g, 3.88 mmol), $AuCl_3$ (82.43 mg, 0.27 mmol), and AgOTf (69.83 mg, 0.27 mmol) in anhydrous CH_2Cl_2 (50 mL). Yield: 2.23 g, 70%. $[\alpha]_D^{25}$ ($CHCl_3$, *c* 1.0): –8.6°; IR (cm^{-1}, $CHCl_3$): 3478, 3070, 2927, 1737, 1591, 1447, 1231, 1110, 715; ^1H NMR (399.78 MHz, $CDCl_3$): δ 1.95 – 2.02 (m, 2H), 2.05 (s, 7H), 2.08 (s, 3H), 2.13 (s, 3H), 2.48 (t, J = 7.4 Hz, 2H), 3.58 (dt, J = 9.6, 6.0 Hz, 1H), 3.67 (s, 3H), 3.70 (dd, J = 10.5, 7.2 Hz, 1H), 3.81 (dt, J = 9.6, 6.3 Hz, 1H), 3.86 (dd, J = 10.4, 4.5 Hz, 1H), 4.20 (dd, J = 11.9, 7.2 Hz, 1H), 4.26 – 4.33 (m, 3H), 4.36 (dd, J = 11.9, 4.2 Hz, 1H), 5.02 (dd, J = 5.7, 1.8 Hz, 1H), 5.07 (d, J = 1.7 Hz, 1H), 5.11 (s, 1H), 5.26 (s, 1H), 5.39 (dt, J = 7.6, 4.0 Hz, 1H), 5.48 – 5.49 (m, 1H), 5.66 (d, J = 4.1 Hz, 1H), 7.42 – 7.54 (m, 4H), 7.54 – 7.65 (m, 2H), 8.01 – 8.13 (m, 4H); ^{13}C NMR (100.53 MHz, $CDCl_3$): δ 20.6, 20.7, 20.7, 20.8, 24.9, 30.6, 51.6, 62.7, 66.3,

69.0, 69.3, 69.5, 76.3, 77.8, 80.2, 81.3, 81.5, 83.2, 105.7, 106.0, 128.5-129.9 (10C), 133.6, 133.6, 165.4, 165.9, 169.7, 170.0, 170.1, 170.6, 173.7; HRMS (ESI): m/z calcd for $[C_{39}H_{46}O_{19}+Na]^+$: 841.2531; Found: 841.2533.

[3-(Methoxycarbonyl) propyl] 2,3-di-*O*-benzoyl-5-*O*-(2,3-di-*O*-benzoyl-5-*O*-(*t*-butyldiphenylsilyl)-α-D-arabinofuranosyl)-6-*O*-(2,3,5,6-tetra-*O*-acetyl-β-D-galactofuranosyl)-β-D-galactofuranoside **20**

This compound is prepared using the above-mentioned supplementary method A using aglycon **19** (2.00 g, 2.44 mmol) and donor **8b** (1.71 g, 2.69 mmol), $AuCl_3$ (51.86 mg, 0.17 mmol), and AgOTf (43.93 mg, 0.17 mmol) in anhydrous CH_2Cl_2 (50 mL). Yield: 2.56 g, 75%. $[\alpha]_D^{25}$ ($CHCl_3$, *c* 1.2): +12.4°; IR (cm^{-1}, $CHCl_3$): 3070, 2927, 1727, 1600, 1450, 1269, 1110, 709; ^1H NMR (399.78 MHz, $CDCl_3$): δ 1.04 (s, 9H), 1.77 (s, 3H), 1.91 (s, 3H), 1.94 (s, 3H), 1.99 (s, 3H), 2.09 (dd, J = 10.0, 8.3 Hz, 2H), 2.44 (t, J = 7.7 Hz, 2H), 3.54 (dt, J = 9.8, 6.0 Hz, 1H), 3.61 (s, 3H), 3.62 – 3.67 (m, 1H), 3.78 (dt, J = 9.6, 6.3 Hz, 1H), 3.85 (dd, J = 10.3, 5.3 Hz, 1H), 3.92 – 3.97 (m, 1H), 3.98 (d, J = 3.9 Hz, 1H), 4.08 – 4.19 (m, 2H), 4.31 – 4.40 (m, 2H), 4.42 – 4.52 (m, 3H), 4.97 (dd, J = 6.1, 2.5 Hz, 1H), 4.97 – 5.08 (m, 1H), 5.21 (s, 1H), 5.25 (s, 1H), 5.31 (dt, J = 7.4, 3.9 Hz, 1H), 5.43 – 5.51 (m, 1H), 5.52 (s, 1H), 5.69 (dd, J = 16.2, 4.6 Hz, 2H), 7.24 – 7.61 (m, 18H), 7.66 – 7.76 (m, 4H), 7.93 – 8.18 (m, 8H); ^{13}C NMR (100.53 MHz, $CDCl_3$): δ 19.4, 20.4, 20.6, 20.6, 20.8, 24.9, 26.8, 26.8, 26.8, 30.7, 51.6, 62.8, 63.1, 66.3, 66.9, 69.4, 72.2, 76.4, 77.1, 77.3, 80.4, 81.4, 81.9, 82.1, 82.7, 83.0, 104.4, 105.4, 106.3, 127.7 (4C), 128.4-128.8 (8C), 129.2-129.5 (4C), 127.2-130.0 (10C), 133.2, 133.2, 133.4, 133.4, 133.5, 133.6, 135.7 (4C), 165.5, 165.5, 165.6, 165.7, 169.5, 170.1, 170.1, 170.5, 173.8; HRMS (ESI): m/z calcd for $[C_{74}H_{80}O_{25}Si+Na]^+$: 1419.4656; Found: 1419.4651.

[3-(Methoxycarbonyl) propyl] 2,3-di-*O*-benzoyl-5-*O*-(2,3-di-*O*-benzoyl-α-D-arabinofuranosyl)-6-*O*-(2,3,5,6-tetra-*O*-acetyl-β-D-galactofuranosyl)-β-D-galactofuranoside **7**

This compound is prepared using the abovementioned supplementary method D from **20** (2.00 g, 1.43 mmol and HF.Py (70% HF basis, 81.80 mg, 2.86 mmol) in anhydrous THF:Py (20:4 mL). Yield: 1.58 g, 95%. $[\alpha]_D^{25}$ ($CHCl_3$, *c* 1.0): –9.7°; IR (cm^{-1}, $CHCl_3$): 3393, 2924, 1727, 1591, 1449, 1261, 1107, 715; ^1H NMR (399.78 MHz, $CDCl_3$): δ 1.94 (s, 3H), 1.97 (s, 3H), 1.99 (s, 3H), 1.99 (m, 2H), 2.07 (s, 3H), 2.40 – 2.53 (m, 3H), 3.55 (dt, J = 9.8, 6.0 Hz, 1H), 3.64 (s, 3H), 3.71 – 3.85 (m, 2H), 3.88-3.99 (m, 3H), 4.13 (dd, J = 11.9, 7.5 Hz, 1H), 4.26 – 4.33 (m, 2H), 4.40 (m, 2H), 4.42 – 4.48 (m, 1H), 4.94 (dd, J = 6.1, 2.2 Hz, 1H), 5.01 (d, J = 2.1 Hz, 1H), 5.02 (s, 1H), 5.24 (s, 1H), 5.33 (dt, J = 7.5, 3.8 Hz, 1H), 5.46 (dd, J = 5.6, 2.1 Hz, 1H), 5.48 (d, J = 1.8 Hz, 1H), 5.55 (d, J = 2.0 Hz, 1H), 5.70 (dd, J = 5.2, 1.6 Hz, 1H), 5.72 (s, 1H), 7.28 – 7.60 (m, 10H), 7.68 (dd, J = 8.0, 1.6 Hz, 2H), 7.90 – 8.10 (m, 8H); ^{13}C NMR (100.53 MHz, $CDCl_3$): δ 20.5, 20.5, 20.6, 20.8, 24.8, 30.6, 51.5, 62.2, 62.7, 66.3, 67.2, 69.1, 75.4, 76.3, 77.0, 77.2, 77.3, 79.8, 81.2, 82.3, 82.3, 83.0, 105.3, 105.7, 106.6, 127.8-129.9 (20C), 133.3, 133.3, 133.4, 133.4, 165.2, 165.6, 165.7, 165.9, 169.6, 170.0, 170.0, 170.5, 173.7; HRMS (ESI): m/z calcd for $[C_{58}H_{62}O_{25}+Na]^+$: 1181.3478; Found: 1181.3469.

Pent-4-enyl 2,3-di-*O*-benzoyl-5-*O*-(2,3-di-*O*-benzoyl-5-*O*-(*t*-butyldiphenylsilyl)-α-D-arabinofuranosyl)-α-D-arabinofuranoside **21**[4]

This compound is prepared using the above-mentioned supplementary method A starting from aglycon **8a** (3.36 g, 6.50 mmol) and donor **8b** (5.00 g, 6.50 mmol) and $AuCl_3$ (0.17 g, 0.55 mmol) in anhydrous CH_2Cl_2 (100 mL). Yield: 6.65 g, 84%. $[\alpha]_D^{25}$ ($CHCl_3$, *c* 1.0): –12.4 °; IR(cm^{-1}, $CHCl_3$): 3070, 2927, 1727, 1594, 1451, 1268, 1115, 715; ^1H NMR (399.78, $CDCl_3$): 1.02 (s, 9H), 1.68 – 1.80 (m, 2H), 2.12 – 2.23 (m, 2H), 3.52 (dt, J = 9.0, 5.9 Hz, 1H), 3.79 (dt, J = 9.2, 6.5 Hz, 1H), 3.93 (dd, J = 11.2, 2.5 Hz, 1H), 3.99 (d, J = 4.4 Hz, 1H), 4.20 (dt, J = 11.1, 6.5 Hz, 1H), 4.33 (d, J = 2.3 Hz, 1H), 4.48 (d, J = 3.9 Hz, 1H), 4.51 (dd, J = 10.5, 5.9 Hz, 1H), 4.92 – 5.06 (m, 2H), 5.22 (s, 1H), 5.38 (s,

1H), 5.51 (s, 1H), 5.58 (s, 1H), 5.63 (s, 1H), 5.64 (s, 1H), 5.81 (ddt, J = 16.8, 10.1, 6.5 Hz, 1H), 7.14 – 7.80 (m, 22H), 7.87 – 8.19 (m, 8H); ^{13}C NMR (100.53 MHz, $CDCl_3$): 19.2, 26.7, 26.7, 26.7, 28.7, 30.3, 63.3, 66.1, 66.6, 72.4, 77.5, 81.7, 81.8, 82.1, 83.2, 105.6, 105.9, 114.9, 127.5-133.3 (30C), 135.6, 135.6, 135.6, 135.7, 135.9, 135.9, 138.1, 165.2, 165.4, 165.5, 165.6; HRMS (ESI) : m/z calcd for $[C_{59}H_{60}O_{13}Si+Na]^+$: 1027.3701; Found: 1027.3711.

Pent-4-enyl 2,3-di-*O*-benzoyl-5-*O*-(2,3-di-*O*-benzoyl-α-D-arabinofuranosyl)-α-D-arabinofuranoside **22**[4]

This compound is synthesized using the above-mentioned supplementary method D on compound **21** (3.00 g, 2.98 mmol and HF.Py (70% HF basis, 0.43 g, 14.92 mmol) in anhydrous THF:Py (30:6 mL). Yield: 2.17 g, 95%. $[\alpha]_D^{25}$ ($CHCl_3$, *c* 1.0): –19.0°; IR(cm^{-1}, $CHCl_3$): 3422, 3070, 2928, 1724, 1598, 1451, 1264, 1110, 712; ^{1}H NMR (399.78 MHz, $CDCl_3$): δ 1.40 – 1.90 (m, 2H), 2.07 – 2.34 (m, 2H), 2.39 (brs, 1H), 3.51 (dt, J = 9.5, 6.1 Hz, 1H), 3.77 (dt, J = 9.5, 6.6 Hz, 1H), 3.96 (dd, J = 11.2, 2.9 Hz, 2H), 4.02 (dd, J = 12.1, 3.5 Hz, 1H), 4.20 (dd, J = 11.2, 4.6 Hz, 1H), 4.44 (td, J = 4.6, 3.0 Hz, 1H), 4.51 (q, J = 4.0 Hz, 1H), 4.88 – 5.08 (m, 2H), 5.22 (s, 1H), 5.42 (s, 1H), 5.43 – 5.45 (m, 1H), 5.51 (d, J = 1.2 Hz, 1H), 5.62 (d, J = 5.0 Hz, 1H), 5.66 (d, J = 1.3 Hz, 1H), 5.82 (ddt, J = 16.9, 10.2, 6.7 Hz, 1H), 7.04 – 7.73 (m, 12H), 7.75 – 8.30 (m, 8H); ^{13}C NMR (100.53 MHz, $CDCl_3$): δ 28.7, 30.2, 62.2, 66.1, 66.6, 77.3, 77.7, 81.6, 81.7, 81.8, 83.6, 105.5, 105.7, 114.9, 128.3-129.8 (20C), 133.3, 133.4, 133.4, 133.5, 138.0, 165.1, 165.4, 165.7, 166.1; HRMS (ESI) : m/z calcd for $[C_{43}H_{42}O_{13}+Na]^+$: 789.2523; Found: 789.2520.

Prop-2-yn-1-yl 3-*O*-Benzoyl-5-*O*-(2,3-di-*O*-benzoyl-5-*O*-(*t*-butyldiphenylsilyl)-α-D-arabinofuranosyl)-α-D-arabinofuranoside 1,2-*O*-orthobenzoate **23**[3]

This compound is prepared using the above-mentioned supplementary method C using pentenyl glycosides **21** (3.50 g, 3.48 mmol), Br_2 (0.61 g, 3.83 mmol), propargyl alcohol (0.39 g, 6.96 mmol),

and 2,6-lutidine (1.70 g, 13.93 mmol) in anhydrous CH_2Cl_2 (25 mL). Yield: (2.89 g, 85%); $[\alpha]_D^{25}$ ($CHCl_3$, c 1.2): –18.9°; IR(cm^{-1}, $CHCl_3$): 3434, 3070, 2931, 1721, 1588, 1264, 1107, 706; ^{1}H NMR (399.78, $CDCl_3$): δ 1.01 (s, 9H), 2.37 (t, J = 2.5 Hz, 1H), 3.51 (dd, J = 10.4, 7.5 Hz, 1H), 3.72 – 3.80 (m, 1H), 3.90 – 3.96 (m, 1H), 3.96 – 4.02 (m, 1H), 4.26 (q, J = 4.4 Hz, 1H), 4.34 (d, J = 2.2 Hz, 1H), 4.44 – 4.56 (m, 1H), 4.61 (t, J = 7.4 Hz, 1H), 5.05 (s, 1H), 5.12 (d, J = 4.3 Hz, 1H), 5.34 – 5.45 (m, 1H), 5.50 – 5.68 (m, 2H), 6.36 (d, J = 4.3 Hz, 1H), 7.10 – 7.75 (m, 24H), 7.86 – 8.22 (m, 6H); ^{13}C NMR (100.53 MHz, $CDCl_3$): δ 19.3, 26.8, 26.8, 26.8, 52.0, 63.2, 66.8, 73.8, 77.2, 77.9, 79.4, 82.2, 83.2, 85.0, 85.4, 105.9, 106.8, 122.6, 126.5-130.0 (26C), 133.2, 133.3, 133.5, 133.6, 135.6, 135.7, 135.7, 135.7, 135.7, 135.7, 135.7, 136.0, 165.3, 165.4, 165.7; HRMS (ESI) : m/z calcd for $[C_{57}H_{54}O_{13}Si+Na]^+$: 997.3231; Found: 997.3238.

Pent-4-enyl 2,3-di-*O*-benzoyl-5-*O*-(2,3-di-*O*-benzoyl-5-*O*-(2,3-di-*O*-benzoyl-5-*O*-(2,3-di-*O*-benzoyl-5-*O*-(*t*-butyldiphenylsilyl)-α-D-arabinofuranosyl)-α-D-arabinofuranosyl)-α-D-arabinofuranosyl)-α-D-arabinofuranoside **24**[3]

This compound is prepared using the above mentioned supplementary method A using aglycon **22** (1.60 g, 2.09 mmol) and **23** (2.03 g, 2.09 mmol), $AuCl_3$ (37.53 mg, 0.15 mmol), and AgOTf (44.30 mg, 0.15 mmol) in anhydrous CH_2Cl_2 (40 mL). Yield: 2.95 g, 84%. $[\alpha]_D^{25}$ ($CHCl_3$, *c* 1.2): +2.4^o; IR (cm^{-1}, $CHCl_3$): 3068, 2933, 1724, 1599, 1452, 1266, 1109, 708; ^{1}H NMR (399.78 MHz, $CDCl_3$): δ 0.99 (s, 9H), 1.79 – 1.65 (m, 2H), 2.21 – 2.09 (m, 2H), 3.50 (dt, J = 9.5, 6.2 Hz, 1H),3.76 (dt, J = 9.5, 6.6 Hz, 1H), 3.98 – 3.85 (m, 6H), 4.17 (ddd, J = 14.7, 7.9, 3.2 Hz, 3H), 4.42 (dd, J = 7.6, 4.5 Hz, 1H), 4.47 (q, J = 4.5 Hz, 1H), 4.64 – 4.56 (m, 2H), 5.04 – 4.90 (m, 2H), 5.21 (s, 1H), 5.35 (s, 1H), 5.39 (s, 1H), 5.40 (s, 1H), 5.48 (t, J = 2.1 Hz, 1H), 5.53 (t, J = 4.0 Hz, 1H), 5.65 – 5.59 (m, 5H), 5.80 (ddt, J = 16.9, 10.2, 6.7 Hz, 1H), 7.59 – 7.19 (m, 30H), 7.67 (m, 4H), δ 8.06 – 7.86 (m, 16H); ^{13}C NMR (100.53 MHz,

$CDCl_3$): δ 19.4, 26.9, 26.9, 26.9, 28.9, 30.4, 60.6, 63.4, 65.8, 65.9, 66.0, 66.8, 66.8, 77.3, 77.4, 81.7, 81.7, 81.9, 81.9, 82.1, 82.1, 82.3, 83.2, 105.7, 105.9, 106.0, 106.0, 115.0, 127.8-130.1 (50C), 133.2, 133.3, 133.3, 133.3, 133.5, 133.5, 133.5, 133.5, 135.8, 135.8, 138.2, 165.3, 165.3, 165.4, 165.6, 165.6, 165.7, 165.7, 165.8; HRMS (ESI): m/z calcd for $[C_{97}H_{92}O_{25}Si+Na]^+$: 1708.5628; Found: 1708.5620.

Prop-2-ynyl 3-*O*-Benzoyl-5-*O*-(2,3-di-*O*-benzoyl-5-*O*-(2,3-di-*O*-benzoyl-5-*O*-(2,3-di-*O*-benzoyl-5-*O*-(*t*-butyldiphenylsilyl)-α-D-arabinofuranosyl)-α-D-arabinofuranosyl)-α-D-arabinofuranosyl)-α-D-arabinofuranoside 1,2-*O*-orthobenzoate **6**[3]

This compound is prepared using the aforementioned supplementary method C using pentenyl glycosides **24** (2.50 g, 1.48 mmol), Br_2 (0.26 g, 1.63 mmol), propargyl alcohol (0.21 g, 3.71 mmol), and 2,6-lutidine (0.45 g, 3.71 mmol) in anhydrous CH_2Cl_2 (25 mL). Yield: 2.16 g, 88%. $[\alpha]_D^{25}$ ($CHCl_3$, *c* 1.0): –2.1°; IR(cm^{-1}, $CHCl_3$): 3302, 3071, 2927, 2105, 1725, 1596, 1454, 1265, 1107, 708; ^{1}H NMR (399.78 MHz, $CDCl_3$): δ 1.00 (s, 9H), 2.36 (t, J = 2.5 Hz, 1H), 3.51 (dd, J = 10.3, 7.2 Hz, 1H), 3.67 (dd, J = 11.2, 2.7 Hz, 1H), 3.78 (dd, J = 10.3, 7.7 Hz, 1H), 3.88 (dd, J = 9.5, 2.5 Hz, 1H), 3.92 (dd, J = 5.2, 2.4 Hz, 2H), 3.96 (d, J = 4.5 Hz, 2H), 4.07 (dd, J = 11.2, 4.0 Hz, 1H), 4.17 (dd, J = 11.3, 4.1 Hz, 1H), 4.28 (dd, J = 7.4, 3.9 Hz, 1H), 4.49 (q, J = 4.6, 1H), 4.58-4.62 (m, 2H), 5.56 (d, J = 1.3 Hz, 1H), 5.61-5.63 (m, 3H), 5.11 (d, J = 4.3 Hz, 1H), 5.30 (s, 1H), 5.45 (d, J = 1.2 Hz, 1H), 5.52 (s, 1H), 5.06 (s, 1H), 5.38 (s, 1H), 5.60 (s, 1H), 6.35 (d, J = 4.3 Hz, 1H), 7.21-7.70(m, 36H), 7.87-8.05 (m, 14H); ^{13}C NMR (100.53 MHz, $CDCl_3$): δ 19.2, 26.7 (3C), 52.0, 63.3, 65.5, 65.7, 66.4, 76.7, 77.2, 77.3, 77.7, 81.4, 81.5, 82.0, 82.1, 82.1, 82.1, 83.1, 84.8, 85.3, 105.4, 105.8, 105.9, 106.7, 122.5, 126.4 (2C), 127.6-135.6 (59C), 165.0, 165.1, 165.2 (2C), 165.4, 165.6 (2C); HRMS (ESI): m/z calcd for $[C_{95}H_{86}O_{25}Si+Na]^+$: 1678.5159; Found: 1678.5146.

Pent-4-enyl 2,3-di-*O*-benzoyl-5-*O*-(2-*O*-benzoyl-3,5-di-*O*-((*t*-butyldiphenyl)silyl)-α-D-arabinofuranosyl)-α-D-arabinofuranoside **25**

This compound is prepared using the aforementioned supplementary method A using aglycon **8a** (7.5 g, 8.80 mmol) and donor **8d** (13.64 g, 17.60 mmol), and $AuCl_3$ (0.19 g, 0.60 mmol) in anhydrous CH_2Cl_2 (100 mL). Yield: 8.12 g, 81%. $[\alpha]_D^{25}$ ($CHCl_3$, c 1.0): +27.8°; IR (cm^{-1}, $CHCl_3$): 3067, 2932, 2114, 1726, 1642, 1454, 1264, 1109, 705; ^1H NMR (399.78 MHz, $CDCl_3$): δ 0.93 (s, 9H), 0.98 (s, 9H), 1.68 – 1.83 (m, 2H), 2.10 – 2.28 (m, 2H), 3.52 (dt, J = 9.6, 6.5 Hz, 1H), 3.59 (dd, J = 11.6, 4.6 Hz, 1H), 3.69 (dd, J = 11.5, 2.6 Hz, 1H), 3.83 (dt, J = 9.5, 6.6 Hz, 1H), 3.93 (dd, J = 11.0, 4.1 Hz, 1H), 4.10 (dd, J = 11.1, 6.1 Hz, 1H), 4.36 (dq, J = 6.0, 2.7 Hz, 1H), 4.50 (dd, J = 5.9, 2.0 Hz, 1H), 4.56 (q, J = 5.1, 4.6 Hz, 1H), 4.90 – 5.07 (m, 2H), 5.10 (s, 1H), 5.23 (s, 1H), 5.32 (d, J = 2.0 Hz, 1H), 5.51 (d, J = 1.4 Hz, 1H), 5.51 – 5.59 (m, 1H), 5.82 (ddt, J = 16.9, 10.2, 6.6 Hz, 1H), 7.10 – 7.22 (m, 4H), 7.23 – 7.47 (m, 14H), 7.53 – 7.63 (m, 11H), 7.68 – 7.75 (m, 2H), 8.03 – 8.12 (m, 4H); ^{13}C NMR (100.53 MHz, $CDCl_3$): δ 19.1, 19.2, 26.7, 26.7, 26.7, 26.7, 26.8, 26.8, 28.8, 30.3, 62.7, 66.7, 66.9, 76.9, 77.8, 81.4, 81.9, 84.8, 85.0, 105.7, 106.1, 114.8, 127.5-130.0 (27C), 132.7, 132.9, 133.2, 133.2, 133.3, 133.3, 133.5, 135.6, 135.6, 135.7, 135.7, 135.7, 135.7, 135.8, 135.8, 138.2, 165.1, 165.5, 165.6; HRMS (ESI): m/z calcd for $[C_{68}H_{74}O_{12}Si_2+Na]^+$: 1161.4617; Found: 1161.4610.

Pent-4-enyl 2,3-di-*O*-benzoyl-5-*O*-(2-*O*-benzoyl-α-D-arabinofuranosyl)-α-D-arabinofuranoside **26**

This compound is prepared using the above-mentioned supplementary method D using **25** (8.00 g, 7.02 mmol and HF·py (70% HF basis, 0.80 g, 28.12 mmol) in anhydrous THF:Py (30:6 mL) as the starting material. Yield: 3.71 g, 80%. $[\alpha]_D^{25}$ ($CHCl_3$, *c* 1.0): +24.4°; IR (cm^{-1}, $CHCl_3$): 3506, 3069, 2930, 1721, 1600, 1451, 1267, 1111, 863; ^1H NMR (399.78 MHz, $CDCl_3$): δ 1.68 – 1.80 (m, 2H), 2.12

– 2.23 (m, 2H), 2.21 (bs, 1H), 3.53 (dt, J = 9.5, 6.2 Hz, 1H), 3.72 – 3.84 (m, 3H), 3.93 (ddd, J = 12.0, 6.4, 3.1 Hz, 2H), 4.14 (d, J = 5.0 Hz, 1H), 4.17 (dd, J = 10.9, 4.9 Hz, 1H), 4.27 – 4.33 (m, 1H), 4.40 (q, J = 4.8 Hz, 1H), 4.92 – 5.04 (m, 2H), 5.15 (d, J = 2.4 Hz, 1H), 5.24 (s, 1H), 5.38 (s, 1H), 5.50 (d, J = 1.4 Hz, 1H), 5.54 (d, J = 5.0 Hz, 1H), 5.81 (ddt, J = 16.9, 10.2, 6.6 Hz, 1H), 7.36 – 7.49 (m, 5H), 7.50 – 7.72 (m, 4H), 7.98 (dd, J = 8.2, 1.2 Hz, 2H), 8.06 (d, J = 7.8 Hz, 4H); ^{13}C NMR (100.53 MHz, $CDCl_3$): δ 28.7, 30.2, 62.0, 65.8, 66.8, 76.5, 77.3, 81.6, 81.9, 84.4, 86.0, 105.1, 105.6, 114.9, 128.5-129.9 (15C), 133.5, 133.5, 133.6, 138.0, 165.4, 165.9, 166.5; HRMS (ESI): m/z calcd for $[C_{36}H_{38}O_{12}+Na]^+$: 685.2261; Found: 685.2255.

Pent-4-enyl 2,3-di-*O*-benzoyl-5-*O*-(2-*O*-benzoyl -3-*O*-(2,3-di-*O*-benzoyl-5-*O*-((*t*-butyldiphenyl)silyl)-α-D-arabinofuranosyl)-5-*O*-(2,3-di-*O*-benzoyl-5-*O*-((-*t*-butyldiphenyl)silyl)-α-D-arabinofuranosyl)-α-D-arabinofuranosyl)-α-D-arabinofuranosyl)-α-D-arabinofuranoside **27**

This compound is prepared employing the above-mentioned supplementary method A on aglycon **26** (2.50 g, 3.77 mmol), **8b** (5.99 g, 9.43 mmol), $AuCl_3$ (0.16 g, 0.53 mmol), AgOTf (0.13 g, 0.53 mmol) in anhydrous CH_2Cl_2 (100 mL). Yield: (3.09 g, 45%); $[\alpha]_D^{25}$ ($CHCl_3$, *c* 1.0): +11.7°; IR (cm^{-1}, $CHCl_3$): 3293, 3071, 2974, 1723, 1594, 1450, 1268, 1107, 717; ^{1}H NMR (399.78 MHz, $CDCl_3$): δ 0.92 (s, 9H), 0.97 (s, 9H), 1.64 – 1.77 (m, 2H), 2.15 (dd, J = 8.9, 4.7 Hz, 2H), 3.48 (dt, J = 9.5, 6.2 Hz, 1H), 3.76 (dt, J = 9.6, 6.6 Hz, 1H), 3.86-3.90 (m, 6H), 4.05 (dd, J = 11.5, 4.6 Hz, 1H), 4.16 (dd, J = 11.2, 4.7 Hz, 1H), 4.31 – 4.40 (m, 2H), 4.40 (q, J = 4.6 Hz, 1H), 4.46 (d, J = 6.1 Hz, 1H), 4.51-4.54 (m, 1H), 4.85 – 5.05 (m, 2H), 5.18 (s, 1H), 5.30 (s, 1H), 5.37 (s, 1H), 5.42 (d, J = 1.2 Hz, 1H), 5.47 (dd, J = 4.2, 1.3 Hz, 2H), 5.52 (d, J = 1.2 Hz, 1H), 5.57 (td, J = 11.2, 10.2, 5.3 Hz, 4H), 5.79 (ddt, J = 16.9, 10.1, 6.7 Hz, 1H), 7.11 – 7.57 (m, 34H), 7.56 – 7.73 (m, 7H), 7.75 – 8.08 (m, 14H); ^{13}C NMR (100.53 MHz, $CDCl_3$): δ 19.2, 19.2, 26.6, 26.6, 26.7, 26.7, 26.7, 26.7, 28.7, 30.3, 63.2, 63.2, 65.8, 65.8, 66.7, 77.1, 77.1, 80.9, 81.8, 81.9, 81.9, 81.9, 82.0, 82.2, 82.6, 83.4, 83.7, 105.5, 105.5, 105.8, 106.1, 114.8,

127.6-133.0 (48C), 133.0, 133.1, 133.1, 133.2, 133.2, 133.2, 133.4, 135.5, 135.5, 135.5, 135.6, 135.6, 135.6, 135.6, 135.6, 135.6, 135.6, 135.6, 138.1, 164.9, 165.1, 165.3, 165.3, 165.4, 165.5, 165.5; HRMS (ESI): m/z calcd for $[C_{106}H_{106}O_{24}Si_2+Na]^+$: 1841.6510; Found: 1841.6523.

Pent-4-enyl 2,3-di-*O*-benzoyl-5-*O*-(2-*O*-benzoyl-3-*O*-(2,3-di-*O*-benzoyl-5-*O*-(2,3-di-*O*-benzoyl-5-*O*-((*t*-butyldiphenyl)-silyl)-α-D-arabinofuranosyl)-α-D-arabinofuranosyl)-5-*O*-(2,3-di-*O*-benzoyl-5-*O*-(2,3-di-*O*-benzoyl-5-*O*-((*t*-butyldiphenyl)-silyl)-α-D-arabinofuranosyl)-α-D-arabinofuranosyl)-α-D-arabinofuranosyl)-α-D-arabinofuranoside **28**

The above-mentioned supplementary method D from **27** (3.00 g, 1.65 mmol and HF.Py (70% HF basis, 0.47 g, 16.48 mmol) in anhydrous THF:Py (20:4 mL) afforded pent-4-enyl 2,3-di-*O*-benzoyl-5-*O*-(2-*O*-benzoyl-3-*O*-(2,3-di-*O*-benzoyl-α-D-arabinofuranosyl)-5-*O*-(2,3-di-*O*-benzoyl-α-D-arabino furanosyl)-α-D-arabinofuranosyl)-α-D-arabinofuranoside (1.66 g, 75%). $[\alpha]_D^{25}$ ($CHCl_3$, *c* 1.5): –15.3°; IR (cm^{-1}, $CHCl_3$): 3293, 3071, 2974, 1723, 1594, 1450, 1268, 1107, 717; ^1H NMR (399.78 MHz, $CDCl_3$): δ 1.63 – 1.79 (m, 2H), 2.07 – 2.22 (m, 2H), 2.72 (s, 2H), 3.37 (d, J = 3.0 Hz, 1H), 3.46 – 3.55 (m, 1H), 3.73 – 3.85 (m, 2H), 3.85 – 3.93 (m, 3H), 3.99 (ddd, J = 11.4, 7.2, 3.6 Hz, 2H), 4.17 (dd, J = 11.2, 4.5 Hz, 1H), 4.30 (q, J = 5.0 Hz, 1H), 4.38 (dq, J = 13.5, 4.7 Hz, 2H), 4.46 – 4.56 (m, 2H), 4.86 – 5.06 (m, 2H), 5.18 (dd, J = 5.0, 1.5 Hz, 1H), 5.21 (s, 1H), 5.25 (d, J = 4.5 Hz, 1H), 5.31 (s, 1H), 5.35 (s, 1H), 5.37 (s, 1H), 5.43 (d, J = 1.7 Hz, 1H), 5.45 (d, J = 1.2 Hz, 1H), 5.51 (d, J = 1.4 Hz, 1H), 5.57 (d, J = 1.2 Hz, 1H), 5.62 (d, J = 5.0 Hz, 1H), 5.80 (ddt, J = 16.9, 10.1, 6.7 Hz, 1H), 7.14 – 7.65 (m, 21H), 7.82 – 8.11 (m, 14H); ^{13}C NMR (100.53 MHz, $CDCl_3$): δ 28.7, 30.3, 62.6, 62.7, 64.9, 65.7, 66.7, 77.1, 77.9, 78.0, 80.7, 81.0, 81.5, 81.5, 81.8, 81.9, 82.7, 84.0, 84.1, 105.1, 105.3, 105.5, 105.6, 114.9, 128.3-129.9 (35C), 133.3, 133.4, 133.4, 133.4, 133.4, 133.4, 133.5, 138.1, 164.7, 165.1, 165.4, 165.5, 165.6, 166.1, 166.1; HRMS (ESI): m/z calcd for $[C_{74}H_{70}O_{24}+Na]^+$: 1365.4155; Found: 1365.4120.

This compound (1.50 g, 1.12 mmol) was subjected to supplementary method A using the glycosyl donor **8b** (1.77 g, 2.79 mmol), and $AuCl_3$ (47.42 mg, 0.16 mmol) in anhydrous CH_2Cl_2 (80 mL) as the starting material. Yield: 1.40 g, 50%. $[\alpha]_D^{25}$ ($CHCl_3$, *c* 0.7): +10.2°; IR (cm^{-1}, $CHCl_3$): 3071, 2928, 1726, 1591, 1454, 1265, 1108, 708; ^1H NMR (399.78 MHz, $CDCl_3$): δ 0.96 (s, 9H), 0.98 (s, 9H), 1.63 – 1.77 (m, 2H), 2.06 – 2.22 (m, 2H), 3.21 – 3.37 (m, 1H), 3.36 – 3.39 (m, 2H), 3.42 – 3.55 (m, 3H), 3.68 – 4.07 (m, 10H), 4.13 (dt, J = 10.6, 5.2 Hz, 3H), 4.30 – 4.58 (m, 5H), 4.84 – 5.07 (m, 1H), 5.19 (s, 1H), 5.22 (s, 1H), 5.31 (d, J = 3.4 Hz, 2H), 5.36 (s, 1H), 5.43 (d, J = 1.3 Hz, 1H), 5.46 (d, J = 1.2 Hz, 1H), 5.51 (d, J = 1.5 Hz, 2H), 5.52 (d, J = 1.3 Hz, 1H), 5.54 (d, J = 0.9 Hz, 1H), 5.55 – 5.63 (m, 4H), 5.79 (ddt, J = 16.9, 10.3, 6.7 Hz, 1H), 7.15 – 7.61 (m, 45H), 7.60 – 7.73 (m, 6H), 7.79 – 8.09 (m, 24H); ^{13}C NMR (100.53 MHz, $CDCl_3$): δ 19.2, 19.2, 26.7, 26.7, 26.7, 26.7, 26.7, 26.7, 28.7, 30.3, 63.1, 63.3, 65.6, 65.7, 65.8, 66.7, 66.7, 74.9, 75.1, 77.2, 81.3, 81.5, 81.7, 81.8, 81.9, 81.9, 82.0, 82.1, 82.4, 82.4, 82.5, 82.6, 82.6, 83.0, 83.1, 105.3, 105.5, 105.8, 105.9, 105.9, 106.0, 114.8, 127.6-129.9 (75C), 133.0, 133.1, 133.1, 133.2, 133.2, 133.2, 133.2, 133.2, 133.2, 133.2, 133.4, 135.6 (4C), 138.1, 164.8, 165.0, 165.0, 165.1, 165.3, 165.4, 165.4, 165.5, 165.5, 165.5, 165.5; HRMS (ESI): m/z calcd for $[C_{144}H_{138}O_{36}Si_2+Na]^+$: 2522.8438; Found: 2522.8486.

Propargyl 3-*O*-benzoyl-5-*O*-(2-*O*-benzoyl-3-*O*-(2,3-di-*O*-benzoyl-5-*O*-(2,3-di-*O*-benzoyl-5-*O*-((*t*-butyldiphenyl)silyl)-α-D-arabinofuranosyl)-α-D-arabinofuranosyl)-5-*O*-(2,3-di-*O*-benzoyl-5-*O*-(2,3-di-*O*-benzoyl-5-*O*-((*t*-butyldiphenyl)silyl)-α-D-arabinofuranosyl)-α-D-arabinofuranosyl)-α-D-arabinofuranosyl)-α-D-arabinofuranoside 1,2-*O*-orthobenozoate **4**

This compound is prepared using the above delineated supplementary method C on pentenyl glycosides **28** (1.25 g, 0.50 mmol), Br_2 (87.87 mg, 0.55 mmol), propargyl alcohol (70.06 mg, 1.25 mmol), and 2,6-lutidine (0.15 g, 1.25 mmol) in anhydrous CH_2Cl_2 (10 mL) as the starting material.

Yield: 1.06 g, 86%. $[\alpha]_D^{25}$ ($CHCl_3$, *c* 1.2): –31.8 °; IR (cm^{-1}, $CHCl_3$): 3297, 3067, 2926, 1726, 1596, 1456, 1265, 1107, 707; ^1H NMR (399.78 MHz, $CDCl_3$): δ 0.99 (s, 9H), 0.99 (s, 9H), 2.36 (t, J = 2.3 Hz, 1H), 3.47 (dd, *J* = 10.2, 6.9 Hz, 1H), 3.62 (d, *J* = 9.5 Hz, 1H), 3.72 – 3.79 (m, 2H), 3.82 – 3.86 (m, 1H), 3.87 – 3.96 (m, 7H), 4.15 (dd, J = 11.1, 4.1 Hz, 1H), 4.19 (dd, J = 11.3, 3.4 Hz, 1H), 4.30-4.32 (m, 1H), 4.41-4.42 (m, 1H), 4.46-4.50 (m, 5H), 4.99 (s, 1H), 5.06 (d, J = 4.3 Hz, 1H), 5.23 (s, 1H), 5.26 (s, 1H), 5.33 (s, 1H),5.35 (s, 1H), 5.44 (s, 1H), 5.45 (s, 1H), 5.52 (s, 1H), 5.50 (s, 1H), 5.54 (s, 1H), 5.59 (s, 1H), 5.61 (s, 1H), 5.62 (s, 1H),5.60 (s, 1H), 5.67 (d, J = 4.5 Hz, 1H), 6.28 (d, J = 4.3 Hz, 1H), 7.21-8.02 (m, 75H); ^{13}C NMR (100.53 MHz, $CDCl_3$): δ 19.2 (2C), 26.7 (6C), 52.0, 65.4 (2C), 65.6 (2C), 66.6 (2C), 76.8, 76.8, 77.0, 77.2 (3C), 77.7, 79.3, 81.2, 81.4, 81.5, 82.0, 82.0, 82.5, 82.6, 82.8, 83.1 (2C), 84.8, 85.3, 105.3, 105.5, 105.8, 105.9, 106.1, 106.6, 122.5, 126.4-135.6 (90C), 164.9, 165.0 (2C), 165.1, 165.2, 165.4 (3C), 165.5, 165.6; HRMS (ESI): m/z calcd for $[C_{142}H_{132}O_{36}Si_2+Na]^+$: 2492.7968; Found: 2492.7959.

Pent-4-enyl 2,3-di-*O*-benzoyl-5-*O*-(2-*O*-benzoyl-3-*O*-(2-*O*-acetyl-3,5-di-*O*-benzoyl-α-D-arabinofuranosyl)-5-*O*-(2-*O*-acetyl-3,5-di-*O*-benzoyl-α-D-arabinofuranosyl)-α-D-arabinofuranosyl)-α-D-arabinofuranoside **29**

This compound is prepared using the above mentioned supplementary method A using aglycon **26** (1.15 g, 1.74 mmol), **8c** (1.90 g, 4.34 mmol) as starting materials, and $AuCl_3$ (73.69 mg, 0.24 mmol), AgOTf (61.4 mg, 0.24 mmol) in anhydrous CH_2Cl_2 (100 mL). Yield: 1.19 g, 48%. $[\alpha]_D^{25}$ ($CHCl_3$, *c* 1.0): +24.4°; IR (cm^{-1}, $CHCl_3$): 3071, 2927, 1716, 1600, 1451, 1267, 1100, 753; ^1H NMR (400.13 MHz, $CDCl_3$): δ 1.63 – 1.84 (m, 2H), 1.96 (s, 3H), 1.99 (s, 3H), 2.12 – 2.26 (m, 2H), 3.54 (dt, J = 9.6, 6.1 Hz, 1H), 3.80 (dt, J = 9.5, 6.6 Hz, 1H), 3.87 – 4.00 (m, 2H), 4.10 (dd, J = 11.5, 3.9 Hz, 1H), 4.18 (dd, J = 11.2, 4.4 Hz, 1H), 4.39 – 4.79 (m, 9H), 4.90 – 5.10 (m, 2H), 5.25 (s, 1H), 5.28 (s, 1H), 5.35 (s, 2H),

5.35 (s, 1H), 5.38 (d, J = 4.3 Hz, 1H), 5.41 (s, 1H), 5.44 (s, 1H), 5.48 (s, 1H), 5.55 (s, 1H), 5.67 (d, J = 4.9 Hz, 1H), 5.83 (ddt, J = 16.9, 10.2, 6.6 Hz, 1H), 7.10 – 7.65 (m, 21H), 7.79 – 8.33 (m, 14H); ^{13}C NMR (100.62 MHz, $CDCl_3$): δ 20.5, 20.5, 28.6, 30.2, 63.4, 63.5, 65.4, 65.5, 66.6, 77.4, 77.6, 77.8, 80.8, 81.1, 81.2, 81.2, 81.5, 81.7, 81.8, 81.9, 82.6, 105.4, 105.5, 105.6, 105.7, 114.8, 128.2-128.5(14C), 128.8-129.1(7C), 129.6-129.7(14C), 133.0, 133.0, 133.3, 133.3, 133.3, 133.3, 133.4, 138.0, 165.3, 165.4, 165.5, 165.5, 165.6, 165.8, 166.0, 169.1, 169.3. HRMS (ESI): m/z calcd for $[C_{78}H_{74}O_{26}+Na]^+$: 1449.4366; Found: 1449.4391.

Prop-2-yn-1-yl 3-*O*-benzoyl-5-*O*-(2-*O*-benzoyl-3-*O*-(2-O-acetyl 3,5-di-*O*-benzoyl-α-D-arabinofuranosyl)-5-*O*-(2-*O*-acetyl 3,5-di-*O*-benzoyl-α-D-arabinofuranosyl)-α-D-arabinofuranosyl)-α-D-arabinofuranoside 1,2-*O*-orthobenzoate **2**

This compound is prepared using the above mentioned supplementary method C using pentenyl glycosides **29** (1.50 g, 1.05 mmol), Br_2 (0.19 g, 1.16 mmol), propargyl alcohol (0.15 g, 2.63 mmol), and 2,6-lutidine (0.32 g, 2.63 mmol) in anhydrous CH_2Cl_2 (15 mL). Yield: 1.29 g, 88%. $[\alpha]_D^{25}$ ($CHCl_3$, c 1.0): +54.8°; IR (cm^{-1}, $CHCl_3$): 3070, 2931, 1716, 1600, 1455, 1267, 1110, 861; ^{1}H NMR (500.00 MHz, $CDCl_3$): δ 2.02 (s, 6H), 2.40 (bs, 1H), 3.45 – 3.81 (m, 3H), 3.82 – 4.17 (m, 3H), 4.33 (d, J = 20.1 Hz, 1H), 4.42 – 4.76 (m, 7H), 5.01 (s, 1H), 5.09 (d, J = 3.9 Hz, 1H), 5.16 (s, 1H), 5.30 (s, 1H), 5.33 (s, 2H), 5.39 (s, 2H), 5.21 – 5.66 (m, 2H), 5.47 (s, 1H), 6.31 (d, J = 4.0 Hz, 1H), 7.08 – 7.73 (m, 21H), 7.79 – 8.30 (m, 14H); ^{13}C NMR (125.77 MHz, $CDCl_3$): δ 20.6, 20.6, 52.0, 54.1, 63.6, 65.3, 66.6, 73.7, 74.9, 77.6, 77.7, 79.3, 80.5, 80.8, 81.1, 81.4, 81.5, 81.5, 82.5, 84.8, 85.4, 105.4, 105.5, 105.9, 106.6, 122.5, 126.4, 128.2- 129.9 (34C), 133.0, 133.1, 133.3, 133.3, 133.3, 133.5, 134.3, 165.2, 165.4, 165.6, 165.7, 165.9, 166.1, 169.3, 169.3. HRMS (ESI): m/z calcd for $[C_{76}H_{68}O_{26}+Na]^+$: 1419.3897; Found: 1419.3891.

[3-(Methoxycarbonyl) propyl] 2,3-di-*O*-benzoyl-5-*O*-(2,3-di-*O*-benzoyl-5-*O*-(2,3-di-*O*-benzoyl-5-*O*-(2,3-di-*O*-benzoyl-5-*O*-(2,3-di-*O*-benzoyl-5-*O*-(2,3-di-*O*-benzoyl-5-*O*-((tertbutyldiphenyl)-silyl)-α-D-arabinofuranosyl)-α-D-arabinofuranosyl)-α-D-arabinofuranosyl)-α-D-arabinofuranosyl)-α-D-arabinofuranosyl)-6-*O*-(2,3,5,6-tetra-*O*-acetyl-β-D-galactofuranosyl)-β-D-galactofuranoside **30**

This compound is prepared using the above-mentioned supplementary method A using aglycon **7** (0.7 g, 0.60 mmol) and **6** (1.00 g, 0.60 mmol), $AuCl_3$ (12.82 mg, 0.04 mmol), and AgOTf (10.86 mg, 0.04 mmol) in anhydrous CH_2Cl_2 (40 mL) as the starting material. Yield: 1.17 g, 70%; $[\alpha]_D^{25}$ ($CHCl_3$, *c* 1.5): +15.3 ^{0}; IR(cm^{-1}, $CHCl_3$): 3071, 2974, 1721, 1601, 1450, 1268, 1107, 757; ^{1}H NMR (399.78 MHz, $CDCl_3$): δ 0.96 (s, 9H), 1.72 (s, 3H), 1.85 (s, 3H), 1.90 (s, 3H), 1.95 (s, 3H), 2.00-2.04 (m, 2H), 2.34 – 2.45 (m, 2H), 3.29 – 3.59 (m, 2H), 3.57 (s, 3H), 3.69 – 3.98 (m, 10H), 4.12 (bs, 6H), 4.20 – 4.46 (m, 6H), 4.54 (s, 3H), 4.91 (d, J = 26.7 Hz, 1H), 5.15 (d, J = 10.1 Hz, 1H), 5.21 (s, 1H), 5.33 (d, J = 8.3 Hz, 5H), 5.39 – 5.72 (m, 13H), 7.03 – 7.56 (m, 42H), 7.63 (s, 4H), 7.72 – 8.15 (m, 24H); ^{13}C NMR (100.53 MHz, $CDCl_3$): δ 19.3, 20.5, 20.6, 20.7, 20.8, 22.9, 24.9, 26.8, 26.8, 26.8, 29.6, 29.8, 30.7, 51.2, 51.6, 62.8, 63.4, 63.4, 65.8, 65.8, 65.9, 66.4, 69.5, 72.2, 76.4, 77.2, 77.2, 80.5, 81.4, 81.5, 81.6, 81.6, 81.9, 82.0, 82.1, 82.1, 82.1, 82.1, 82.1, 82.1, 82.2, 83.2, 83.2, 104.4, 105.4, 106.0, 106.0, 106.0, 106.2, 127.7-128.6 (27C), 129.0, 129.1, 129.1, 129.1, 129.1, 129.1, 129.1, 129.2, 129.2, 129.3, 129.3, 129.3, 129.7-130.0 (23C), 133.0, 133.0, 133.1, 133.2, 133.2, 133.2, 133.3, 133.4, 133.4, 133.4, 133.5, 133.5, 133.6, 133.7, 135.7 (8C), 165.2, 165.2, 165.2, 165.3, 165.4, 165.5, 165.5, 165.6, 165.6, 165.7, 165.7, 165.7, 169.6, 170.1, 170.1, 170.5, 173.8; HRMS (ESI): m/z calcd for $[C_{150}H_{144}O_{49}Si+Na]^+$: 2779.8443; Found: 2779.8431.

[3-(Methoxycarbonyl) propyl] 2,3-di-*O*-benzoyl-5-*O*-(2,3-di-*O*-benzoyl-5-*O*-(2,3-di-*O*-benzoyl-5-*O*-(2,-3-di-*O*-benzoyl-5-*O*-(2,3-di-*O*-benzoyl-5-*O*- (2,3-di-*O*-benzoyl-α-D-arabinofuranosyl)-α-D-

arabinofuranosyl)-α-D-arabinofuranosyl)-α-D-arabinofuranosyl)-α-D-arabinofuranosyl)-6-*O*-(2,3,5,6-tetra-*O*-acetyl-β-D-galactofuranosyl)-β-D-galactofuranoside **5**

This compound is prepared using the aforementioned supplementary method D utilizing **30** (1.00 g, 0.36 mmol and HF.Py (70% HF basis, 41.44 mg, 1.45 mmol) in 20 mL of anhydrous THF:Py (4:1). Yield: 0.78 g, 85%. $[\alpha]_D^{25}$ ($CHCl_3$, *c* 1.0): +3.9 °; IR (cm^{-1}, $CHCl_3$): 3333, 2925, 1725, 1591, 1451, 1263, 1108, 711; ^{1}H NMR (399.78 MHz, $CDCl_3$): δ 1.91 (s, 3H), 1.96 (s, 3H), 2.00 (s, 3H), 1.95-2.01 (m, 2H), 2.07 (s, 3H), 2.49 (t, J = 7.5 Hz, 2H), 3.56 (dt, J = 9.8, 6.1 Hz, 1H), 3.75-4.62 (m, 19H), 4.12-4.62 (m, 7H), 4.95 (dd, J = 5.9, 2.0 Hz, 1H), 5.00 (d, J = 2.2 Hz, 1H), 5.05 (s, 1H), 5.24 (s, 1H), 5.35 (dt, J = 7.6, 3.9 Hz, 1H), 5.40 (m, 6H), 5.48 (d, J = 1.4 Hz, 1H), 5.60-5.65 (m, 4H), 5.69 (d, J = 4.9 Hz, 1H), 5.60-5.76 (m, 5H), 5.74 (s, 1H), 7.21-7.59 (m, 36H),7.84-8.07 (m, 24H); ^{13}C NMR (100.53 MHz, $CDCl_3$): δ 20.4 (2C), 20.6, 20.7, 24.8, 30.6, 51.5, 62.2, 62.6, 66.5, 65.6, 65.7, 66.0, 66.3, 66.6, 69.1, 75.2, 76.2, 76.6, 77.0, 77.1 (2C), 77.2, 77.6, 79.9, 81.0, 81.4, 81.5 (3C), 81.6, 81.9, 82.2 (2C), 82.2 (2C), 82.3, 83.5, 105.3, 105.5, 105.7 (3C), 105.8, 106.7, 128.2-133.4 (72C), 165.0 (4C), 165.2, 165.4, 165.5 (3C), 165.6 (2C), 166.0, 169.3, 169.9 (2C), 170.4, 173.6; HRMS (ESI): m/z calcd for $[C_{134}H_{126}O_{49}+Na]^+$: 2542.7299; Found: 2542.7332.

[3-(Methoxycarbonyl) propyl] 2,3-di-*O*-benzoyl-5-*O*-(2,3-di-*O*-benzoyl-5-*O*-(2,3-di-*O*-benzoyl-5-*O*-(2,3-di-*O*-benzoyl-5-*O*-(2,3-di-*O*-benzoyl-5-*O*-(2,3-di-*O*-benzoyl-5-*O*-(2,3-di-O-benzoyl-5-*O*-(2-*O*-benzoyl-3-*O*-(2,3-di-*O*-benzoyl-5-*O*-(2,3-di-*O*-benzoyl-5-*O*-(*t*-butyldiphenylsilyl)-α-D-arabinofuranosyl)-α-D-arabinofuranosyl)-5-*O*-(2,3-di-*O*-benzoyl-5-*O*-(2-,3-di-*O*-benzoyl-5-*O*-(*t*-butyldiphenylsilyl)-α-D-arabinofuranosyl)-α-D-arabinofuranosyl)-α-D-arabinofuranosyl)-α-D-arabinofuranosyl)-α-D-arabinofuranosyl)-α-D-arabinofuranosyl)-α-D-arabinofuranosyl)-α-D-arabinofuranosyl)-α-D-arabinofuranosyl)-6-*O*-(2,3,5,6-tetra-*O*-acetyl-β-D-galactofuranosyl)-β-D-galactofuranoside **31**

This compound is prepared utilizing the aforementioned supplementary method A using aglycon **5** (100 mg, 39.70 μmol) and glycosyl donor **28** (98.1 mg, 39.70 μmol), NIS (26.80 mg, 119.11 μmol), and AgOTf (1.79 mg, 11.91 μmol) in anhydrous CH_2Cl_2 (20 mL). Yield: 42.46 mg, 24% and; (**b**) using aglycon **5** (0.51 g, 0.20 mmol), glycosyl donor **4** (0.50 g, 0.20 mmol), AuCl3 (6.14 mg, 0.02 mmol), and AgOTf (3.64 mg, 0.01 mmol) in anhydrous CH_2Cl_2 (20 mL) as the starting material. Yield: (0.58 g, 65%); $[\alpha]_D^{25}$ ($CHCl_3$, *c* 0.8): +12.9°; IR (cm^{-1}, $CHCl_3$): 3060, 2927, 1726, 1591, 1452, 1269, 1108, 710; ^1H NMR (399.78 MHz, $CDCl_3$): δ 0.99 (s, 9H), 0.99 (s, 9H), 1.90 (s, 3H), 1.96 (s, 3H), 1.99 (s, 3H), 1.95-2.01 (m, 2H), 2.07 (s, 3H), 2.49 (dd, J = 7.9, 7.1 Hz, 2H), 3.55 (dt, J = 9.8, 6.1 Hz, 1H), 3.66 (s, 3H), 3.72-4.03(m, 14H), 3.78 (dt, J = 9.9, 5.8 Hz, 1H), 4.09-4.20 (m, 7H), 4.10-4.20 (m, 2H), 4.26-4.31 (m, 2H), 4.37-4.61 (m, 13H), 4.38-4.61 (m, 2H), 4.94 (dd, J =5.9, 2.0 Hz, 1H), 4.99 (d, J =2.1 Hz, 1H) 5.05 (s, 1H), 5.21 (s, 1H), 5.24 (s, 1H), 5.29 (s, 1H), 5.30 (s, 1H), 5.32-5.35 (m, 1H), 5.36 (s, 1H), 5.37 (s, 1H), 5.38 (s, 2H), 5.44 (d, 1.0 Hz, 1H), 5.47 (d, J = 1.1 Hz, 1H), 5.49 (s, 1H), 5.49 (s, 1H), 5.50 (d, 1.5 Hz 1H), 5.51 (d, 1.3 Hz, 1H), 5.52(m, 1H), 5.52(d, 1.1 Hz 1H), 5.58-5.64 (m, 7H), 5.54 (d, J = 0.9 Hz, 1H), 5.58-5.64 (m, 8H), 5.68 (d, J = 4.8 Hz, 1H), 5.72-5.75 (m, 2H), 5.73 (s, 1H), 7.15-8.8.07 (m, 135H); ^{13}C NMR (100.53 MHz, $CDCl_3$): δ 19.2 (2C), 20.5 (2C), 20.6, 20.8, 26.7 (6C), 29.7, 30.6, 51.5, 62.7, 63.1, 63.2, 65.4 (2C), 65.5, 65.6 (2C), 65.7 (3C), 66.4, 66.6, 69.1, 75.2, 76.3, 76.7, 77.0 (4C), 77.1 (2C), 77.2 (4C), 80.0, 80.3, 81.0, 81.2, 81.4 (4C), 81.5, 81.6 (4C), 82.0 (2C), 82.0 (4C), 82.1, 82.2, 82.3 (2C), 82.5, 82.6 (2C), 82.9, 83.1, 105.2, 105.4, 105.6, 105.9 (8C), 106.1, 106.7, 127.6-135.6 (162C), 164.7, 164.9, 165.0 (4C), 165.1 (2C), 165.2 (3C), 165.3, 165.4 (2C), 165.5 (6C), 165.6 (3C), 169.3, 170.0, 170.0, 170.4, 173.7; HRMS (ESI): m/z calcd for $[C_{273}H_{254}O_{84}Si_2+Na]^+$: 4956.5107; Found: 4956.5207.

[3-(Methoxycarbonyl) propyl] 2,3-di-*O*-benzoyl-5-*O*-(2,3-di-*O*-benzoyl-5-*O*-(2,3-di-*O*-benzoyl-5-*O*-(2,-3-di-*O*-benzoyl-5-*O*-(2,3-di-*O*-benzoyl-5-*O*-(2,3-di-*O*-benzoyl-5-*O*-(2,3-di-*O*-benzoyl-5-*O*-(2-*O*-benzoyl-3-*O*-(2,3-di-*O*-benzoyl-5-*O*-(2,3-di-*O*-benzoyl-α-D-arabinofuranosyl)-α-D-arabinofuranosyl)-5-

O-(2,3-di-*O*-benzoyl-5-*O*-(2,3-di-*O*-benzoyl-α-D-arabinofuranosyl)-α-D-arabinofuranosyl)-α-D-arabinofuranosyl)-α-D-arabinofuranosyl)-α-D-arabinofuranosyl)-α-D-arabinofuranosyl)-α-D-arabinofuranosyl)-α-D-arabinofuranosyl)-α-D-arabinofuranosyl)-6-*O*-(2,3,5,6-tetra-*O*-acetyl-β-D-galactofuranosyl)-β-D-galactofuranoside **3**

This compound is prepared using the aforementioned supplementary method D using **31** (0.5 g, 0.10 mmol and HF.Py (70% HF basis, 20.77 mg, 0.81 mmol) in anhydrous THF:Py (5:1 mL). Yield: 0.38 g, 85%. $[\alpha]_D^{25}$ ($CHCl_3$, *c* 0.8): +12.9°; IR (cm^{-1}, $CHCl_3$): 3060, 2927, 1726, 1591, 1452, 1269, 1108, 710; 3420, 3029, 2925, 1600, 1454, 1361, 1065, 741; ^1H NMR (399.78 MHz, $CDCl_3$): δ 1.89 (s, 3H), 1.95 (s, 3H), 1.90 – 2.04 (m, 2H), 1.98 (s, 3H), 2.06 (s, 3H), 2.48 (t, J = 7.5 Hz, 2H), 3.54 (dt, J = 9.8, 6.2 Hz, 2H), 3.64 (s, 3H), 3.68 – 4.07 (m, 18H), 4.06 – 4.21 (m, 7H), 4.24 – 4.32 (m, 2H), 4.32 – 4.37 (m, 2H), 4.38 – 4.54 (m, 7H), 4.52 – 4.65 (m, 6H), 4.94 (dd, J = 5.9, 2.0 Hz, 1H), 4.98 (d, J = 2.2 Hz, 1H), 5.04 (s, 1H), 5.23 (s, 1H), 5.27 (s, 1H), 5.33 (s, 1H), 5.35 (s, 2H), 5.37 (s, 2H), 5.37 – 5.39 (m, 5H), 5.42 (d, J = 1.4 Hz, 1H), 5.45 (d, J = 1.2 Hz, 1H), 5.48 (d, J = 1.5 Hz, 1H), 5.50 (d, J = 1.4 Hz, 1H), 5.52 (s, 1H), 5.53 (s, 1H), 5.55 – 5.57 (m, 3H), 5.58 (s, 1H), 5.60 – 5.64 (m, 3H), 5.62 (s, 8H), 5.67 (d, J = 4.9 Hz, 1H), 5.69 – 5.77 (m, 3H), 7.08 – 7.67 (m, 69H), 7.73 – 8.17 (m, 46H); ^{13}C NMR (100.53 MHz, $CDCl_3$): δ 20.5, 20.5, 20.6, 20.7, 30.6, 31.9, 51.5, 62.1, 62.2, 62.7, 64.8, 65.4, 65.5, 65.6, 65.6, 65.6, 65.7, 66.3, 66.6, 69.1, 69.1, 75.2, 75.2, 76.3, 76.3, 77.0, 77.0, 77.1, 77.1, 77.1, 77.2, 77.2, 77.2, 77.4, 77.5, 79.9, 80.5, 81.0, 81.1, 81.2, 81.4, 81.4, 81.4, 81.4, 81.4, 81.5, 81.5, 81.5, 81.5, 81.6, 81.9, 82.0, 82.0, 82.2, 82.2, 82.3, 82.4, 82.8, 83.7, 83.8, 83.8, 83.8, 105.3, 105.4, 105.5, 105.5, 105.5, 105.6, 105.7, 105.8, 105.8, 105.8, 105.8, 105.9, 106.7, 128.2-133.5 (138C), 164.7, 164.9, 165.0, 165.0, 165.0, 165.0, 165.0, 165.0, 165.0, 165.0, 165.0, 165.0, 165.1, 165.2, 165.2, 165.2, 165.4, 165.5, 165.5, 165.5, 165.5, 165.5, 165.6, 165.6, 165.6, 165.8, 165.9, 169.3, 169.9, 170.0, 170.4, 173.7; HRMS (ESI) : m/z calcd for $[C_{241}H_{218}O_{84}+Na]^+$: 4480.2752; Found: 4480.2869.

[3-(Methoxycarbonyl)-propyl] 2,3-di-*O*-benzoyl-5-*O*-(2,3-di-*O*-benzoyl-5-*O*-(2,3-di-*O*-benzoyl-5-*O*-(2,3-di-*O*-benzoyl-5-*O*-(2,3-di-*O*-benzoyl-5-*O*-(2,3-di-*O*-benzoyl-5-*O*-(2,3-di-*O*-benzoyl-5-*O*-(2-*O*-benzoyl-3-*O*-(2,3-di-*O*-benzoyl-5-*O*-(2,3-di-*O*-benzoyl-5-*O*-(2,3-di-*O*-benzoyl-5-*O*-(2-*O*-benzoyl-3-*O*-(2-*O*-acetyl-3 ,5-di-*O*-benzoyl-α-D-arabinofuranosyl)-5-*O*-(2-*O*-acetyl-3,5-di-*O*-benzoyl-α-D-arabinofuranosyl)-α-D-arabinofuranosyl)-α-D-arabinofuranosyl)-α-D-arabinofuranosyl)-α-D-arabinofuranosyl)-5-*O*-(2,3-di-*O*-benzoyl-5-*O*-(2,3-di-*O*-benzoyl-5-*O*-(2,3-di-*O*-benzoyl-5-*O*-(2-*O*-benzoyl-3-*O*-(2-*O*-acetyl-3,5-di-*O*-benzoyl-α-D-arabinofuranosyl)-5-*O*-(2-*O*-acetyl-3,5-di-*O*-benzoyl-α-D-arabinofuranosyl)-α-D-arabinofuranosyl)-α-D-arabinofuranosyl)-α-D-arabinofuranosyl)-α-D-arabinofuranosyl)-α-D-arabinofuranosyl)-α-D-arabinofuranosyl)-α-D-arabinofuranosyl)-α-D-arabinofuranosyl)-α-D-arabinofuranosyl)-α-D-arabinofuranosyl)-α-D-arabinofuranosyl)-6-*O*-(2,3,5,6-tetra-*O*-acetyl-β-D-galactofuranosyl)-β-D-galactofuranoside **32**

This compound is prepared using the aforementioned supplementary method A using aglycon **3** (0.15 g, 0.04 mmol) and donor **2** (0.12 g, 0.08 mmol), $AuCl_3$ (1.43 mg, 4.71 µmol), and AgOTf (1.21 mg, 4.71 µmol) in anhydrous CH_2Cl_2 (20 mL). Yield: 0.17 g, 71%. $[\alpha]_D^{25}$ ($CHCl_3$, *c* 0.8): +38.2°. IR (cm^{-1}, $CHCl_3$): 3056, 2926, 1729, 1590, 1450, 1270, 1108, 709; ^{1}H NMR (400.13 MHz, $CDCl_3$): δ 1.56 (dd, J = 16.9, 9.3 Hz, 2H), 1.92 (s, 3H), 1.94 (s, 3H), 1.97 (s, 3H), 1.98 (s, 3H), 2.01 (s, 3H), 2.01 (s, 2x3H), 2.09 (s, 3H), 2.50 (t, J = 7.5 Hz, 2H), 3.68 (s, 3H), 3.74 – 3.95 (m, 20H), 3.97 – 4.24 (m, 16H), 4.31 (dt, J = 8.9, 4.5 Hz, 2H), 4.40-4.72 (m, 30H), 4.97 (d, J = 5.7 Hz, 1H), 5.01 (d, J = 2.2 Hz, 1H), 5.07 (s, 1H), 5.21 (s, 2H), 5.25 (s, 2x1H), 5.27 (d, J = 3.1 Hz, 2H), 5.30 (m, 5H), 5.34 (m, 4H), 5.36 (m, 3H), 5.38 (m, 2H), 5.34-5.40 (m, 9H), 5.47 (m, 3H), 5.51 (m, 4H), 5.55 (d, J = 3.0 Hz, 3H), 5.59 (s, 1H), 5.45 – 5.68 (m, 7H), 5.61 (m, 5H), 5.64 (m, 4H), 5.70 (d, J = 4.6 Hz, 1H), 5.71 – 5.81 (m, 2H), 7.10 – 7.62 (m, 111H), 7.74 – 8.15 (m, 74H); ^{13}C NMR (100.61 MHz, $CDCl_3$): δ 20.4, 20.5, 20.6, 20.6, 20.7, 20.7, 23.8, 23.8, 28.4, 30.6, 51.5, 62.7-66.4 (22C), 69.2-77.8 (22C), 80.0-82.8 (43C), 105.2, 105.4 (2C), 105.5 (3C), 105.6, 105.7 (4C), 105.9 (8C), 106.0, 106.7, 128.2-133.3 (222C), 164.8,-

166.1 (37C), 169.1, 169.1, 169.3, 169.3, 169.3, 170.0, 170.0, 170.4, 173.7; HRMS (MALDI-TOF): Calculated m/z for $[C_{387}H_{346}O_{134}+Na]^+$: 7163.0292, Found: 7170.6265 (in linear mid mode).

[3-(Methoxycarbonyl)-propyl] 5-*O*-(5-*O*-(5-*O*-(5-*O*-(5-*O*-(5-*O*-(5-*O*-(3-*O*-(5-*O*-(5-*O*-(5-*O*-(3-*O*-(-α-D-arabinofuranosyl)-5-*O*-(α-D-arabinofuranosyl)-α-D-arabinofuranosyl)-α-D-arabinofuranosyl)-α-D-arabinofuranosyl)-α-D-arabinofuranosyl)-5-*O*-(5-*O*-(5-*O*-(5-*O*-(3-*O*-(α-D-arabinofuranosyl)-5-*O*-(α-D-arabinofuranosyl)-α-D-arabinofuranosyl)-α-D-arabinofuranosyl)-α-D-arabinofuranosyl)-α-D-arabinofuranosyl)-α-D-arabinofuranosyl)-α-D-arabinofuranosyl)-α-D-arabinofuranosyl)-α-D-arabinofuranosyl)-α-D-arabinofuranosyl)-α-D-arabinofuranosyl)-α-D-arabinofuranosyl)-6-*O*-(β-D-galactofuranosyl)-β-D-galactofuranoside **1**

$[\alpha]_D^{25}$ (H_2O, *c* 1.0): +123.8°. ^1H NMR (600.40 MHz, D_2O): δ 1.78 (m, 2H), 2.25 (t, J = 7.4 Hz, 1H), 2.30 (t, J = 7.3 Hz, 1H), 3.33-4.30 (m, 112 H), 4.85-5.24 (m, 21 H); ^{13}C NMR (150.97 MHz, D_2O): δ 24.2, 30.6, 52.1, 61.1(5C), 62.8, 66.2-67.3(16C), 70.7(2C), 76.5-76.8(15C), 79.0-79.1(4C), 80.7-81.2(18C), 81.5-82.5(20C), 83.8-84.0(6C), 106.8-107.7(21C), 176.9; HRMS (MALDI-TOF): Calculated m/z for $[C_{112}H_{182}O_{89}+Na]^+$: 2974.9647, Found: 2974.2810.

Supplementary References

1. Thadke, S. A., Mishra, B., Hotha, S. Gold(III)-catalyzed glycosidations for 1,2-*trans* and 1,2-*cis* furanosides. *J. Org. Chem.* **79**, 7358-7371 (2014).
2. Lu, J., Fraser-Reid, B. Preparation, properties, and applications of *n*-pentenyl arabinofuranosyl donors. *Org. Lett.* **6**, 3051-3054 (2004).

3. Thadke, S. A., Hotha, S. Efficient synthesis of oligosaccharyl 1,2-*O*-orthoesters from *n*-pentenyl glycosides and application to the pentaarabinofuranoside of the mycobacterial cell surface. *Org. Biomol. Chem.* **12**, 9914-9920 (2014).
4. Hotha, S., Sureshkumar, G. Propargyl 1,2-orthoesters as glycosyl donors: stereoselective synthesis of 1,2-*trans* glycosides and disaccharides. *Tetrahedron Lett.* **48**, 6564-6568 (2007).
